# Nitrogen Radical Photocatalysed Reduction of Nitrous Oxide

**DOI:** 10.1002/anie.9521232

**Published:** 2026-08-23

**Authors:** Reece Lister‐Roberts, Agamemnon E. Crumpton, Meera Mehta

**Affiliations:** ^1^ Department of Chemistry University of Oxford Oxford UK

**Keywords:** main group catalysis, nitrogen radicals, nitrous oxide, photochemistry

## Abstract

Nitrous oxide (N_2_O) represents one of the most potent greenhouse gases, with a global warming potential of approximately 300× that of CO_2_. Yet protocols for its reduction to benign products such as dinitrogen (N_2_) remain comparatively rare. In this study, we report a nitrogen‐based catalyst that enables the photochemical reduction of N_2_O to N_2_ using pinacol borane as the reductant. Mechanistic investigations indicate that the active catalytic species operates via an unprecedented N(0)/N(–1) redox cycle. This work represents the first metal‐free homogeneous photocatalytic protocol for such a process.

## Graphical abstract

This work offers the first example of reduction of the potent greenhouse gas N_2_O to the benign products N_2_ and O(Bpin)_2_ using a nitrogen‐based catalyst. The active catalyst is shown to be a nitrogen‐based radical which operates under photocatalytic conditions. Experimental and computational studies give insights into the role of visible light in the reduction.

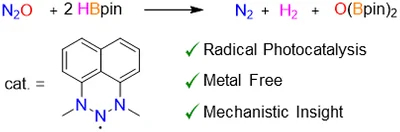

## 1 Introduction

Mitigating the detrimental impact of greenhouse gases on the climate has been a global challenge motivating academics, engineers, policymakers, philanthropists, and industry over the last several decades. While much attention has been paid to carbon dioxide (CO_2_), nitrous oxide (N_2_O) has largely been ignored despite having an estimated global warming potential 300× greater than that of CO_2_ and a lifetime of approximately 114 years [1, 2, 3]. The leading approach to N_2_O degradation is to facilitate oxygen atom transfer, producing benign dinitrogen (N_2_)—a process adopted by nature during microbial denitrification [4]. Traditionally, this degradation chemistry has been mediated by transition metal‐based catalysts, including precious metals [5, 6, 7, 8, 9, 10, 11]. Depleting reserves of these metals have led to growing fiscal and environmental costs associated with their use and increasingly insecure supply chains [12, 13]. In order to move to more sustainable processes there has been growing interest in developing organo‐main‐group catalysts, including for N_2_O abatement chemistry.

Early studies demonstrated the stoichiometric capture and cleavage of the N─O bond in N_2_O using various main group compounds [14, 15, 16, 17]. However, only very recently have catalytic protocols been developed in the field (Figure 1a). In 2019, Cantat et al. developed a catalytic system in which fluoride acts as a (pre‐)catalyst for the silylation of N_2_O [18]. In 2020, Cornella and coworkers reported the fastest main group system to date for N_2_O reduction, based on a Bi(I) species that accesses a Bi(I)/Bi(III) redox cycle [19]. Subsequently, Mo et al. expanded this work to Group 14 using a mixed Ge/Sn system which cooperatively facilitates N_2_O reduction [20]. Previously, we have shown that a range of low‐valent clusters based on the heavy Group 15 elements phosphorus, arsenic, and antimony could mediate the reduction of N_2_O to N_2_ [21]. Paradies et al. and Dong et al. later provided further examples of main group N_2_O reduction at phosphorus and Sn/Ge, respectively [22, 23].

**FIGURE 1:**
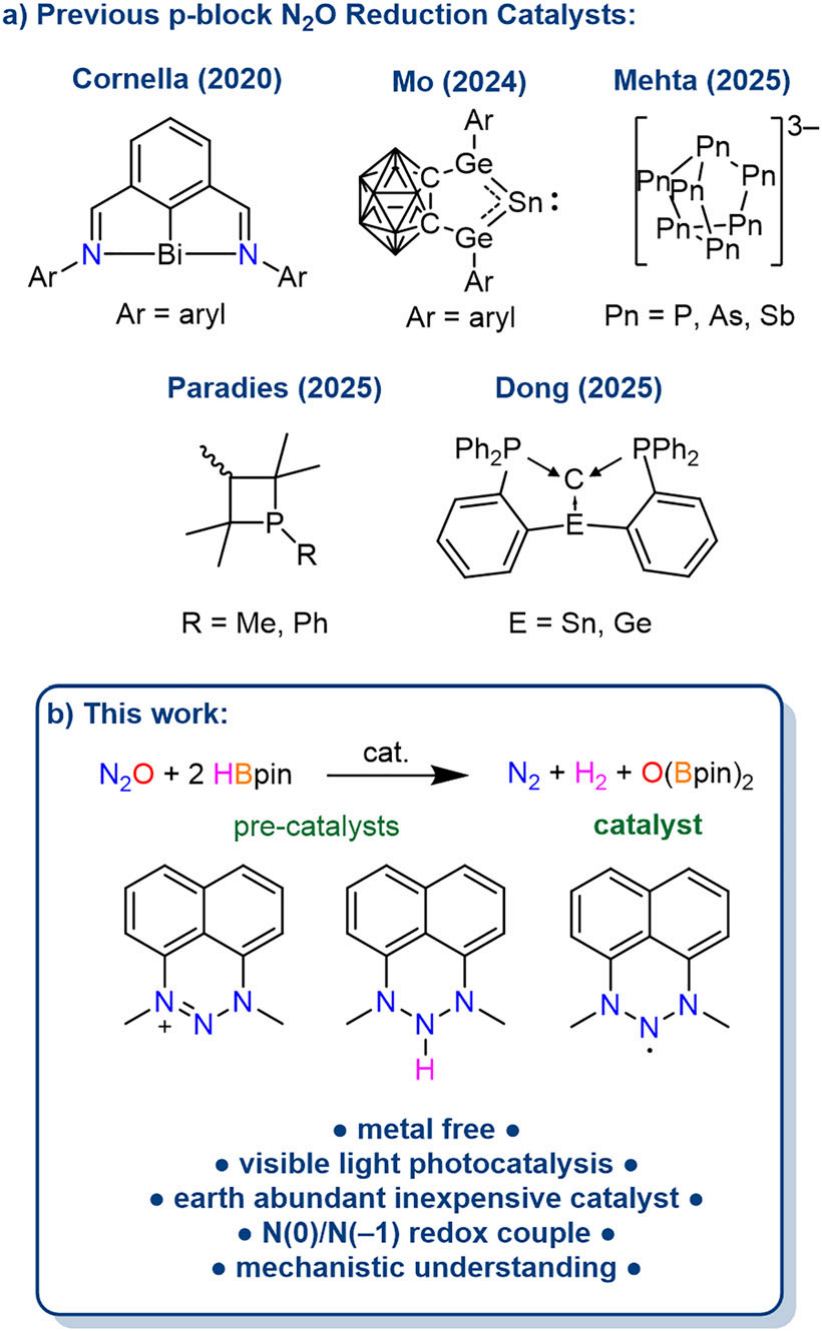
(a) Previous main group systems used for catalytic nitrous oxide reduction. (b) This work.

N‐heterocyclic nitrenium cations have seen a surge of interest in the last decade due to their ease of synthesis, high stability, and potential for photochemical reactivity. An early application of these cations in transition metal‐free catalysis was reported by Mehta and Goicoechea, where through Lewis acid catalysis they could deoxygenate ketones, transfer hydrogenate alkenes, and promote dehydrocoupling reactions [24]. Later, Gandelman and coworkers modified the structure of this nitrenium cation to promote the hydrosilylative reduction of imines [25]. Beyond Lewis acid catalysis, N‐heterocyclic nitrenium cations have also been shown to generate charge‐transfer complexes, which upon irradiation can access one‐electron redox chemistry. These systems have enabled the photocatalytic reduction of aryl halides to facilitate ring closure, photohomolysis of CF_3_CO_2_Cl, reduction of acyl fluorides, and reduction of Se─Se bonds [26, 27, 28, 29]. However, it ought to be noted that the mechanisms for these photocatalytic protocols are not well understood.

Herein, we report the first example of N_2_O reduction to N_2_ mediated by a nitrogen‐centred catalyst (Figure 1b). This finding also represents the first example of a metal‐free homogeneous photocatalytic reduction of N_2_O. Both simple N‐heterocyclic nitrenium salts and the corresponding amine were found to be pre‐catalysts in this transformation, with a nitrogen‐centred radical identified as the catalytically active species. Experimental and computational studies show that visible light irradiation is necessary for this transformation, both in the formation of the active catalyst and in the activation of key catalytic intermediates. To the best of our knowledge, this work represents an unprecedented N(0)/N(–1) redox cycle, and thus significantly advances organo‐main‐group redox chemistry.

## 2 Results and Discussion

First, the reduction of N_2_O (~1 atm) with pinacol borane (HBpin) using 10 mol% nitrenium cation [**1**][BArF] ([BArF] = tetrakis(pentafluorophenyl)borate) was probed (Table 1). The reaction was conducted in N,N‐dimethylformamide (DMF) under 390 nm irradiation for 18 h, after which analysis of the reaction mixture by ^1^H and ^11^B{^1^H} nuclear magnetic resonance (NMR) spectroscopy revealed complete consumption of HBpin, with >99% conversion to O(Bpin)_2_, and detection of H_2_ gas. O(Bpin)_2_ and H_2_ are the expected products from deoxygenation of N_2_O and elimination of N_2_ gas. A range of solvents, reductants, catalyst loadings, and wavelengths of light were screened but showed no further improvement on catalytic performance (Section S2.1). The pressure of N_2_O used in the reaction was also shown to have no effect on turnover, hence ∼1 atm was used throughout these studies. Next, the nitrenium cation with an iodide anion, [**1**][I], and trifluoromethanesulfonate anion ([OTf]^–^), [**1**][OTf], were tested in a similar fashion. Unexpectedly, both gave higher catalytic turnover frequencies (TOFs) despite the more coordinating anions partially quenching the Lewis acidity of the nitrenium cation [24, 30]. Using [**1**][OTf] as the catalyst, the reaction progress was tracked by ^1^H NMR spectroscopy revealing very slow conversion at the start of the reaction, consistent with an induction period (Table S6).

**TABLE 1:** Screening of nitrogen‐based catalysts for the reduction of N_2_O with HBpin.

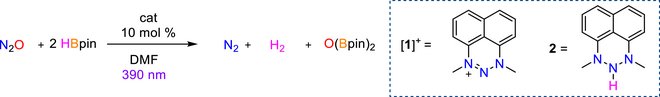					
Catalyst	Loading	Time (h)	Conversion (%)^a^	TON	TOF (h^−1^)
[1][BArF]	10	18	>99	10	0.56
[1][I]	10	14	>99	10	0.71
[1][OTf]	10	8	>99	10	1.25
2	10	2	>99	10	5
2	1	2	18	18	9
2	1	18	68	68	3.8
pyrrole	10	NA	<5^b^	0	0
2,2,6,6‐tetramethylpiperidine	10	NA	<5^b^	0	0

^a^ Determined by ratio of O(Bpin)_2_ to HBpin in ^1^H NMR spectrum.

^b^ Trace conversion due to moisture in N_2_O cylinder.

To rationalise these observations, a mechanism involving an initial hydride transfer from HBpin to [**1**]^+^_,_ generating amine **2** and a pinacol borane cation ([Bpin]^+^) could be envisioned. This type of hydride transfer has previously been reported between nitrenium salts and hydrosilanes [25]. The stability of [Bpin]^+^ would be increased significantly with a more coordinating anion present, consistent with the improved catalytic performance of [**1**][OTf] and [**1**][I] over [**1**][BArF]. However, a stoichiometric reaction of [**1**]^+^ with HBpin under reaction conditions did not lead to formation of **2**, suggesting that a simple hydride transfer was not taking place. Instead, the neutral amine **2** was independently synthesised via literature methods and was employed as a catalyst under the same conditions [25]. Analysis by ^1^H and ^11^B{^1^H} NMR spectroscopy revealed that **2** catalyses the reaction approximately 9× faster than [**1**][BArF] as a (pre‐)catalyst in this reaction. Whilst a simple hydride transfer from HBpin to [**1**]^+^ to form **2** may not be occurring, a cooperative activation of HBpin by [**1**]**^+^** and N_2_O is plausible given recent reports of substrate‐assisted hydride transfer from silanes to nitrenium salts [31]. Although not observed by NMR spectroscopy, ultraviolet–visible (UV–vis) spectroscopy studies show a bathochromic shift when [**1**][BArF] was mixed with HBpin in a 1:1 ratio, consistent with a weak interaction between the two species (Figure 2a). An additional bathochromic shift was observed when the cuvette mixture was pressurised with N_2_O, indicating that N_2_O also interacts with the pair. Independently synthesised compound **2** was also studied by UV–vis spectroscopy and showed an almost identical absorbance spectrum to that of [**1**][OTf] + HBpin + N_2_O, consistent with **2** being formed under the reaction conditions. When [**1**][OTf] was analysed by UV–vis spectroscopy with only N_2_O, no change in the resulting spectrum was observed (Figure S5). Given the poor basicity of N_2_O, this hydride transfer step is expected to be slow, rationalising the relatively poor performance of [**1**]^+^ compared to **2**, and the observed induction period in the reaction profile when [**1**][OTf] was studied.

**FIGURE 2:**
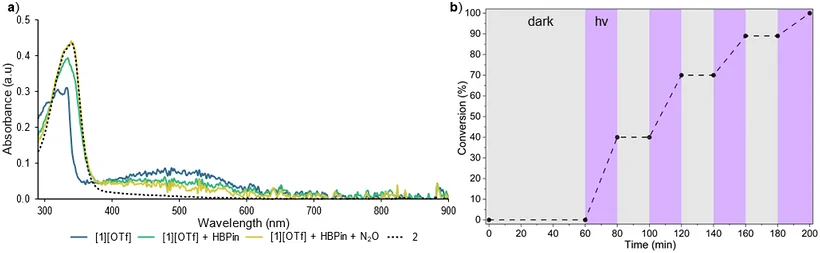
(a) Experimental UV–vis spectra in DMF of [**1**][OTf] (blue), [**1**][OTf] + HBpin (green), [**1**][OTf] + HBpin + N_2_O (yellow), and **2** (black dots). (b) Graph tracking conversion of O(Bpin)_2_, determined by ^1^H NMR spectroscopy, after an initial 1 h dark period followed by alternating 20 min periods in the dark and under 390 nm irradiation.

The reaction was subsequently re‐optimised using **2** as the catalyst; the same conditions were found to be optimal (i.e., 10 mol% catalyst loading, DMF solvent, 390 nm irradiation, 2 h; Section S2.2). Without the need for hydride transfer, H_2_ and H_2_ surrogates (i.e., 1,4‐cyclohexadiene and 9,10‐dihydroanthracene) were investigated as the reductant but showed no conversion (Table S10). B_2_pin_2_ was also investigated but showed poor conversion compared to HBpin. Both blue (456 nm) and green (525 nm) light irradiation were also screened but showed less than 5% conversion. The UV–vis spectrum of **2** shows a strong absorption peak at 350 nm that tails into the 390 nm region (Figure 2a), in line with the need for purple (390 nm) irradiation in these reactions. A range of solvents were also screened and revealed that the reaction proceeded more rapidly in polar solvents. Whilst **2** exhibited slightly higher TOFs in tetrahydrofuran and acetonitrile rather than in benzene, DMF was still found to be the most suitable solvent for the reaction. Reducing the catalyst loading to 5 mol%, and then to 1 mol% of **2**, significantly reduced the conversion of O(Bpin)_2_ to 43% and 18%, respectively. To understand the impact of trace water on the reaction, the reaction was repeated with N_2_O that had been further dried over molecular sieves, and no change in catalytic performance was observed. A thermometer was placed under the reaction conditions to determine the temperature increase caused by 390 nm irradiation process and detected a maximum temperature of 44°C over 2 h. The N_2_O reduction reaction was repeated with **2** as the catalyst using labelled ^15^N_2_O and the formation of ^15^N_2_ was confirmed by ^15^N NMR spectroscopy (Section S2.3.1).

To probe the reaction mechanism, a series of control reactions and stoichiometric reactions were undertaken (Sections S2.1.4 and S2.2.3). First, the N_2_O reduction reaction was repeated with **2** as the catalyst in the absence of light and no conversion of HBpin to O(Bpin)_2_ was observed. Next, the reaction was repeated at 50°C with no 390 nm irradiation, and no conversion of HBpin to O(Bpin)_2_ was observed. These studies are consistent with irradiation with 390 nm light being necessary for the transformation. Next, when the reaction was conducted with alternating 20‐min periods of irradiation and darkness, no reaction progress was observed during the dark periods (Figure 2b). However, the reaction reached completion in less cumulative time under irradiation (80 vs. 120 min), presumably as intermediate steps which do not require light can still proceed in the dark. Unlike the tracked reaction using [**1**][OTf] as the catalyst, no induction period was observed when catalyst **2** was employed with the rate of conversion decreasing as the reaction progresses. Simple cyclic amines, namely pyrrole and 2,2,6,6‐tetramethylpiperidine, were also tested as catalysts at 10 mol% loading in a similar fashion and did not show any catalytic competency (Table 1). Further, irradiation of HBpin and N_2_O in the absence of catalyst in DMF or acetonitrile solvent did not give any conversion. When **2** was irradiated with 390 nm light in CD_3_CN solvent for one hour, the ^1^H NMR spectroscopy resonances corresponding to **2** completely disappeared and gas release could be observed throughout the reaction, presumed to be H_2_ gas. Initially, H_2_ could not be observed in the ^1^H NMR spectrum due to the paramagnetic nature of the sample but leaving the sample for three days and allowing the paramagnetic material to decompose led to observation of a ^1^H NMR spectroscopy resonance in line with H_2_ formation (Section S3.1). The reaction was repeated and analysed by electron paramagnetic resonance (EPR) spectroscopy, which confirmed the formation of **3** ^•^, in accordance with literature reports and presumably formed from homolytic cleavage of the N─H bond (Figure 3) [32]. Formation of H_2_ is in line with homocoupling of two H^•^ radicals [33]. Homolytic cleavage of related N─H bonds is known to occur by strong oxidising agents or ultraviolet light, but has not previously been reported under visible light irradiation.

**FIGURE 3:**
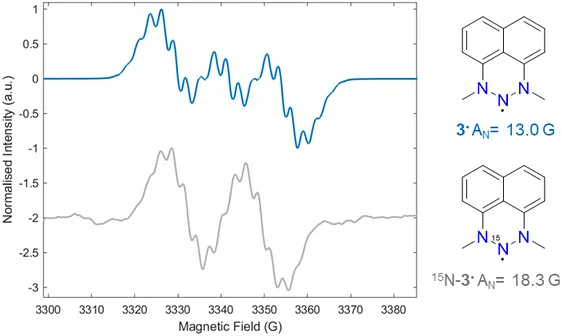
EPR spectra of **3** ^•^ (blue) and ^15^N‐**3** ^•^ (grey) with the molecular structures and anisotropic coupling to the central nitrogen shown. Compound **3** ^•^ and its EPR spectrum have previously been reported in the literature [32].

Based on experimental observations, a plausible mechanism was proposed and refined using density functional theory (DFT) at the ωB97x‐D4/def2‐TZVP (SMD = DMF) level of theory (Figure 4). Time‐dependent DFT (TDDFT) studies were conducted at the revPBE/def2‐TZVP (SMD = DMF) level of theory after screening a range of functionals to best reproduce the experimental UV–vis spectrum of **2** (Section S4.1.2). Further computational details and a full justification for each step can be found in Section S4. The first proposed step in the mechanism is a photo‐induced homolytic cleavage of the N─H bond in **2** to form the active catalyst **3^•^**, consistent with the UV–vis and EPR data. TDDFT and complete active space self‐consistent field (CASSCF) calculations show that an excitation is accessible under the employed 390 nm irradiation (TDDFT excitation wavelength = 352.6 nm; CASSCF excitation wavelength = 368.2 nm; Section S4.2). DFT calculations suggest that this N─H bond cannot cleave spontaneously upon excitation to the first singlet state. However, a small singlet‐triplet gap suggests that intersystem crossing in this case is feasible. Here, homolytic cleavage of the N─H bond (**TS1**) is energetically feasible, with the transition state having a ∼8 kcal mol^−1^ barrier. A lower energy transition state was also located where one triplet‐state molecule of **2** collides with a singlet‐state molecule of **2** to lose H_2_ with a barrier of 6.3 kcal mol^−1^; however, this was deemed to be chemically less probable despite its lower barrier.

**FIGURE 4:**
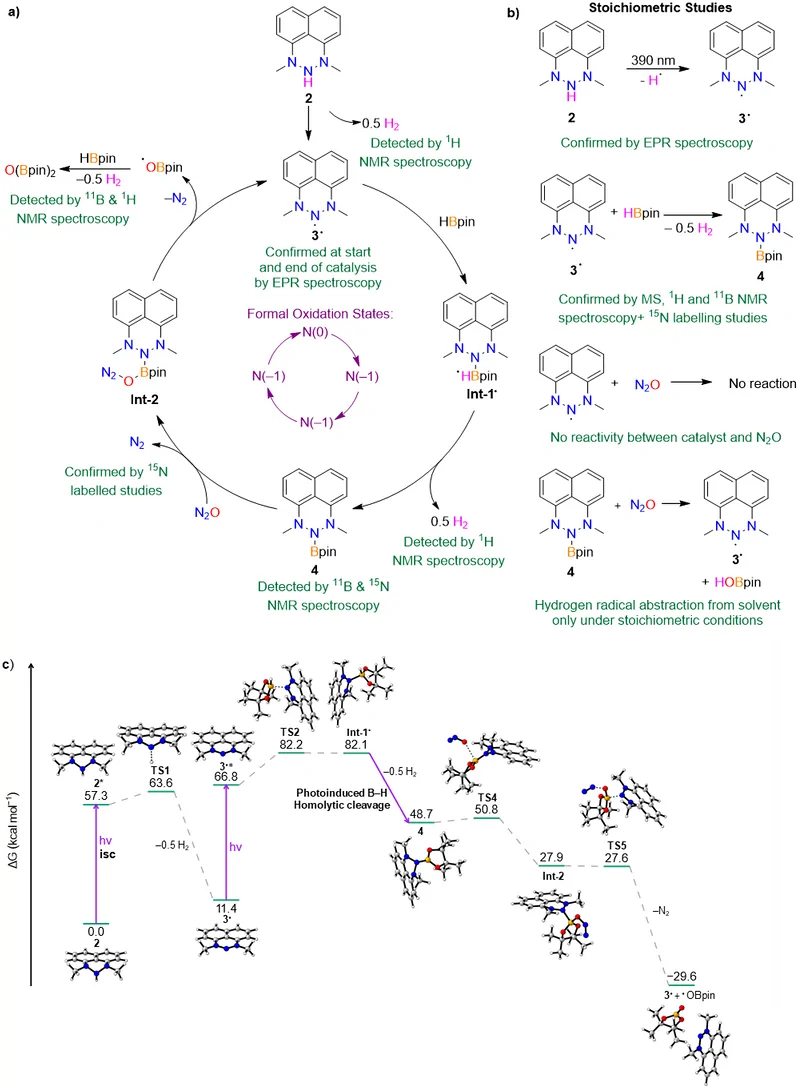
(a) Proposed catalytic cycle for the reduction of N_2_O with **2** as the pre‐catalyst. (b) Stoichiometric reactions relevant to the mechanism presented on the right. (c) Calculated reaction pathway for the reduction of N_2_O with HBpin using **2** as a pre‐catalyst. Gibbs energies in kcal mol^−1^ (ωB97X‐D4/def2‐TZVP with SMD = DMF solvation).

Next, we investigated whether **3** ^•^ preferentially reacts with N_2_O or HBpin (Figure 4b). ^15^N‐**2** was synthesised and reacted independently with HBpin and N_2_O under 390 nm irradiation (Section S3.2). In the case of N_2_O, no ^15^N NMR spectroscopy resonances could be observed, and EPR spectroscopy studies revealed that ^15^N‐**3** ^•^ was still present in solution (with coupling constants in line with literature values; Figure 3) [32]. However, when reacted with HBpin, a new resonance in the ^15^N NMR spectrum was observed at 50 ppm as well as a new resonance at 25 ppm in the ^11^B{^1^H} NMR spectrum. In more dilute samples, a doublet splitting pattern can be observed for the resonance at 25 ppm in the ^11^B{^1^H} NMR spectrum, in line with splitting from the ^15^N atom (Figure S30). Further, EPR spectroscopy confirmed the absence of any detectable paramagnetic species in solution. Therefore, it was proposed that **3** ^•^ first reacts with HBpin to form **4** and generate another hydrogen radical (Figure 4a). Mass spectrometry studies on the crude reaction mixture confirmed the presence of a species with a *m*/*z* value consistent with ^15^N‐**4** (Section S3.2.2). It is also noteworthy that reaction of **3** ^•^ with HBpin in the absence of irradiation shows no conversion to **4**. Further, during the catalytic studies with **2**, when reactions were tracked with ^11^B{^1^H} NMR spectroscopy, the formation and consumption of an intermediate with a resonance at 25 ppm was detected, in line with **4** being a relevant catalytic intermediate. Attempts were made to independently synthesise and isolate **4** with no success. When formation of **4** was probed using DFT, two transition states and an intermediate were located, indicating a stepwise process (Figure 4c; Section S4.3.3). First, a photoexcited molecule of **3** ^•^ reacts with HBpin to form a tetra‐coordinate boron intermediate (**Int‐1** ^•^) with a barrier of 15.4 kcal mol^−1^. This intermediate then eliminates a hydrogen radical to form **4**. TDDFT studies indicate that upon excitation of **Int‐1** ^•^, this hydrogen radical elimination is spontaneous with a negligible energetic barrier, however the computational method used for studying this step makes the calculated energetic values incomparable with the rest of the mechanism (see Section S4.3.3 for further details). The stoichiometric reaction of **3** ^•^ with HBpin was slower than the other steps (*ca* 3 h), consistent with this step corresponding to the highest computed barrier in the catalytic cycle. Further, our inability to observe **Int‐1** ^•^ by NMR spectroscopy supports this having a very small barrier.

The next step in the proposed cycle is the coordination of N_2_O to **4**, forming a new intermediate (**Int‐2**). A transition state was found for this step where the oxygen of N_2_O coordinates to the boron of **4** in the triplet state with a barrier of 2.1 kcal mol^−1^ (Section S4.4)_._ This is followed by a homolytic cleavage of the N─O bond of N_2_O and the N─B bond of **4** (**TS5**), reforming the catalyst **3** ^•^ and a molecule of ^•^OBpin. Here, the electronic energy of the transition state is slightly higher than that of **Int‐2** by 0.02 kcal mol^−1^_,_ enabling the transition state to be located, but the Gibbs free energy is lower (Figure S45). Independent in situ synthesis of ^15^N‐**4** and subsequent reactivity with N_2_O yielded no reaction under ambient conditions, in line with the need for **4** to be photoexcited into the triplet state for the reaction to occur. However, upon irradiation with 390 nm light for 1 h, complete conversion to HOBpin was observed in the ^1^H and ^11^B{^1^H} NMR spectra (Section S3.3). We were unable to detect **Int‐2** experimentally, likely due to the very low barrier to its homolytic cleavage. As there is no additional HBpin present in this reaction mixture, we postulate that ^•^OBpin abstracts a hydrogen atom from DMF solvent in the stoichiometric studies. However, under catalytic conditions when there is an excess of HBpin, the ^•^OBpin can directly react with HBpin, resulting in the formation of O(Bpin)_2_ and a hydrogen radical which can homocouple to form H_2_ (Section S4.4.3). Hence, when the catalytic reaction was undertaken in DMF‐d_7_, with no other deuterium sources present, only H_2_ formation was detected, not HD. This suggests the preferential hydrogen atom abstraction from HBpin and negligible deuterium atom abstraction from DMF‐d_7_ under catalytic conditions (Section S2.3.3.1). Further, repeating the catalytic reaction with DBpin as the only deuterium source led to formation of D_2_, again supporting the hypothesis that the preferred hydrogen atom abstraction site is from another molecule of HBpin rather than DMF solvent (Section S2.3.2). However, repeating the reaction with stoichiometric amounts of **2** and HBpin in DMF‐d_7_ did afford small HD signals (approximately 10% compared to H_2_) in the ^1^H NMR spectrum, demonstrating that at high ^•^OBpin concentrations or when minimal amounts of HBpin are present, DMF(‐d_7_) can also act as a hydrogen atom source (Section S2.3.3.2). Finally, NMR and EPR spectroscopy studies on the catalytic reaction mixture, both during and after the reaction reached complete conversion, confirmed the presence of **3** ^•^ rather than the cation [**1**]^+^ or the amine **2**, supporting assignment of **3** ^•^ as the active catalyst. Thus, considering formal oxidation states this reaction can be thought to operate via a previously unprecedented N(0)/N(–1) redox cycle at the central nitrogen atom.

To probe the robustness of the catalyst, 10 mol% of **2** was pressurised with N_2_O in DMF solvent and irradiated for 1 h under 390 nm light. HBpin was then added and irradiated for a further 2 h, which led to clean formation of the expected products, albeit only at 80% conversion (Section S2.5.1). Next, 10 mol% of **2** was mixed with HBpin in DMF solvent and irradiated with 390 nm light for 1 h, then the reaction mixture was pressurised with N_2_O and irradiated for a further 2 h. This again led to clean formation of O(Bpin)_2_ and confirmed that the catalyst is resilient to an excess of either substrate under irradiation (Section S2.5.2).

## 3 Conclusion

In this work, we present the first metal‐free homogeneous photocatalytic protocol for N_2_O reduction to benign N_2_ gas. Experimental and computational mechanistic investigations reveal that the nitrogen‐based photocatalyst employed accesses a previously unknown N(0)/N(–1) redox cycle, and highlight the role that visible‐light irradiation plays in the mechanism. This work expands the scope of p‐block catalysts for N_2_O reduction and advances our fundamental understanding of nitrogen redox catalysis and photochemistry.

## Author Contributions

**Reece Lister‐Roberts**: investigation, writing – original draft, methodology, writing – review and editing, formal analysis, data curation. **Agamemnon E. Crumpton**: investigation, writing – review and editing, methodology, formal analysis, data curation. **Meera Mehta**: conceptualization, funding acquisition, writing – review and editing, formal analysis, project administration, supervision, resources.

## Conflicts of Interest

The authors declare no conflicts of interest.

## Supporting information




**Supporting File 1**: anie74367‐sup‐0001‐SuppMat.pdf.

## Data Availability

The data that supports the findings of this study are available in the Supporting Information of this article.

## References

[1] A. R. Ravishankara , J. S. Daniel , and R. W. Portmann , “Nitrous Oxide (N_2_O): The Dominant Ozone‐Depleting Substance Emitted in the 21st Century,” Science 326 (2009): 123–125, 10.1126/science.1176985. 19713491

[2] C. Sánchez‐Sánchez , R. Muñoz , E. Alfonso‐González , et al., “Hydrogen‐Induced Reduction Improves the Photoelectrocatalytic Performance of Titania,” ACS Applied Energy Materials 7 (2024): 2101–2108, 10.1021/acsaem.3c02707. PMC10966639 38550299

[3] A. Genoux and K. Severin , “Nitrous Oxide as Diazo Transfer Reagent,” Chemical Science 15 (2024): 13605–13617, 10.1039/D4SC04530K. PMC11323477 39156938

[4] N. Lehnert , H. T. Dong , J. B. Harland , A. P. Hunt , and C. J. White , “Reversing Nitrogen Fixation,” Nature Reviews Chemistry 2 (2018): 278–289, 10.1038/s41570-018-0041-7.

[5] T. M. Hood , R. S. C. Charman , D. J. Liptrot , and A. B. Chaplin , “Copper(I) Catalysed Diboron(4) Reduction of Nitrous Oxide,” Angewandte Chemie International Edition 63 (2024): e202411692, 10.1002/anie.202411692. 39011672

[6] X. Chen , H. Wang , S. Du , M. Driess , and Z. Mo , “Deoxygenation of Nitrous Oxide and Nitro Compounds Using Bis(N‐Heterocyclic Silylene)Amido Iron Complexes as Catalysts,” Angewandte Chemie International Edition 61 (2022): e202114598, 10.1002/anie.202114598. 34766416

[7] P. Jurt , A. S. Abels , J. J. Gamboa‐Carballo , et al., “Reduction of Nitrogen Oxides by Hydrogen With Rhodium(I)–Platinum(II) Olefin Complexes as Catalysts,” Angewandte Chemie International Edition 60 (2021): 25372–25380, 10.1002/anie.202109642. PMC9298341 34510678

[8] I. Ortega‐Lepe , P. Sánchez , L. L. Santos , et al., “Catalytic Nitrous Oxide Reduction With H_2_ Mediated by Pincer Ir Complexes,” Inorganic Chemistry 61 (2022): 18590–18600, 10.1021/acs.inorgchem.2c02963. PMC10441893 36346983

[9] R. Zeng , M. Feller , Y. Ben‐David , and D. Milstein , “Hydrogenation and Hydrosilylation of Nitrous Oxide Homogeneously Catalyzed by a Metal Complex,” Journal of the American Chemical Society 139 (2017): 5720–5723, 10.1021/jacs.7b02124. PMC5408329 28402635

[10] F. Le Vaillant , A. Mateos Calbet , S. González‐Pelayo , et al., “Catalytic Synthesis of Phenols With Nitrous Oxide,” Nature 604 (2022): 677–683. 10.1038/s41586-022-04516-4 PMC9046086 35478236

[11] A. Mateos‐Calbet , P. C. Bruzzese , M. A. Mermigki , A. Schnegg , D. A. Pantazis , and J. Cornella , “Rapid Oxygen Atom Transfer at a Catalysis‐Relevant Ni(I)–Alkyl Complex With N_2_O,” Journal of the American Chemical Society 147 (2025): 19438–19443, 10.1021/jacs.5c03351. PMC12164269 40442965

[12] A. Hart , “Circular Economy: Closing the Catalyst Loop With Metal Reclamation From Spent Catalysts, Industrial Waste, Waste Shells, and Animal Bones,” Biomass Conversion and Biorefinery 13 (2023): 11483–11498, 10.1007/s13399-021-01942-8.

[13] K. M. P. Wheelhouse , R. L. Webster , and G. L. Beutner , “Advances and Applications in Catalysis With Earth‐Abundant Metals,” Organic Process Research & Development 27 (2023): 1157–1159, 10.1021/acs.oprd.3c00207.

[14] E. Otten , R. C. Neu , and D. W. Stephan , “Complexation of Nitrous Oxide by Frustrated Lewis Pairs,” Journal of the American Chemical Society 131 (2009): 9918–9919, 10.1021/ja904377v. 19569691

[15] Z. Mo , E. L. Kolychev , A. Rit , J. Campos , H. Niu , and S. Aldridge , “Facile Reversibility by Design: Tuning Small Molecule Capture and Activation by Single Component Frustrated Lewis Pairs,” Journal of the American Chemical Society 137 (2015): 12227–12230, 10.1021/jacs.5b08614. 26356306

[16] A. G. Tskhovrebov , E. Solari , M. D. Wodrich , R. Scopelliti , and K. Severin , “Covalent Capture of Nitrous Oxide by N‐Heterocyclic Carbenes,” Angewandte Chemie International Edition 51 (2012): 232–234, 10.1002/anie.201106589. 22095905

[17] A. G. Tskhovrebov , B. Vuichoud , E. Solari , R. Scopelliti , and K. Severin , “Adducts of Nitrous Oxide and N‐Heterocyclic Carbenes: Syntheses, Structures, and Reactivity,” Journal of the American Chemical Society 135 (2013): 9486–9492, 10.1021/ja4030287. 23758062

[18] L. Anthore‐Dalion , E. Nicolas , and T. Cantat , “Catalytic Metal‐Free Deoxygenation of Nitrous Oxide With Disilanes,” ACS Catalysis 9 (2019): 11563–11567, 10.1021/acscatal.9b04434.

[19] Y. Pang , M. Leutzsch , N. Nöthling , and J. Cornella , “Catalytic Activation of N_2_O at a Low‐Valent Bismuth Redox Platform,” Journal of the American Chemical Society 142 (2020): 19473–19479, 10.1021/jacs.0c10092. PMC7677929 33146996

[20] B. Lei , F. Cao , M. Chen , X. Wang , and Z. Mo , “Bisgermylene‐Stabilized Stannylone: Catalytic Reduction of Nitrous Oxide and Nitro Compounds via Element‐Ligand Cooperativity,” Journal of the American Chemical Society 146 (2024): 17817–17826, 10.1021/jacs.4c03227. 38780163

[21] B. van IJzendoorn , R. Lister‐Roberts , N. Kaltsoyannis , and M. Mehta , “Catalytic Nitrous Oxide Degradation With Group 15 Clusters,” Journal of the American Chemical Society 147 (2025): 30317–30325. 10.1021/jacs.5c09618 PMC12371874 40767358

[22] Z. Liu , X. Li , Z. Wang , et al., “E^II^/E^IV^O (E = Ge, Sn) Catalyzed Nitrous Oxide Activation and Chemodivergent Reduction of Nitroarenes,” Angewandte Chemie International Edition 64 (2025): e202515638, 10.1002/anie.202515638. 40995739

[23] R. Zhou , V. Medvarić , T. Werner , and J. Paradies , “Metal‐Free Reduction of Nitrous Oxide via P^III^/P^V^═O Cycling: Mechanistic Insights and Catalytic Performance,” Journal of the American Chemical Society 147 (2025): 37879–37884, 10.1021/jacs.5c06190. 40948462

[24] M. Mehta and J. M. Goicoechea , “Nitrenium Salts in Lewis Acid Catalysis,” Angewandte Chemie International Edition 59 (2020): 2715–2719, 10.1002/anie.201915547. 31808974

[25] K. Singh , I. Avigdori , A. Kaushansky , N. Fridman , D. Toledano , and M. Gandelman , “New Generation of Nitrenium Salts: Catalytic Hydrosilylation of Imines and a Mechanism of Action of Nitrogen Lewis Acids,” ACS Catalysis 12 (2022): 6831–6839, 10.1021/acscatal.2c01297.

[26] K.‐Q. Chen , B.‐B. Zhang , Z.‐X. Wang , and X.‐Y. Chen , “N‑Heterocyclic Nitreniums Can Be Employed as Photoredox Catalysts for the Single‐Electron Reduction of Aryl Halides,” Organic Letters 24 (2022): 4598–4602, 10.1021/acs.orglett.2c01702. 35709368

[27] X.‐J. Ren , P.‐W. Liao , H. Sheng , Z.‐X. Wang , and X.‐Y. Chen , “N‐Heterocyclic Nitrenium‐Catalyzed Photohomolysis of CF_3_SO_2_Cl for Alkene Trifluoromethylation,” Organic Letters 25 (2023): 6189–6194, 10.1021/acs.orglett.3c02380. 37578296

[28] C.‐S. Zhang , K.‐Q. Chen , L. Zhou , Z.‐S. Yang , Z.‐X. Wang , and X.‐Y. Chen , “Photoinduced N‐Heterocyclic Nitrenium‐Catalyzed Single Electron Reduction of Se─Se Bond for the Generation of Nucleophilic Selenolates,” Organic Chemistry Frontiers 11 (2024): 2070–2074, 10.1039/D3QO02012F.

[29] L. Bao , Z.‐X. Wang , and X.‐Y. Chen , “Photoinduced N‐Heterocyclic Nitrenium‐Catalyzed Single Electron Reduction of Acyl Fluorides for Phenanthridine Synthesis,” Organic Letters 25 (2023): 565–568, 10.1021/acs.orglett.3c00049. 36637257

[30] R. R. Golwankar , T. D. I. Curry , C. J. Paranjothi , and J. D. Blakemore , “Molecular Influences on the Quantification of Lewis Acidity With Phosphine Oxide Probes,” Inorganic Chemistry 62 (2023): 9765–9780, 10.1021/acs.inorgchem.3c00084. 36943934

[31] D. Ranolia , A. Koronatov , G. Israeli , A. Kaushansky , N. Fridman , and M. Gandelman , “Organocatalytic Deoxygenative Reduction of α,β‐Unsaturated Ketones,” Journal of the American Chemical Society 148 (2026): 6238–6248, 10.1021/jacs.5c18403. 41660972

[32] A. Koronatov , A. Mauda , B. Tumansky , et al., “Multimodal Reactivity of N─H Bonds in Triazanes and Isolation of a Triazinyl Radical,” Journal of the American Chemical Society 144 (2022): 23642–23648, 10.1021/jacs.2c11113. 36525645

[33] G. Dixon‐Lewis , M. M. Sutton , and A. Williams , “The Kinetics of Hydrogen Atom Recombination,” Discussions of the Faraday Society 33 (1962): 205, 10.1039/df9623300205.

