# Supplementary material for: Nitrogen Radical Photocatalysed Reduction of Nitrous Oxide
Source: Angew Chem Int Ed Engl. 2026 Aug 23;65(42):e9521232. doi: 10.1002/anie.9521232 (PMC13653572; doi:10.1002/anie.9521232)
Supplement: Supplementary file 1 [file ANIE-65-e9521232-s001.pdf]

Supplementary Information

Nitrogen Radical Photocatalysed Reduction of Nitrous Oxide

Reece Lister-Roberts[1], Agamemnon E. Crumpton[1], Meera Mehta[1]*

*meera.mehta@chem.ox.ac.uk

1. Department of Chemistry, University of Oxford, Oxford, OX1 3TA, UK

Table of Contents

1. Methods and Materials 4
 1.1 Experimental Considerations 4
 1.2 Analytical Considerations 4
 1.3 Computational Considerations 5
2. Experimental Data 7
 2.1 Optimisation, Control Reactions, and Characterisation using $[1]^+$ as the Catalyst 7
  2.1.1 Initial Screening with $[1]^+$ as the Catalyst 7
  2.1.2 Optimised Procedure for the Reduction of N_2O with HBpin using [1][OTf] as the catalyst. 10
  2.1.3 Tracked Reaction with [1][OTf] 11
  2.1.4 Control Reactions and Stoichiometric Reactions with [1][OTf] 12
  2.1.5 UV-Vis studies with [1][OTf] 14
 2.2 Optimisation and Control Reactions for 2 15
  2.2.1 Initial Screening with 2 as the Catalyst 15
  2.2.2 Optimised Procedure for the Reduction of N_2O with HBpin using 2 as the catalyst. 17
  2.2.3 Control Reactions and Stoichiometric Reactions with 2 18
  2.2.4 UV-Vis spectrum of 2 19
 2.3 Labelled Experiments with 2 20
  2.3.1 ^{15}N-N_2O 20
  2.3.2 DBpin 21
  2.3.3 DMF-d_7 22
 2.4 Tracked Interval Reaction 24
 2.5 Catalyst Stability Towards Excess Substrate 26
  2.5.1 Catalyst Stability Towards Excess N_2O 26
  2.5.2 Catalyst Stability Towards Excess HBpin 27
3. Experimental Mechanistic Studies 28
 3.1 Studies on the homolytic cleavage of the N–H bond in 2 28
 3.2 Experimental studies on formation of 4 30
  3.2.1 Stoichiometric reactivity of ^{15}N-2 with DBpin 30
  3.2.2 Stoichiometric reactivity of ^{15}N-2 with HBpin 31
 3.3 Studies on the reaction of 4 with N_2O 34
4. Computational Mechanistic Studies 35
 4.1 Method Benchmarking 35
  4.1.1 Structural Benchmarking 35
  4.1.2 Excited-State Benchmarking 36
 4.2 Photochemical Activation of 2 38
  4.2.1 Exploration of the Singlet Excited-State Manifold 38
  4.2.2 Population of the Triplet Manifold 39

4.2.3 Triplet-State N–H Activation 40
4.3 Reactivity of 3• 41
4.3.1 Doublet-State Reactivity of 3• Towards HBpin 41
4.3.2 Accessible Higher Spin States 42
4.3.3 Formation of Int-1• and 4 43
4.4 N_2O Reduction and Product Formation 45
4.4.1 Activation of N_2O 45
4.4.2 Intersystem Crossing Studies on 4 45
4.4.3 Formation of $O(Bpin)_2$ 46
4.5 CASSCF 47
4.5.1 CASSCF of 2 47
4.5.2 CASSCF of 3• 49
4.5.3 CASSSCF of Int-1• 52
4.5.4 CASSCF of 4 53
4.6 TDDFT Orbitals 55
4.6.1 TDDFT S_0->S_1 NTO transition of 2 55
4.6.2 TDDFT D_0->D_4 Natural Transition Orbitals of 3• 55
4.6.3 TDDFT Q_0->Q_2 Natural Transition Orbitals of Int-1• 55
4.6.4 TDDFT S_0->S_1 Natural Transition Orbitals of 4 55
4. References 56
5. DFT Coordinates 58

1. Methods and Materials

1.1 Experimental Considerations

All manipulations were carried out under an inert atmosphere using standard Schlenk-line and glovebox methodology unless specifically stated. All glassware was flame-dried before use.

Innovative Technologies anhydrous engineering solvent purification system was used to obtain dry tetrahydrofuran (THF), dimethylformamide (DMF), diethyl ether, hexane, dichloromethane (DCM), 1,2-difluorobenzene (*o*DFB), and acetonitrile (CH_3CN). Deuterated chloroform ($CDCl_3$, contains 1% TMS w/w), methanol (MeOD-d_4), DMF (DMF-d_7), dichloromethane (CD_2Cl_2), and acetonitrile (CD_3CN) were dried and stored over 3 Å molecular sieves. The solvents obtained in this way were subsequently degassed.

The following compounds were purchased from commercial suppliers and used without further purification: tert-butyl nitrite (Apollo Scientific), methyl triflate (Sigma-Aldrich), 1,8-diaminonaphthalene (Sigma-Aldrich), acetic acid (Sigma-Aldrich), potassium chloride (Sigma-Aldrich), bromopentafluorobenzene (Sigma-Aldrich), n-butyllithium (1.6 M in hexanes; Sigma-Aldrich), methyl iodide (Sigma-Aldrich), sodium borohydride (Sigma-Aldrich), nitrous oxide (N_2O; CK Isotopes), ^{15}N-N_2O (CK Isotopes), ^{15}N-tert-butyl nitrite (Sigma-Aldrich), pinacol borane (HBpin; Sigma-Aldrich), bis(pinacolato)diboron (B_2pin_2; Sigma-Aldrich), dihydroanthracene (Sigma-Aldrich), cyclohexadiene (Sigma-Aldrich), triethylsilane (Et_3SiH; Sigma-Aldrich), triphenylsilane (Ph_3SiH; Sigma-Aldrich), dimethylamine borane ($HNMe_2$-BH_3; Sigma-Aldrich).

The syntheses of potassium tetrakis(pentafluorophenyl)borate ([BArF]), (^{15}N-)[**1**][OTf], [**1**][BArF], [**1**][I], (^{15}N-)**2,** and DBpin (1.4×10^{-4} mmol / µL in C_6D_6) were carried out according to literature procedures.[1–5]

Photochemical experiments were conducted using Kessil PR160L LEDs (390 nm, 55 W max; 456nm, 55W max; 525 nm, 45 W max) at 100% intensity. Reactions were conducted inside a metal box with fan cooling, reaching a maximum temperature of 44 °C after 2 hours.

1.2 Analytical Considerations

NMR spectroscopy. ^{1}H, ^{2}H ^{11}B{^{1}H}, ^{11}B NMR spectra were recorded on a Bruker AVIII 400 spectrometer (operating frequencies: 399.78 MHz, 61.41 MHz, 128.6 MHz, and 128.6 MHz, respectively) or a Bruker AVIII 500 spectrometer (operating frequency: 499.94 MHz, 160.40 MHz, and 160.40 MHz). ^{15}N NMR spectra were recorded on a Bruker AVIII 500 spectrometer at 50.67 MHz. ^{1}H chemical shifts were internally referenced to the residual solvent resonances. NMR samples were prepared under an inert nitrogen atmosphere in a 5 mm J Young NMR tube. All NMR data were analysed using MestReNova V15.1.0.

Mass spectrometry. Mass spectrometry samples were analysed by the mass spectrometry service at the University of Oxford using an electrospray ionisation (ESI) equipped Waters RDa bench-top time of flight mass spectrometer. Samples were prepared under a nitrogen atmosphere and directly injected into the ionisation source of the mass spectrometer.

Ultraviolet–visible spectroscopy. Ultraviolet-visible (UV-Vis) electronic absorption spectra were recorded on a Mettler Toledo UV5Bio spectrophotometer using 10 mm path length quartz J Young cuvettes.

Electron paramagnetic resonance. Electron paramagnetic resonance (EPR) spectra were recorded at X-band (9.4 – 9.8 GHz) with a Bruker EMXmicro spectrometer at 298 K. All EPR spectra were plotted using MATLAB R2024a.

1.3 Computational Considerations

All calculations were performed using ORCA 6.1.1.[6–8]

Ground-state geometry optimisations were carried out using the ωB97X-D4 density functional in conjunction with the def2-TZVP basis set.[9–12] Solvent effects were included using the solvation model based on density (SMD) implicit solvation model for DMF.[13] Harmonic vibrational frequency calculations were subsequently performed at the same level of theory to verify the nature of all stationary points and to obtain thermal corrections to the Gibbs free energies. Minima were characterised by the absence of imaginary frequencies, whereas transition states possessed a single imaginary frequency corresponding to the reaction coordinate.

Excited-state calculations were performed using time-dependent density functional theory (TDDFT) with the def2-TZVP basis set and the SMD(DMF) solvation model. Following benchmarking against the experimental UV–visible absorption spectrum of **2**, the revPBE density functional was employed for excited-state calculations, excited-state geometry optimisations, and spin-state investigations.[14,15] Preliminary exploration of excited-state potential energy surfaces was performed using B97-3c in conjunction with the SMD(DMF) solvation model to enable efficient screening of multiple reaction pathways.[16]

Multireference calculations were carried out using state-averaged Complete Active Space Self-Consistent Field (CASSCF) followed by second-order perturbative CASPT2 corrections employing the def2-TZVP basis set.[17–20] For **2**, an active space of (8,8) was employed and averaged over ten electronic roots. For **3$^{\bullet}$**, an active space of (9,9) was employed and averaged over ten electronic roots.

Unless otherwise stated, all reported energies correspond to Gibbs free energies obtained at 298.15 K. Electronic energies are explicitly identified where discussed.

Figures were prepared using ChimeraX and CYLview20.[21,22]

2. Experimental Data

2.1 Optimisation, Control Reactions, and Characterisation using [1]⁺ as the Catalyst

$$N_2O + 2\ HBpin \xrightarrow[\text{10 mol \%}]{\text{cat}} N_2 + H_2 + O(Bpin)_2$$

2.1.1 Initial Screening with [1]⁺ as the Catalyst

To a J Young NMR tube, a solution of [**1**]$^+$ (0.0057 mmol, 10 mol%) in solvent (0.5 mL) and reductant (0.057 mmol, 1.0 equiv.) was added. The reaction mixture was immediately frozen at 77 K. While frozen, the headspace of the NMR tube was evacuated. N_2O was then condensed into the NMR tube resulting in approximately 1 atm pressure at room temperature. The NMR tube was removed from the liquid nitrogen and after thawing, the reaction mixture was allowed to react for 18 hours under visible light irradiation. The reaction was monitored by ^{1}H and ^{11}B{^{1}H} NMR spectroscopy. NMR conversions were determined by the ratio HBpin/product from the integrations of the crude ^{1}H and ^{11}B{^{1}H} NMR spectra. Note: Due to trace amounts of water in the N_2O cylinder, a small HOBpin resonance (<5 %) can be seen in control reactions. For this reason, any conversion <5 % is considered to be from this trace water and listed as <5 %.

Table S1: Solvent screening for reduction of N_2O with HBpin with [**1**][BArF] as the catalyst after 18 h under 390 nm irradiation.

**Solvent**	**% Conversion (^{1}H NMR spectroscopy)**	**% Conversion (^{11}B{^{1}H} NMR spectroscopy)**
CD_2Cl_2	<5	<5
*o*DFB	<5	<5
DMF	>99	>99
THF	18	16
CD_3CN	27	25

Table S2: Reductant screen (0.057 mmol) for reduction of N_2O with [**1**][BArF] as the catalyst after 18 h in DMF under 390 nm irradiation.

Reductant	% Conversion (^{1}H NMR spectroscopy)
HBpin	99
$HSiEt_3$	<5
$HSiPh_3$	<5
Cyclohexadiene	<5
9,10-dihydroanthracene	<5
H_2*	<5
$HNMe_2$-BH_3	<5
B_2pin_2	<5

* An excess of gas was used, ~1 atm.

Table S3: Irradiation wavelength screening for reduction of N_2O with [**1**][BArF] as the catalyst after 18 h in DMF.

Wavelength (nm)	Catalyst Loading (mol %)	% Conversion (^{1}H NMR spectroscopy)
390	10	>99
	5	68*
	1	19
456	10	22
	5	11
	1	6
525	10	15
	5	8
	1	6

* = Conversion determined by ^{11}B{^{1}H} NMR spectroscopy

Table S4: Catalyst screening for reduction of N_2O with HBpin in DMF under 390 nm irradiation.

[**1**]$^+$ [Anion]	Time (h)	% Conversion (^{1}H NMR spectroscopy)	% Conversion (^{11}B{^{1}H} NMR spectroscopy)
[BArF]$^–$	8	11	8
	18	>99	>99
[OTf]$^–$	8	>99	>99
[I]$^–$	8	54	50
	14	>99	>99

In order to study the effect of pressure on the reaction turnover, a modified version of the general procedure was undertaken.

To a J Young NMR tube, a solution of [**1**][OTf] (0.0029 mmol, 5 mol%) in DMF (0.5 mL) and HBpin (0.057 mmol, 1.0 equiv.) was added. The reaction mixture was immediately frozen at 77 K. While frozen, the headspace of the NMR tube was evacuated. The NMR tube was then removed from the liquid nitrogen and the solution was thawed. The tube was then charged with N_2O at the pressure identified in Table S5. The reaction was allowed to react for 8 hours under 390 nm light irradiation. The reaction was monitored by ^{1}H and ^{11}B{^{1}H} NMR spectroscopy. NMR conversions were determined by the ratio HBpin/product from the integrations of the crude ^{1}H and ^{11}B{^{1}H} NMR spectra. Note: Due to trace amounts of water in the N_2O cylinder, a small HOBpin resonance (<5 %) can be seen in control reactions. For this reason, any conversion < 5 % is considered to be from this trace water and listed as <5 %.

Table S5: Pressure screening for the reduction of N_2O with [**1**][OTf] (5 mol%) as the catalyst after 8 h in DMF under 390 nm irradiation.

Pressure (atm)	% Conversion (^{1}H NMR spectroscopy)
0.5	51
1	48
1.5	49
2	50

2.1.2 Optimised Procedure for the Reduction of N_2O with HBpin using [1][OTf] as the catalyst.

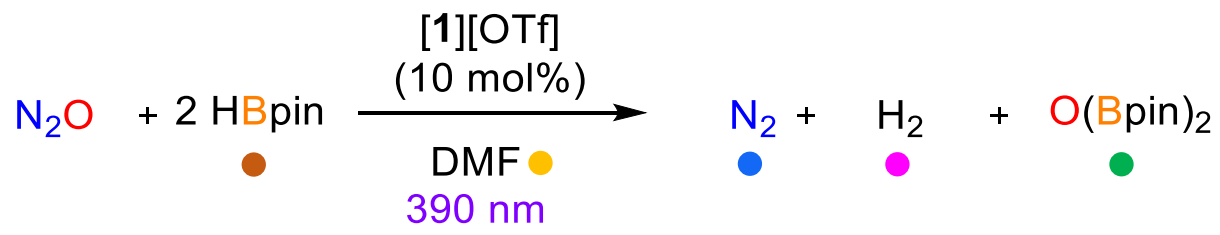

The optimised procedure for the reduction of N_2O with HBpin using [**1**]$^+$ is as follows:

To a J Young NMR tube, a solution of [**1**][OTf] (10 mol%, 0.0057 mmol) in DMF (0.5 mL) and HBpin (0.057 mmol, 1.0 equiv.) was added. The reaction mixture was immediately frozen at 77 K. While frozen, the headspace of the NMR tube was evacuated. N_2O was then condensed into the NMR tube resulting in approximately 1 atm pressure at room temperature. The NMR tube was removed from the liquid nitrogen and after thawing, the reaction mixture was allowed to react for 8 hours under 390 nm light irradiation.

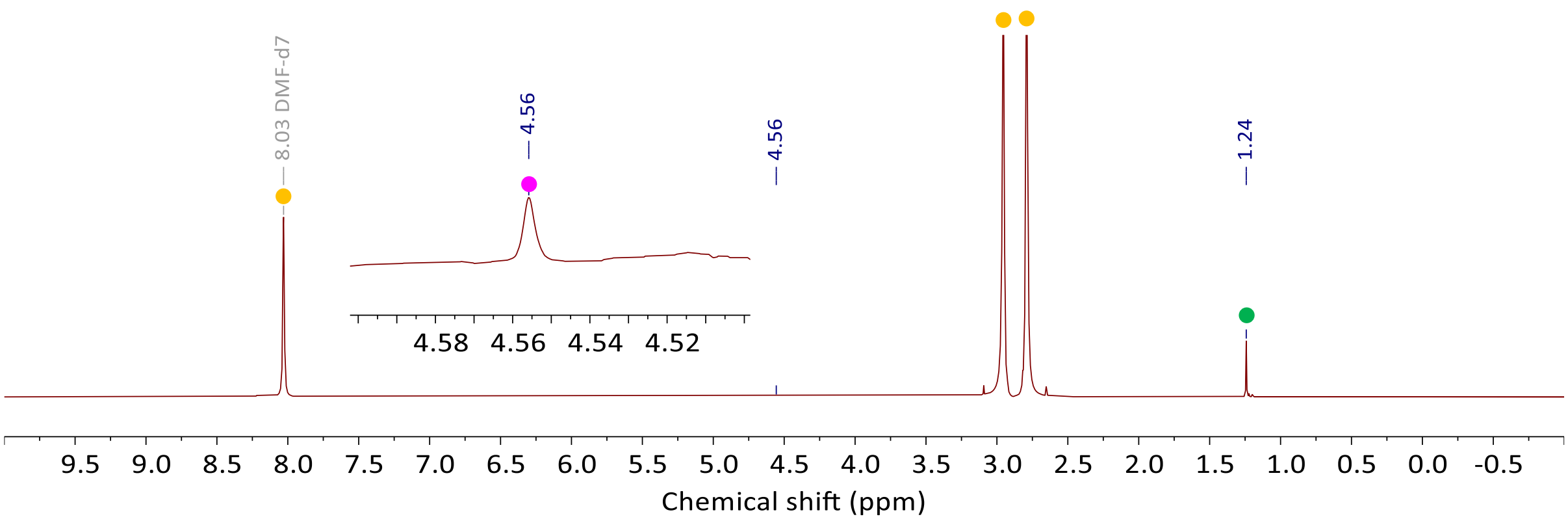

Figure S1: ^{1}H NMR spectrum (DMF) of reduction of N_2O with HBpin in DMF after 8 h using [**1**][OTf] (10 mol%) as a catalyst (optimised conditions) with an inset showing the H_2 resonance.

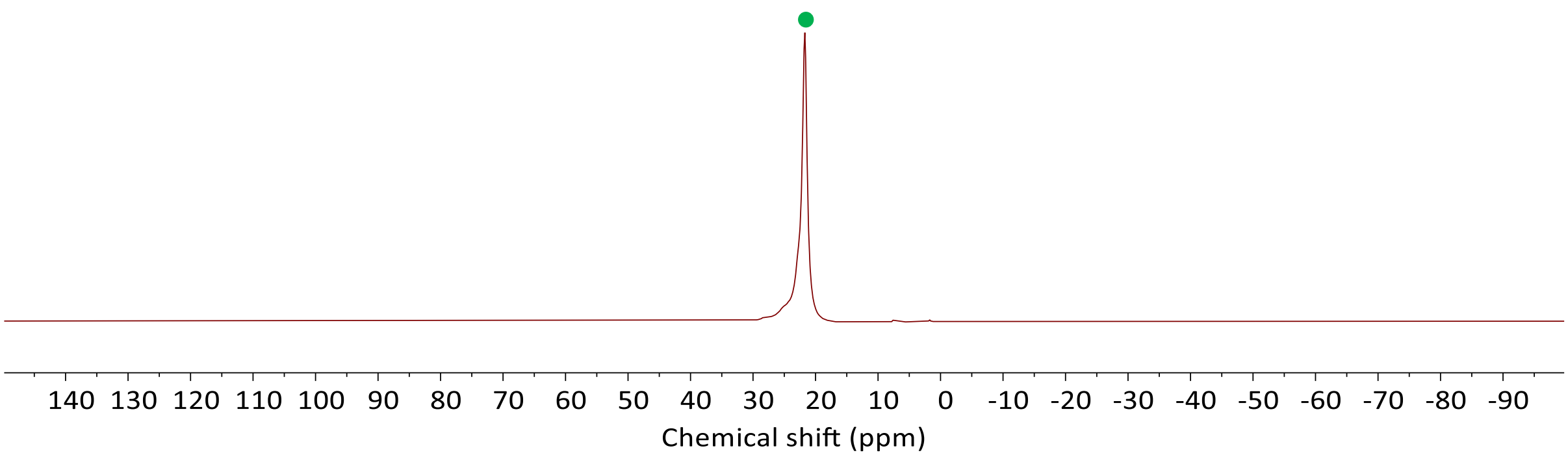

Figure S2: $^{11}B\{^{1}H\}$ NMR (DMF) of reduction of N_2O with HBpin in DMF after 8 h using [**1**][OTf] (10 mol%) as a catalyst (optimised conditions).

2.1.3 Tracked Reaction with [1][OTf]

A tracked reaction following the optimised procedure was undertaken to understand how the rate of reaction changes across the catalytic run. NMR conversions were determined by the ratio HBpin/product from the integrations of the crude ^{1}H and $^{11}B\{^{1}H\}$ NMR spectra.

Table S6: Reduction of N_2O with HBpin using the optimised conditions at specified time points.

**Time (h)**	**% Conversion (^{1}H NMR spectroscopy)**
1	6
2	15
4	44
8	100

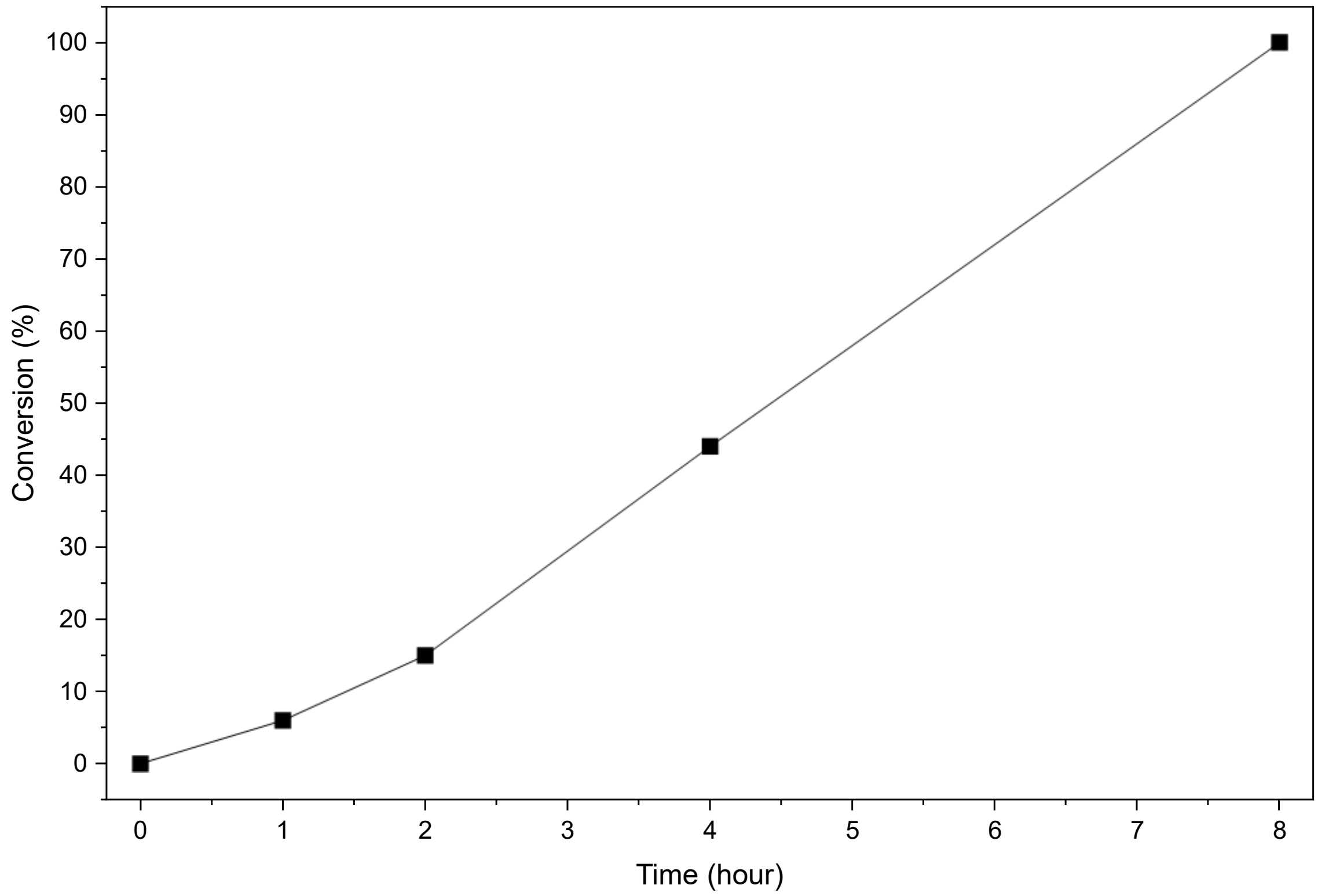

Figure S3: Tracked reaction for the reduction of N_2O with HBpin using the optimised conditions at specified time points from Table S6.

2.1.4 Control Reactions and Stoichiometric Reactions with [1][OTf]

A series of control reactions were undertaken according to the general procedure with details of each reaction noted in Table S7. These reactions indicate that both the catalyst [**1**]$^+$ and visible light irradiation are essential to the reaction progressing.

Table S7: Control reactions associated with the reduction of N_2O with HBpin. Reactions followed standard procedures using [**1**][OTf] as a catalyst.

Reaction Components	[1][OTf] Loading (mol%)	Solvent	Irradiation	Temp	Result
N_2O + HBpin	10	DMF	-	rt	No reaction
[**1**][OTf] + N_2O + HBpin	10	DMF	-	50 °C	No reaction
HBpin + N_2O	0	DMF	-	rt	No reaction
HBpin + N_2O	0	DMF	390 nm	<43°C	No reaction
HBpin + N_2O	0	CD_3CN	-	rt	No reaction
HBpin + N_2O	0	CD_3CN	390 nm	<43°C	No reaction
[**1**][OTf] + N_2O	10	CD_3CN	-	rt	No reaction
[**1**][OTf] + N_2O	10	CD_3CN	390 nm	<43°C	No reaction

A series of stoichiometric reactions were undertaken to probe the first step in the catalytic cycle following modified procedures:

Stoichiometric Reaction Between [1][OTf] and HBpin:

To a J Young NMR tube, a solution of [**1**][OTf] (0.057 mmol, 1 equiv.) in CD_3CN (0.5 mL) and HBpin (0.057 mmol, 1 equiv.) was added. The reaction mixture was allowed to react for 18 hours under ambient conditions or under 390 nm light irradiation. The reaction was monitored by ^1H and $^{11}B\{^1H\}$ NMR spectroscopy and no reaction was observed.

Stoichiometric Reaction Between [1][OTf] and N_2O:

To a J Young NMR tube, a solution of [**1**][OTf] (0.057 mmol) in CD_3CN (0.5 mL) was added. The reaction mixture was immediately frozen at 77 K. While frozen, the headspace of the NMR tube was evacuated. N_2O was then condensed into the NMR tube resulting in approximately 1 atm pressure at room temperature. The NMR tube was removed from the liquid nitrogen and after thawing, the reaction mixture was allowed to react for 18 hours under ambient conditions or under 390 nm light irradiation. The reaction was monitored by ^1H NMR spectroscopy showing no consumption of [**1**][OTf].

Table S8: Stoichiometric reactivity studies involving [**1**]$^+$ relevant to the reduction of N_2O with HBpin.

Reaction Components	Solvent	Irradiation	Result
[**1**][OTf] + HBpin (1:1)	CD_3CN	-	No reaction
[**1**][OTf] + HBpin (1:1)	CD_3CN	390 nm	No reaction
[**1**][OTf] + N_2O (0.057mmol:1atm)	CD_3CN	-	No reaction
[**1**][OTf] + N_2O (0.057mmol:1atm)	CD_3CN	390 nm	No reaction

2.1.5 UV-Vis studies with [1][OTf]

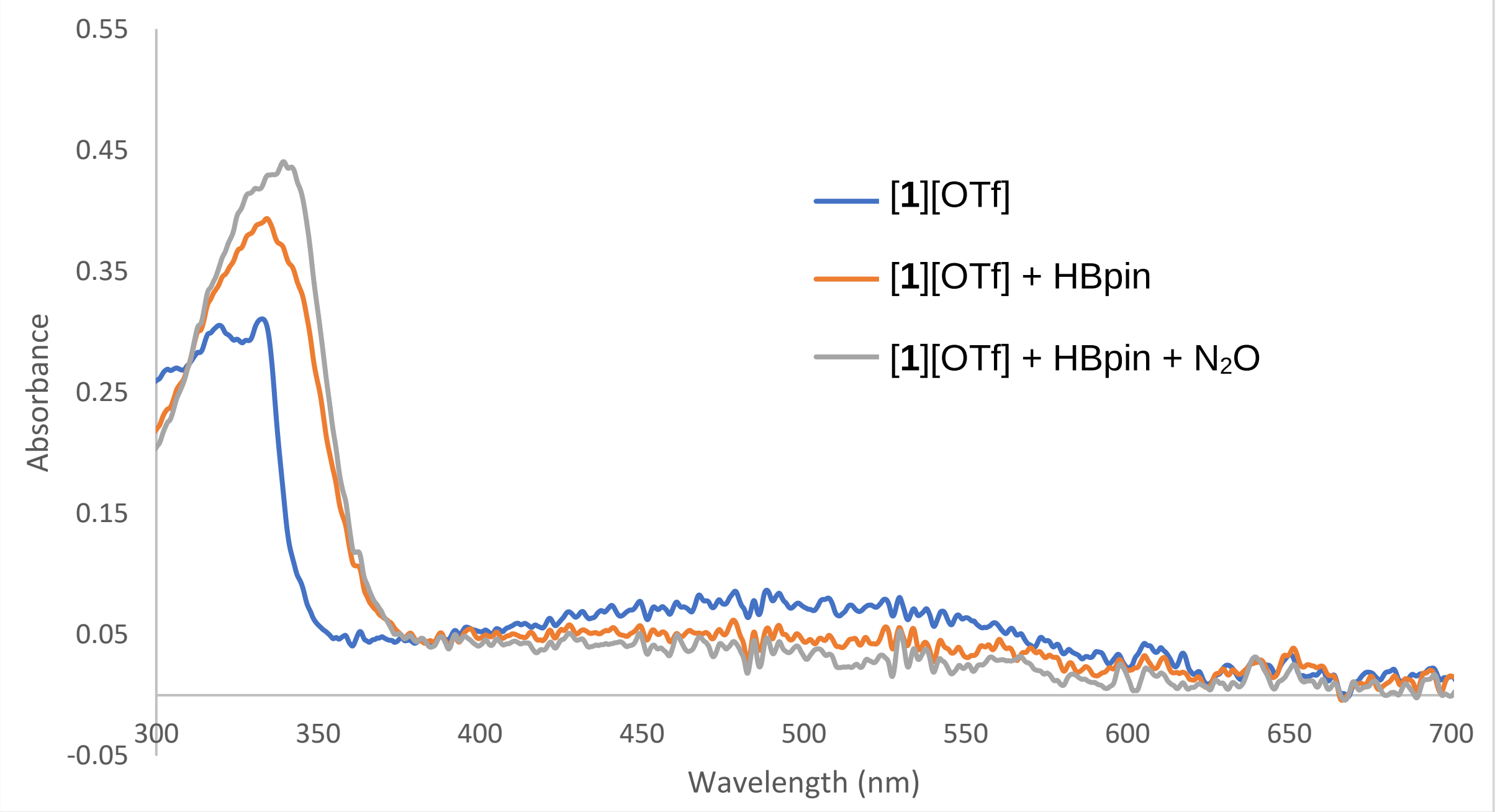

Figure S4: UV-Vis absorption spectra of [**1**][OTf], [**1**][OTf] + HBpin, and [**1**][OTf] + HBpin + N_2O at 0.01 mM in DMF solvent.

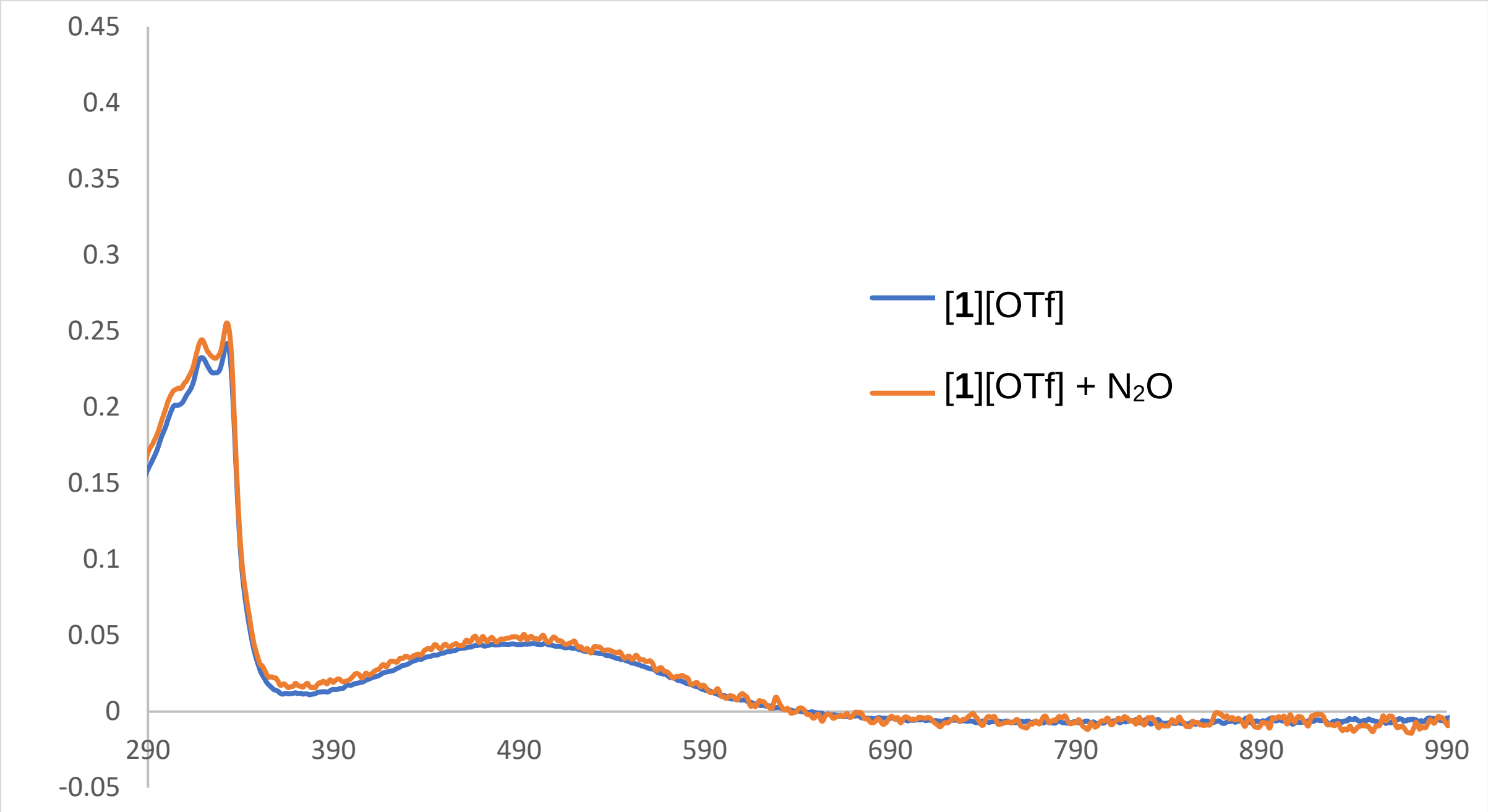

Figure S5: UV-Vis absorption spectra of [**1**][OTf] and [**1**][OTf] + N_2O at 0.01 mM in DMF solvent.

2.2 Optimisation and Control Reactions for 2

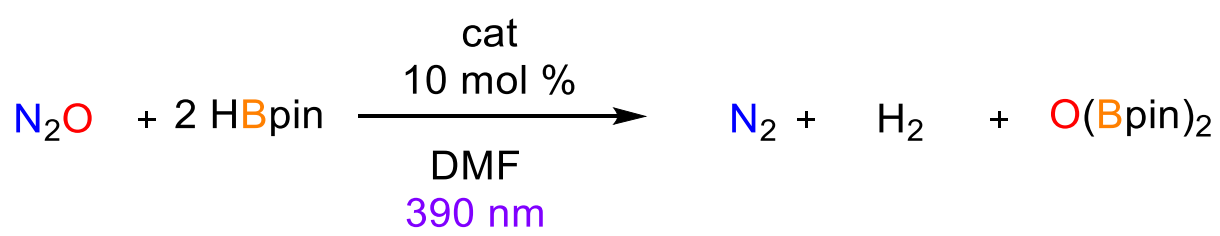

2.2.1 Initial Screening with 2 as the Catalyst

To a J Young NMR tube, a solution of **2** (0.0057 mmol, 10 mol%) in solvent (0.5 mL) and the reductant (0.057 mmol, 1.0 equiv.) was added. The reaction mixture was immediately frozen at 77 K. While frozen, the headspace of the NMR tube was evacuated. N_2O was then condensed into the NMR tube resulting in approximately 1 atm pressure at room temperature. The NMR tube was removed from the liquid nitrogen and after thawing, the reaction mixture was allowed to react for 2 hours under 390 nm irradiation. The reaction was monitored by ^1H and $^{11}B\{^1H\}$ NMR spectroscopy. NMR conversions were determined by the ratio HBpin/product from the integrations of the crude ^1H and $^{11}B\{^1H\}$ NMR spectra. Note: Due to trace amounts of water in the N_2O cylinder, a small HOBpin resonance (<5 %) can be seen in control reactions. For this reason, any conversion < 5 % is considered to be from this trace water and listed as <5 %.

Table S9: Solvent screening for reduction of N_2O with HBpin using **2** (10 mol%) as the catalyst. Reactions were conducted under 390 nm irradiation for 2 h.

Solvent	% Conversion (^1H NMR spectroscopy)	% Conversion ($^{11}B\{^1H\}$ NMR spectroscopy)
CD_2Cl_2	<5	<5
oDFB	<5	<5
DMF	>99	>99
THF	45	52
CD_3CN	54	60

Table S10: Reductant screening for the reduction of N_2O using **2** (10 mol%) as the catalyst. Reactions were conducted in DMF solvent under 390 nm irradiation for 2 h.

Reductant	Amount used	% Conversion (^1H NMR spectroscopy)
HBpin	8.9 µL	>99
Cyclohexadiene	5.4 µL	0
9,10-dihydroanthracene	10.3 mg	0
H_2	1 atm	0
B_2pin_2	9.4 mg	54*

***** The observed product was HOBpin.

The catalytic reaction was repeated using different irradiation wavelengths, and the catalytic loading was varied for the most effective wavelength.

To a J Young NMR tube, a solution of **2** (see Table S11 for catalytic loading) in DMF (0.5 mL) and HBpin (0.057 mmol, 1.0 equiv.) was added. The reaction mixture was immediately frozen at 77 K. While frozen, the headspace of the NMR tube was evacuated. N_2O was then condensed into the NMR tube resulting in approximately 1 atm pressure at room temperature. The NMR tube was removed from the liquid nitrogen and after thawing, the reaction mixture was allowed to react for 2 hours under visible light irradiation. The reaction was monitored by ^1H and $^{11}B\{^1H\}$ NMR spectroscopy. NMR conversions were determined by the ratio HBpin/product from the integrations of the crude ^1H and $^{11}B\{^1H\}$ NMR spectra. Note: Due to trace amounts of water in the N_2O cylinder, a small HOBpin resonance (<5 %) can be seen in control reactions. For this reason, any conversion <5 % is considered to be from this trace water and listed as <5 %.

Table S11: Wavelength screening for reduction of N_2O with HBpin using **2** as the catalyst. Reactions were conducted in DMF solvent for 2 h.

Wavelength (nm)	Loading (mol %)	% Conversion (^{1}H NMR spectroscopy)
390	10	>99
	5	43
	1	18
	1*	19
456	10	<5
525	10	<5

* Use of N_2O gas pre-dried over 3 Å molecular sieves.

2.2.2 Optimised Procedure for the Reduction of N_2O with HBpin using 2 as the catalyst.

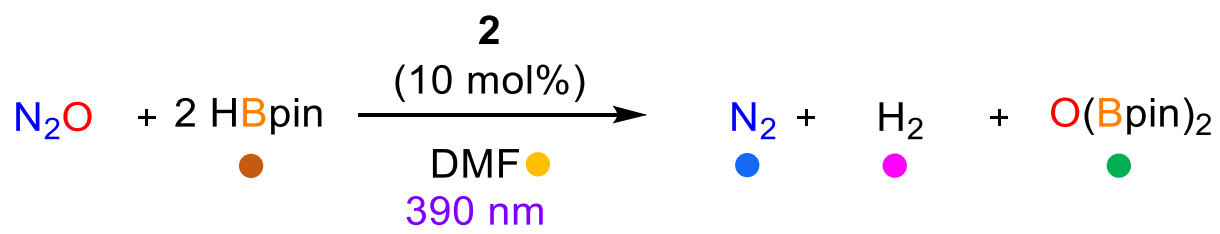

The optimised procedure for the reaction was as follows:

To a J Young NMR tube, a solution of **2** (10 mol%, 0.0057 mmol) in DMF (0.5 mL) and HBpin (0.057 mmol, 1.0 equiv.) was added. The reaction mixture was immediately frozen at 77 K. While frozen, the headspace of the NMR tube was evacuated. N_2O was then condensed into the NMR tube resulting in approximately 1 atm pressure at room temperature. The NMR tube was removed from the liquid nitrogen and after thawing, the reaction mixture was allowed to react for 2 hours under 390 nm light irradiation.

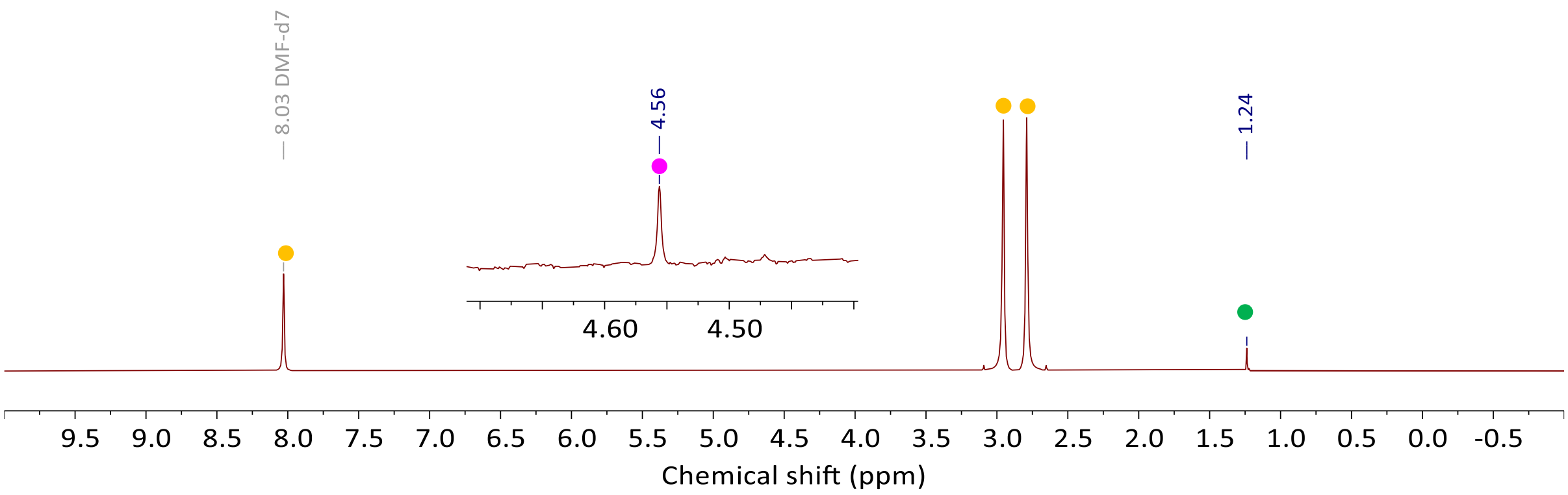

Figure S6: ^{1}H NMR spectrum (DMF) of the reduction of N_2O with HBpin in DMF after 2 h using **2** (10 mol%) as a catalyst.

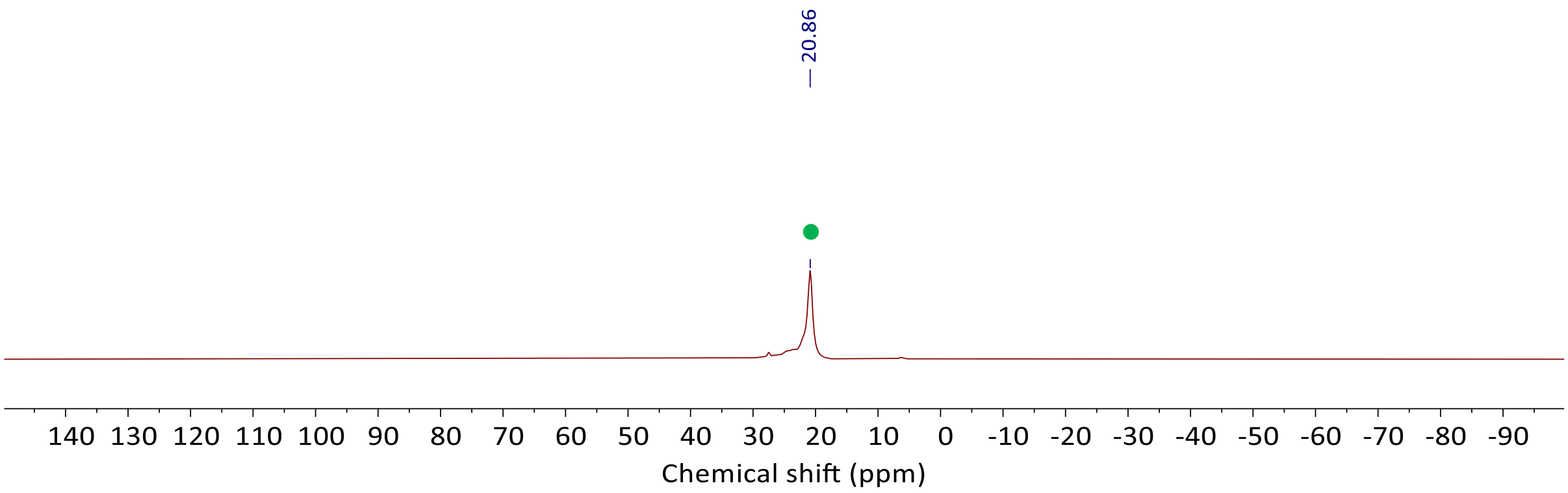

Figure S7: ^{11}B{^{1}H} NMR spectrum (DMF) of the reduction of N_2O with HBpin in DMF after 2 h using **2** (10 mol%) as a catalyst.

2.2.3 Control Reactions and Stoichiometric Reactions with 2.

A series of control reactions were undertaken according to the optimised general procedure described in Section 2.2.2 with changes to the standard conditions noted:

Table S12: Control reactions for the reduction of N_2O with HBpin using **2** as catalyst.

**Change to conditions**	**Solvent**	**Outcome**
No irradiation	CD_3CN	No reaction
No irradiation	DMF	No reaction
50 °C without irradiation	DMF	No reaction
Pyrrole catalyst	DMF	No reaction
2,2,6,6-tetramethylpiperidine catalyst	DMF	No reaction

2.2.4 UV-Vis spectrum of 2

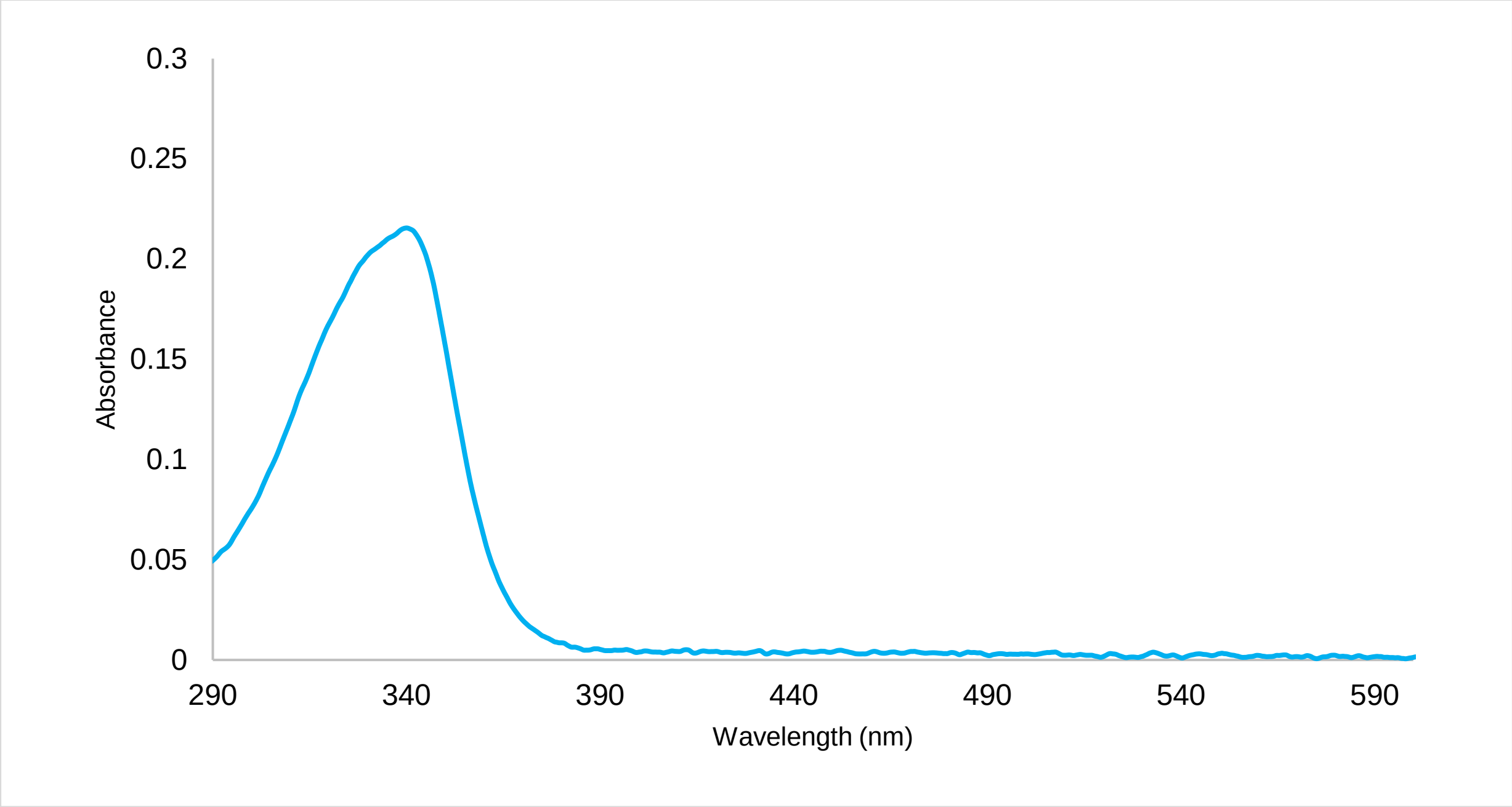

Figure S8: UV-Vis absorption spectrum of **2** (0.01 mM) in DMF solvent.

2.3 Labelled Experiments with 2

2.3.1 ^{15}N-N_2O

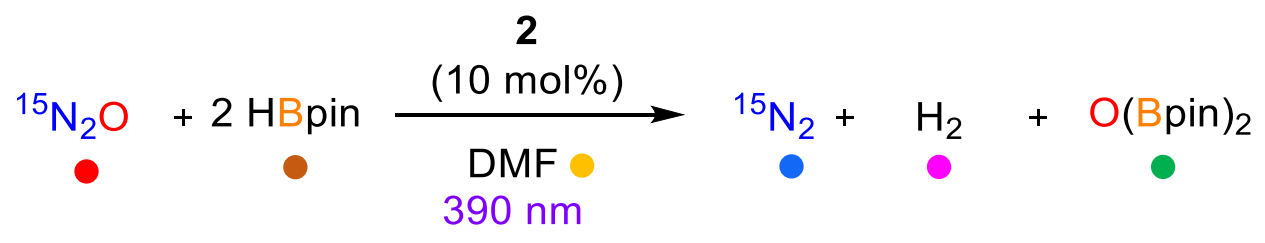

To a J Young NMR tube, a solution of **2** (0.029 mmol, 10 mol%) in DMF (0.5 mL) and HBpin (41.4 µL, 0.29 mmol, 1.0 equiv.) were added. The reaction mixture was immediately frozen at 77 K. While frozen, the headspace of the NMR tube was evacuated. N_2O was then condensed into the NMR tube resulting in approximately 1 atm pressure at room temperature. The NMR tube was removed from the liquid nitrogen and after thawing, the reaction mixture was allowed to react for 2 hours under 390 nm irradiation. The reaction was monitored by ^{1}H, ^{11}B{^{1}H}, and ^{15}N NMR spectroscopy.

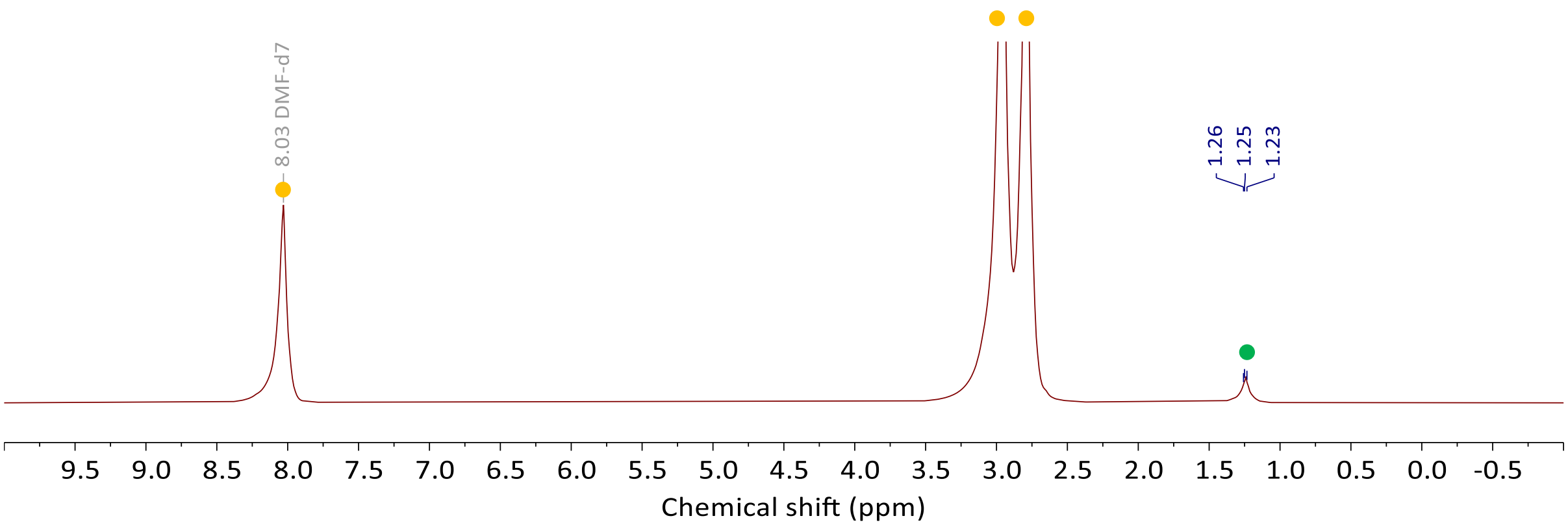

Figure S9: ^{1}H NMR spectrum (DMF) of reaction 2.3.1.

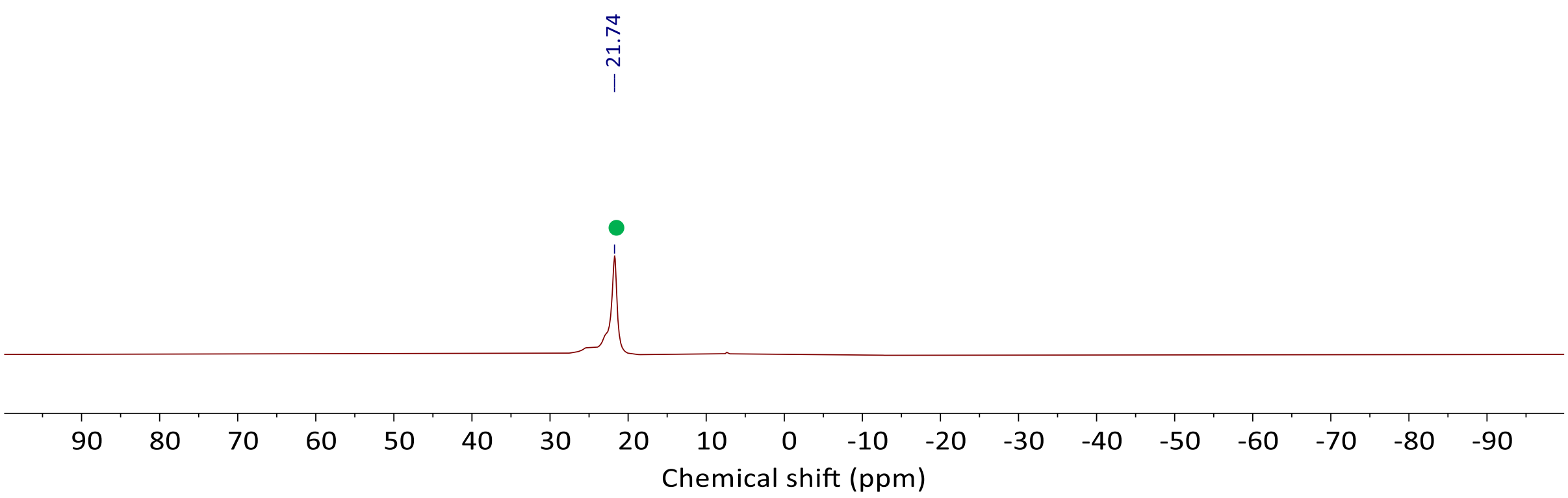

Figure S10: ^{11}B{^{1}H} NMR spectrum (DMF) of reaction 2.3.1.

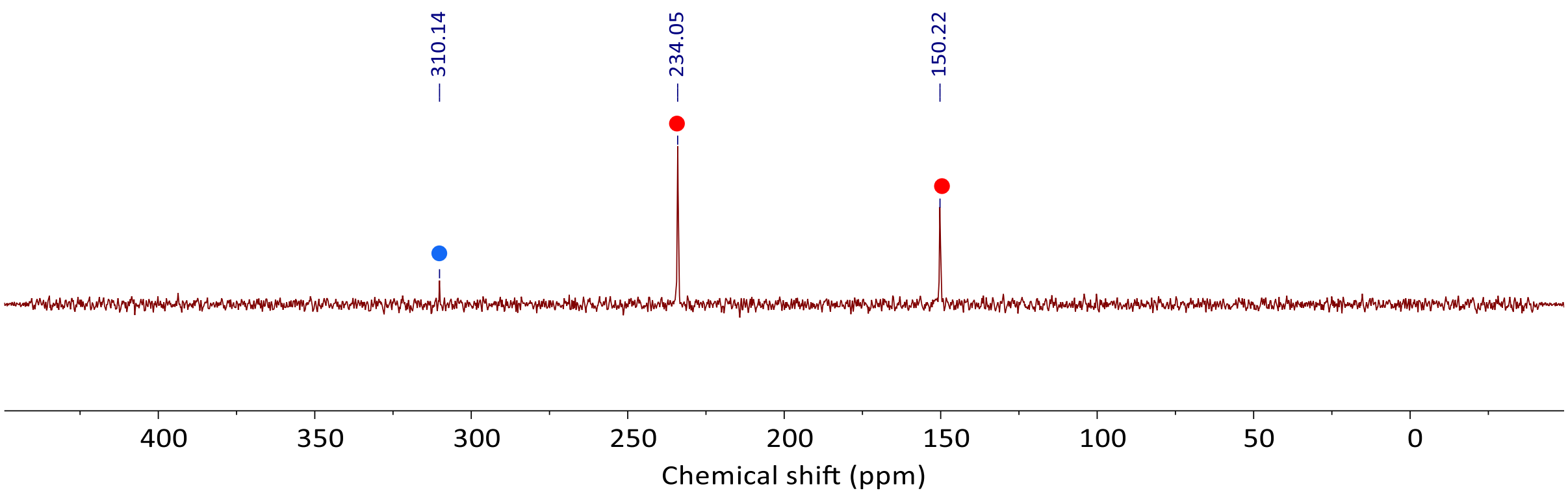

Figure S11: ^{15}N NMR spectrum (DMF) of reaction 2.3.1.

2.3.2 DBpin

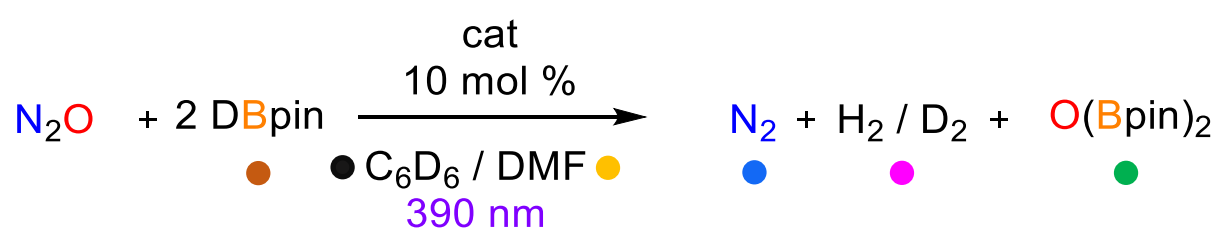

To a J Young NMR tube, a solution of **2** (1.1 mg, 0.0057 mmol, 10 mol%) in DBpin (from a 1 x 10^{-4} mmol/µL stock solution in C_6D_6; 407 µL, 0.057 mmol, 1.0 equiv.) and DMF (0.1 mL) were added. The reaction mixture was immediately frozen at 77 K. While frozen, the headspace of the NMR tube was evacuated. N_2O was then condensed into the NMR tube resulting in approximately 1 atm pressure at room temperature. The NMR tube was removed from the liquid nitrogen and after thawing, the reaction mixture was allowed to react for 2 hours under 390 nm irradiation. The reaction was monitored by ^{1}H, $^{11}B\{^{1}H\}$, and ^{2}H NMR spectroscopy.

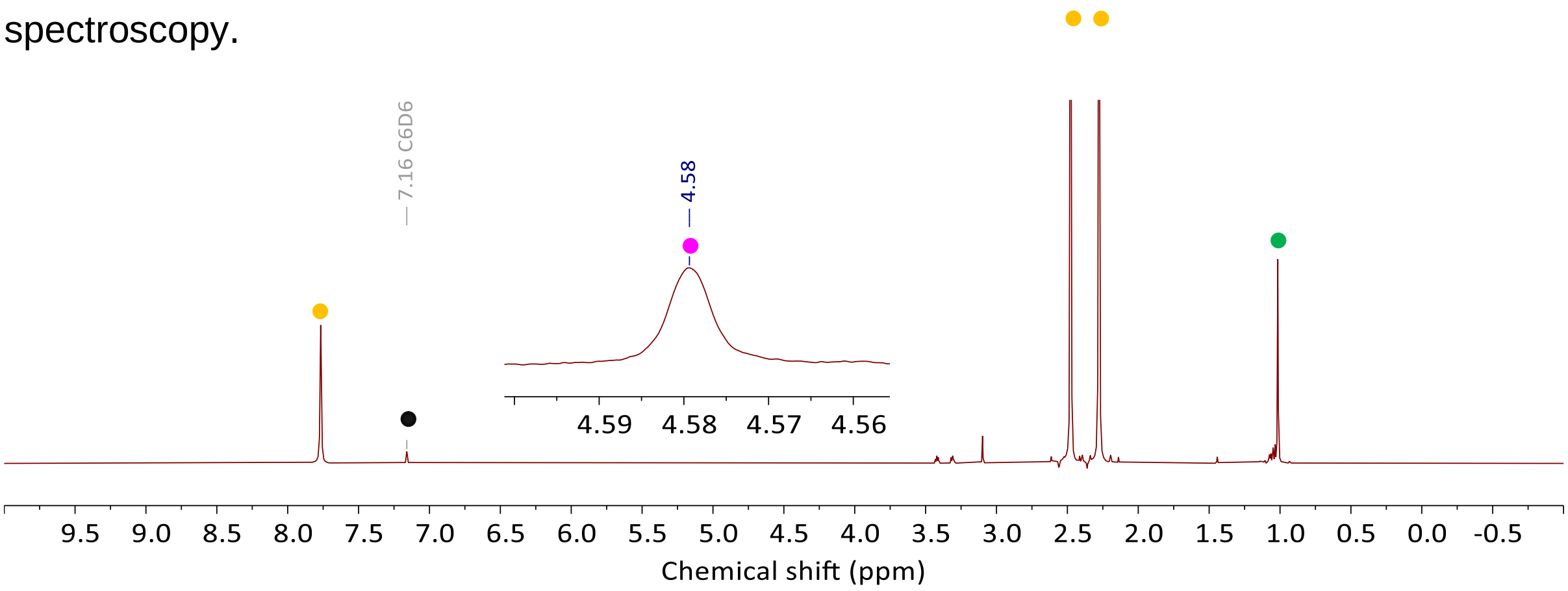

Figure S12: ^{1}H NMR spectrum (C_6D_6:DMF) of reaction 2.3.2 with an inset showing the H_2 resonance.

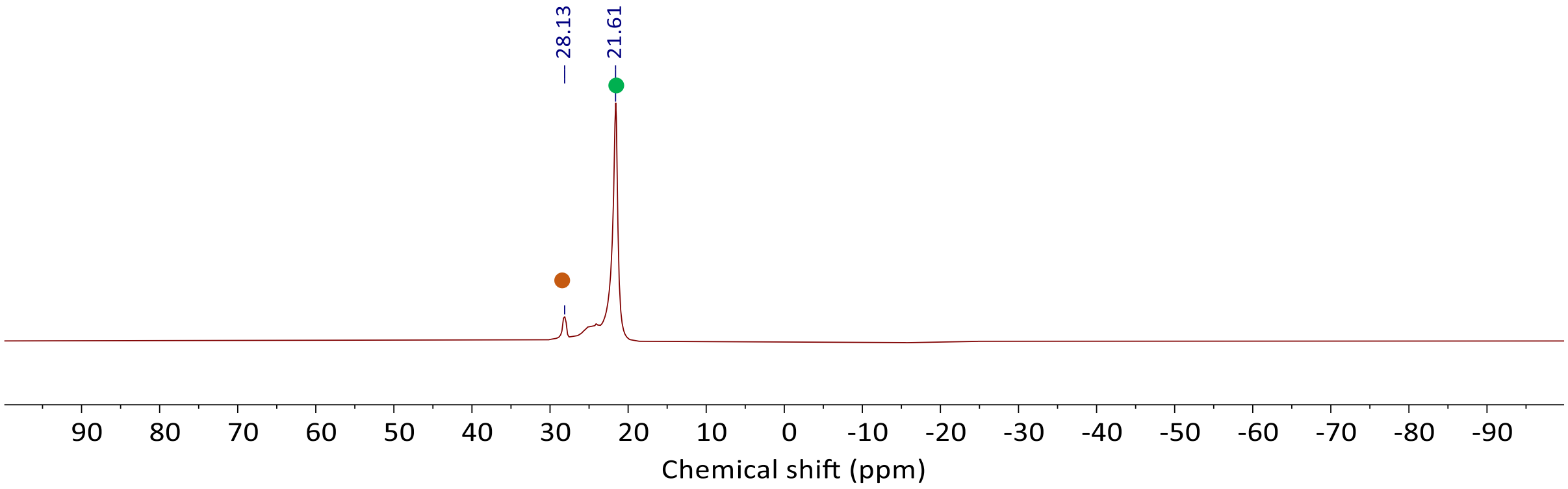

Figure S13: $^{11}B\{^{1}H\}$ NMR spectrum (C_6D_6:DMF) of reaction 2.3.2.

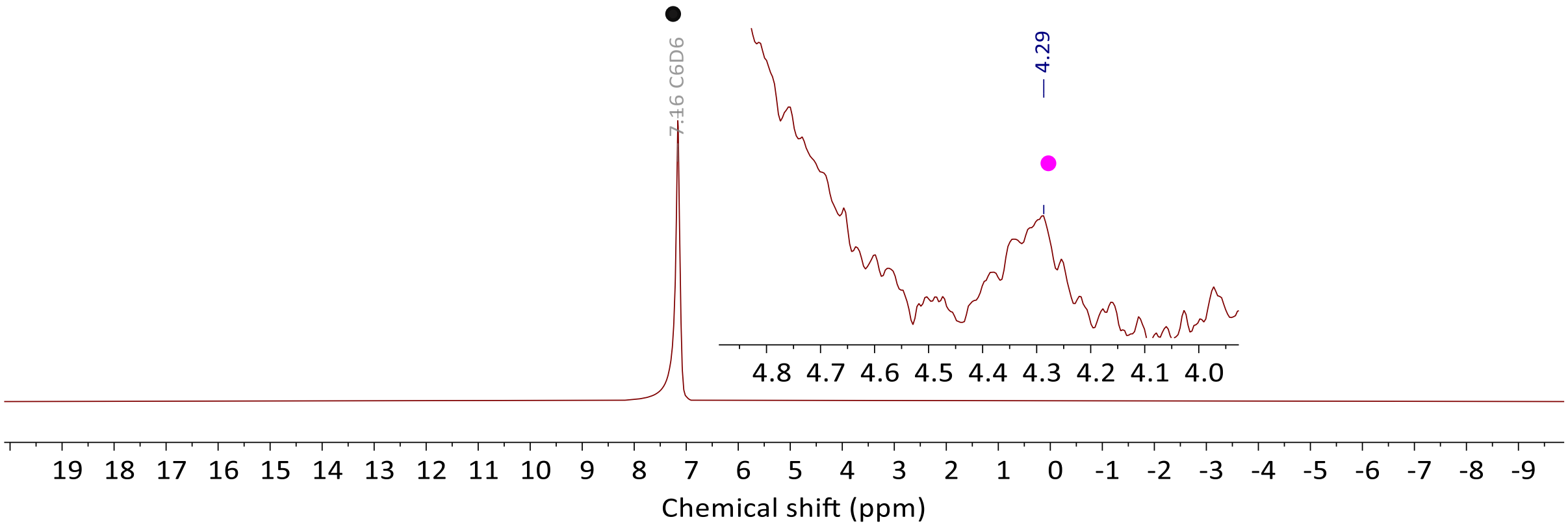

Figure S14: ^{2}H NMR spectrum (C_6D_6:DMF) of reaction 2.3.2 with an inset showing the D_2 resonance.

2.3.3 DMF-d$_7$

2.3.3.1 Catalytic Loading of **2**

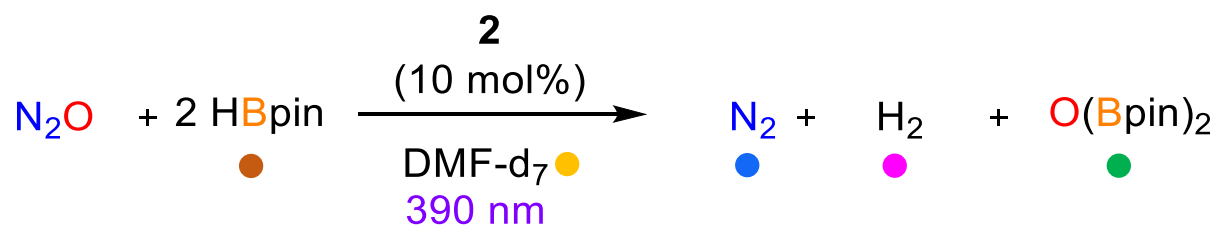

To a J Young NMR tube, a solution of **2** (1.1 mg, 0.0057 mmol, 10 mol%) in DMF-d$_7$ (0.5 mL) and HBpin (8.3 µL, 0.057 mmol, 1.0 equiv.) were added. The reaction mixture was immediately frozen at 77 K. While frozen, the headspace of the NMR tube was evacuated. N_2O was then condensed into the NMR tube resulting in approximately 1 atm pressure at room temperature. The NMR tube was removed from the liquid nitrogen and after thawing, the reaction mixture was allowed to react for 2 hours under 390 nm irradiation. The reaction was monitored by ^{1}H NMR spectroscopy where no HD resonance was observed.

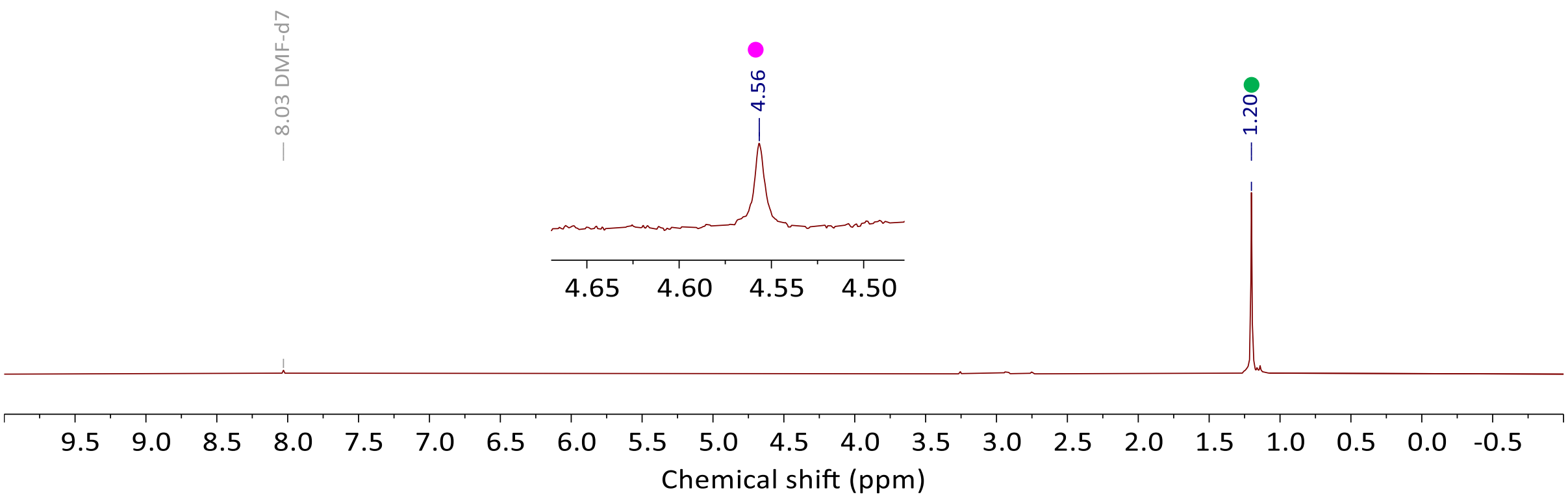

Figure S15: ^{1}H NMR (DMF-d$_7$) spectrum of reaction 2.3.3.1 with an inset showing the H_2 resonance.

2.3.3.2 Stoichiometric Loading of **2**

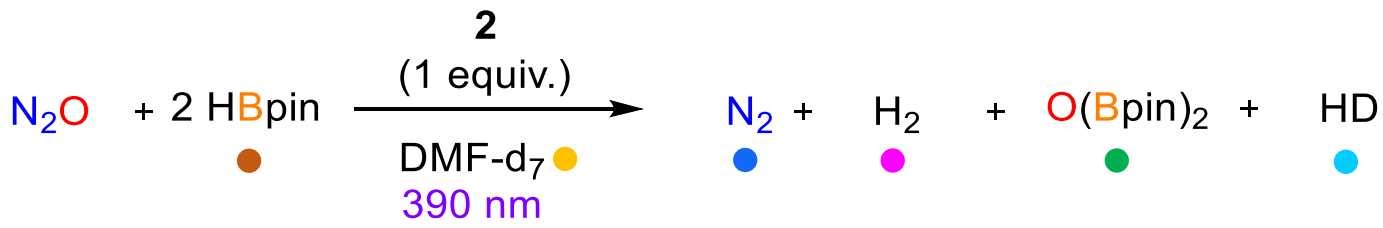

To a J Young NMR tube, a solution of **2** (11 mg, 0.057 mmol, 1 equiv.) in DMF-d$_7$ (0.5 mL) and HBpin (8.3 µL, 0.057 mmol, 1.0 equiv.) was added. The reaction mixture was immediately frozen at 77 K. While frozen, the headspace of the NMR tube was evacuated. N_2O was then condensed into the NMR tube resulting in approximately 1 atm pressure at room temperature. The NMR tube was removed from the liquid nitrogen and after thawing, the reaction mixture was allowed to react for 2 hours under 390 nm irradiation. The reaction was monitored by ^{1}H and ^{11}B{^{1}H} NMR spectroscopy.

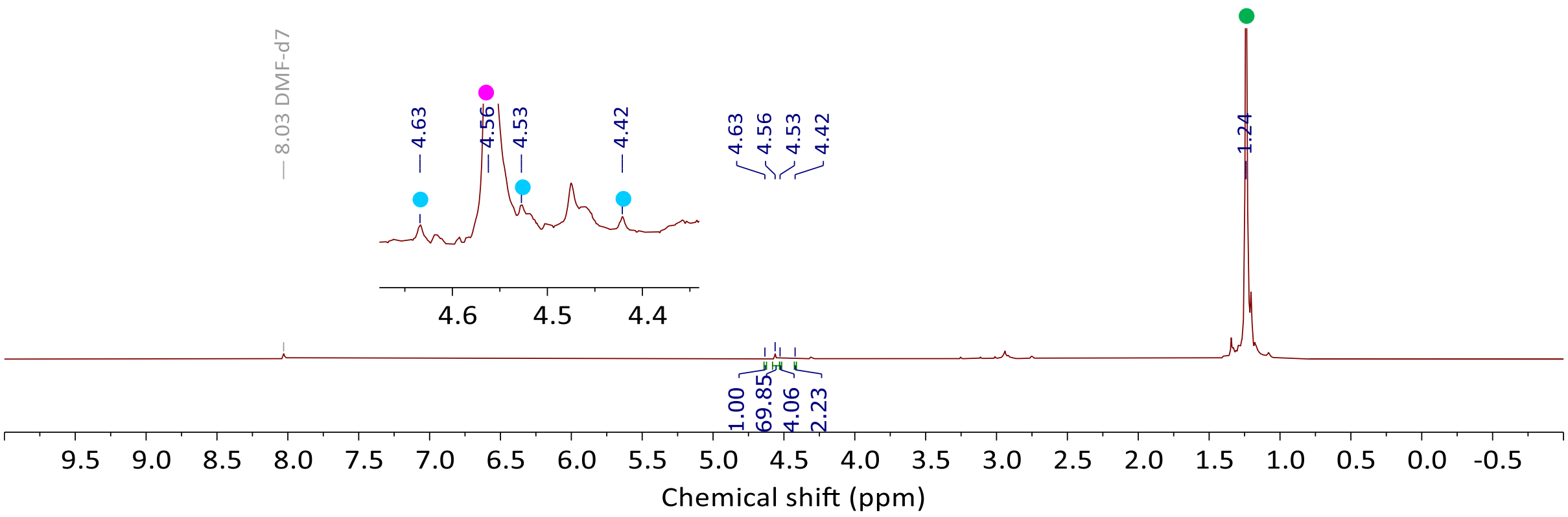

Figure S16: ^{1}H NMR (DMF-d$_7$) spectrum of reaction 2.3.3.2.

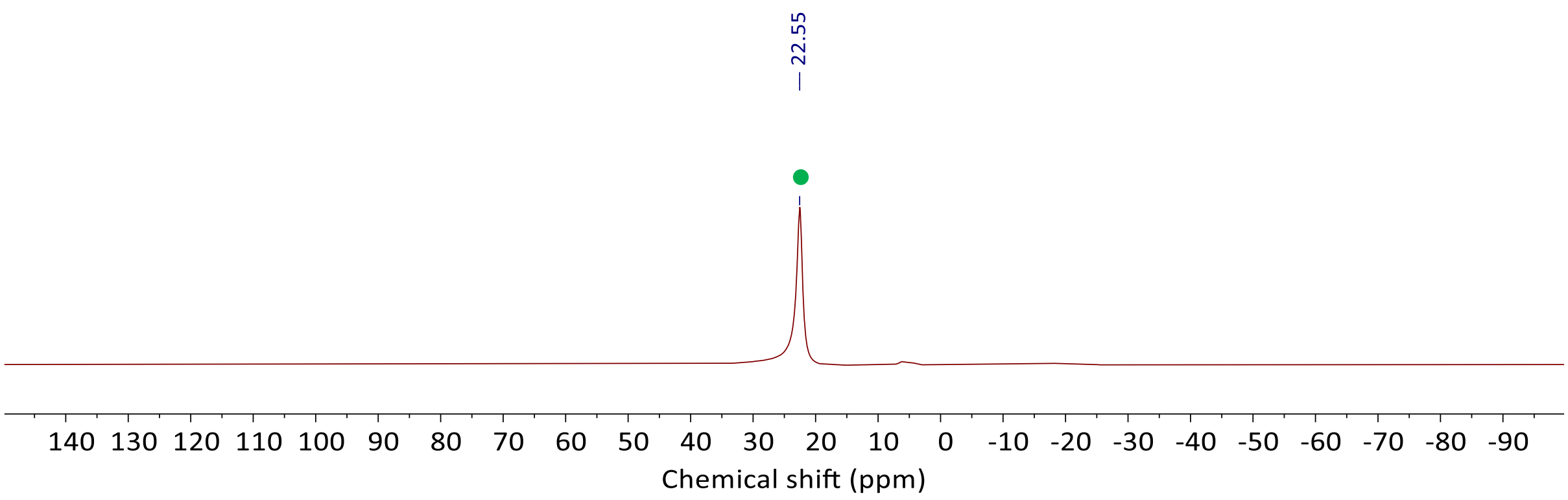

Figure S17: $^{11}B\{^1H\}$ NMR (DMF-d_7) spectrum of reaction 2.3.3.2.

2.4 Tracked Interval Reaction

To a J Young NMR tube, a solution of **2** (0.0057 mmol, 10 mol%) in DMF (0.5 mL) and HBpin (0.057 mmol, 1.0 equiv.) were added. The reaction mixture was immediately frozen at 77 K. While frozen, the headspace of the NMR tube was evacuated. N_2O was then condensed into the NMR tube resulting in approximately 1 atm pressure at room temperature. The NMR tube was removed from the liquid nitrogen and after thawing, the reaction mixture was left at room temperature for one hour before analysis by ^1H and $^{11}B\{^1H\}$ NMR spectroscopy. The reaction mixture was then subjected alternately to 390 nm irradiation and darkness in 20-minute intervals, with analysis by ^1H and $^{11}B\{^1H\}$ NMR spectroscopy after each interval.

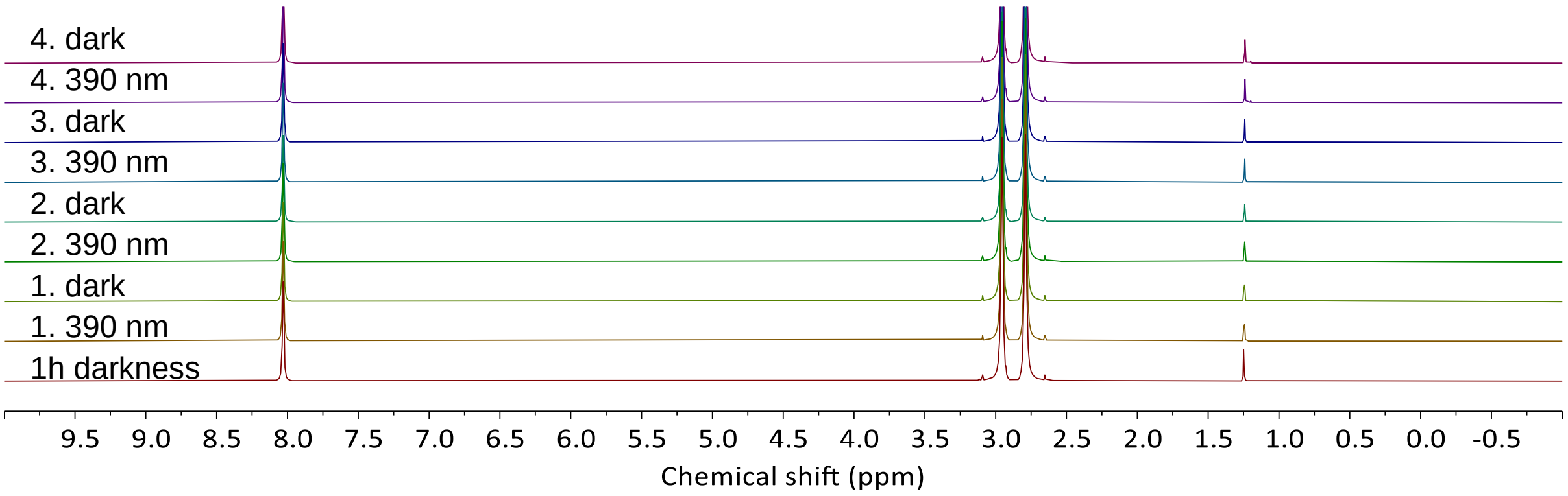

Figure S18: ^1H NMR spectra (DMF) of the reaction mixture at 20-minute time intervals.

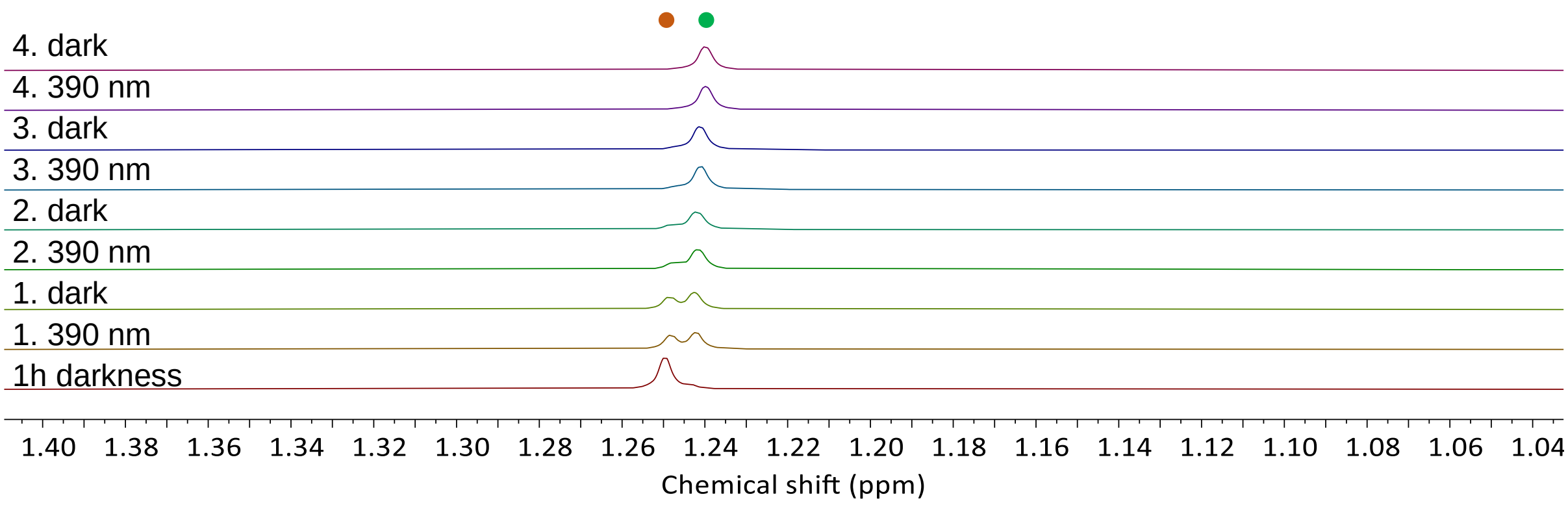

Figure S19: Zoomed in ^{1}H NMR spectra (DMF) of the reaction mixture at 20-minute time intervals. Orange dot: HBpin. Green dot: $O(Bpin)_2$.

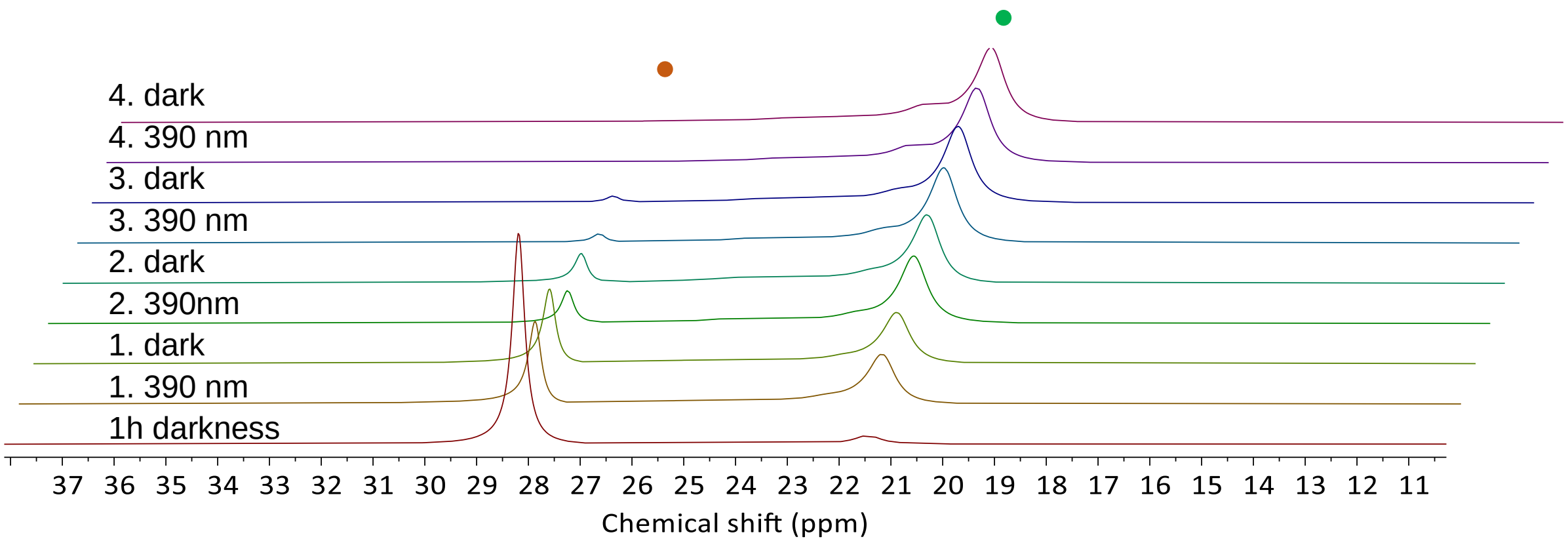

Figure S20: $^{11}B\{^{1}H\}$ NMR spectra (DMF, stacked representation) of the reaction mixture at 20-minute time intervals. Orange dot: HBpin. Green dot: $O(Bpin)_2$.

Table S13: Conversion at various time points in the reaction determined by $^{11}B\{^{1}H\}$ NMR spectroscopy.

**Time point (min)**	**Total Irradiation Time (min)**	**% Conversion ($^{11}B\{^{1}H\}$ NMR spectroscopy)**
60	0	<5
80	20	40
100	20	40
120	40	70
140	40	70
160	60	89
180	60	89
200	80	>99

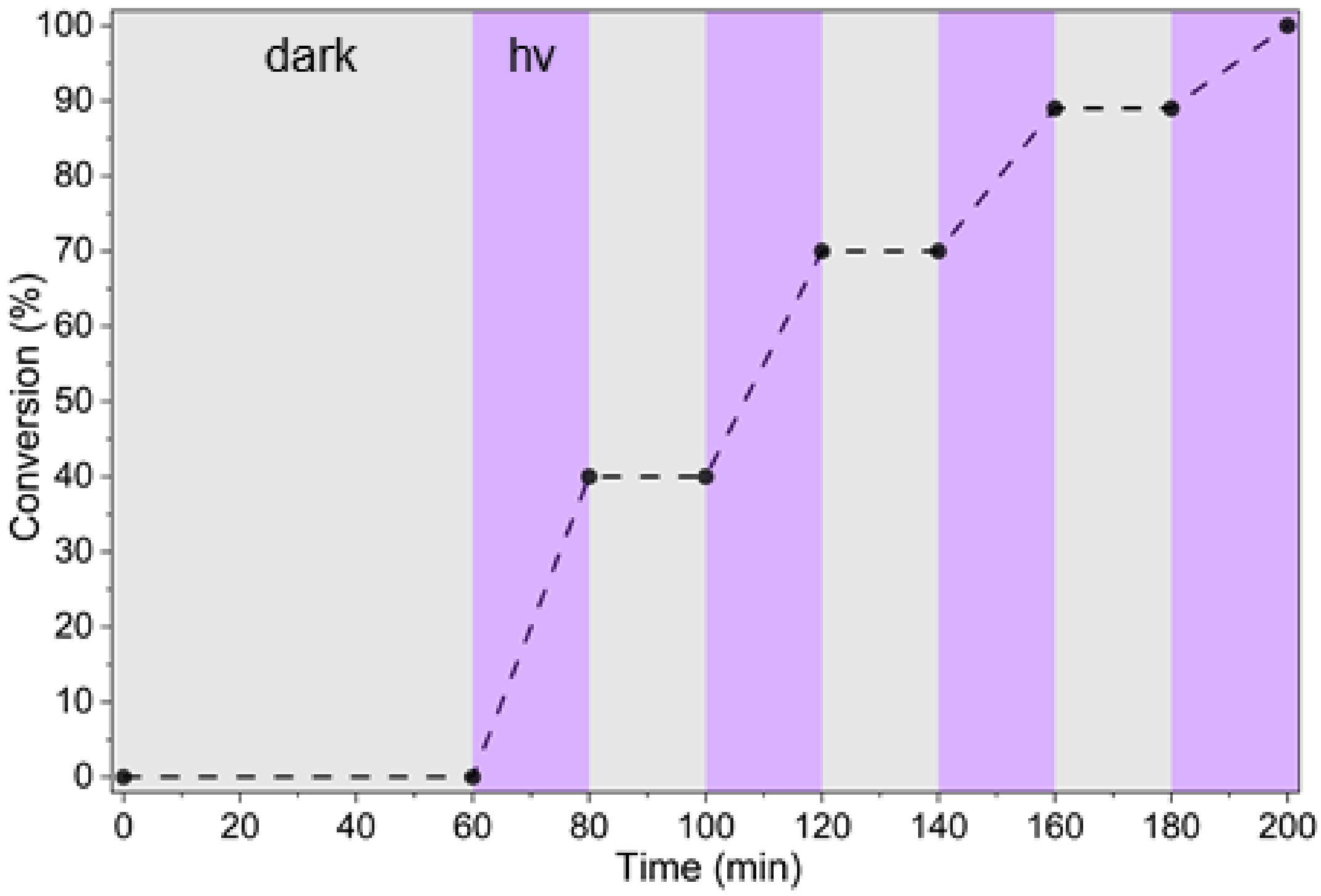

Figure S21: Graph tracking conversion of $O(Bpin)_2$ determined by ^1H NMR spectroscopy after oscillating periods of 20 minutes in the dark and under 390 nm irradiation.

2.5 Catalyst Stability Towards Excess Substrate

2.5.1 Catalyst Stability Towards Excess N_2O

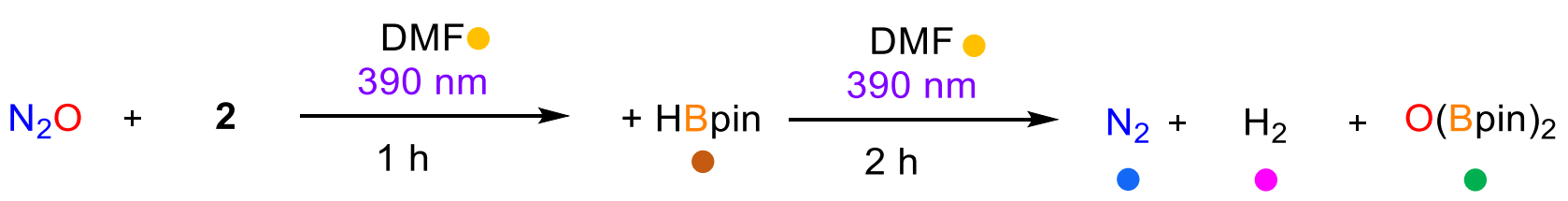

In a J Young NMR tube, a solution of **2** (0.0057 mmol, 10 mol%) in DMF (0.5 mL) was frozen at 77 K and the headspace was removed. The solution was thawed and the tube was refilled with N_2O (1 atm) before irradiating for 1 h under 390 nm light. The reaction mixture was then frozen at 77 K and HBpin (0.057 mmol, 1.0 equiv.) was added. The solution was irradiated with 390 nm light for a further 2 h and the product was analysed by ^1H and $^{11}B\{^1H\}$ NMR spectroscopy.

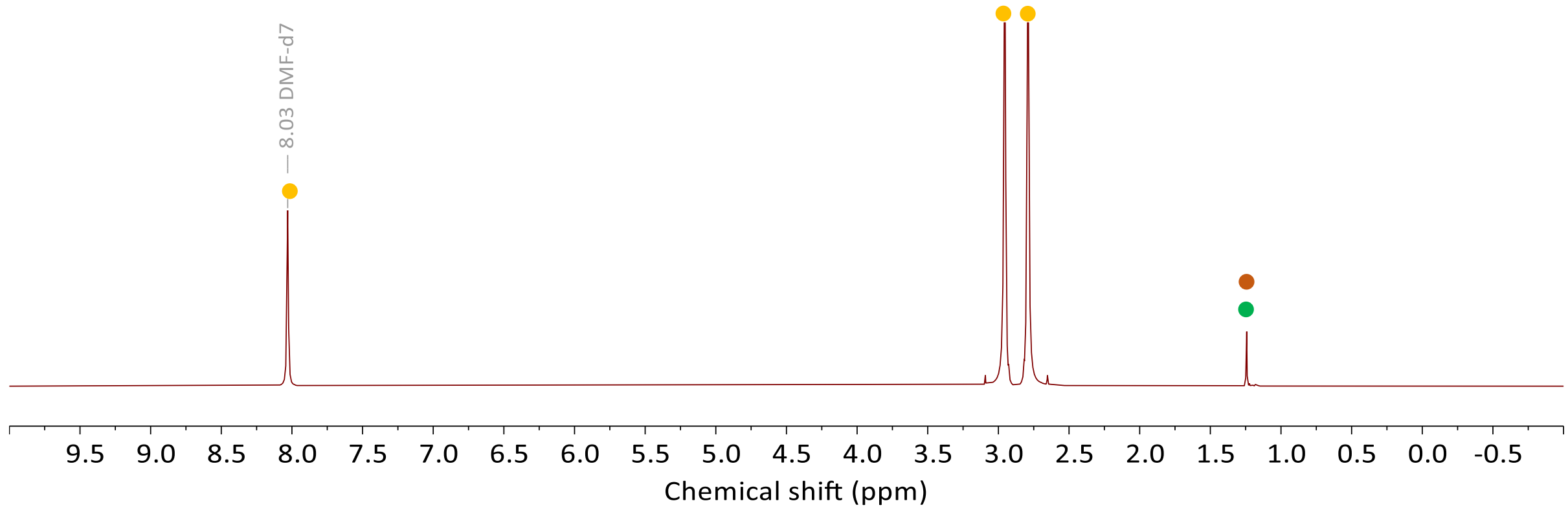

Figure S22: ^{1}H NMR spectrum (DMF) of reaction 2.5.1 after the second irradiation period.

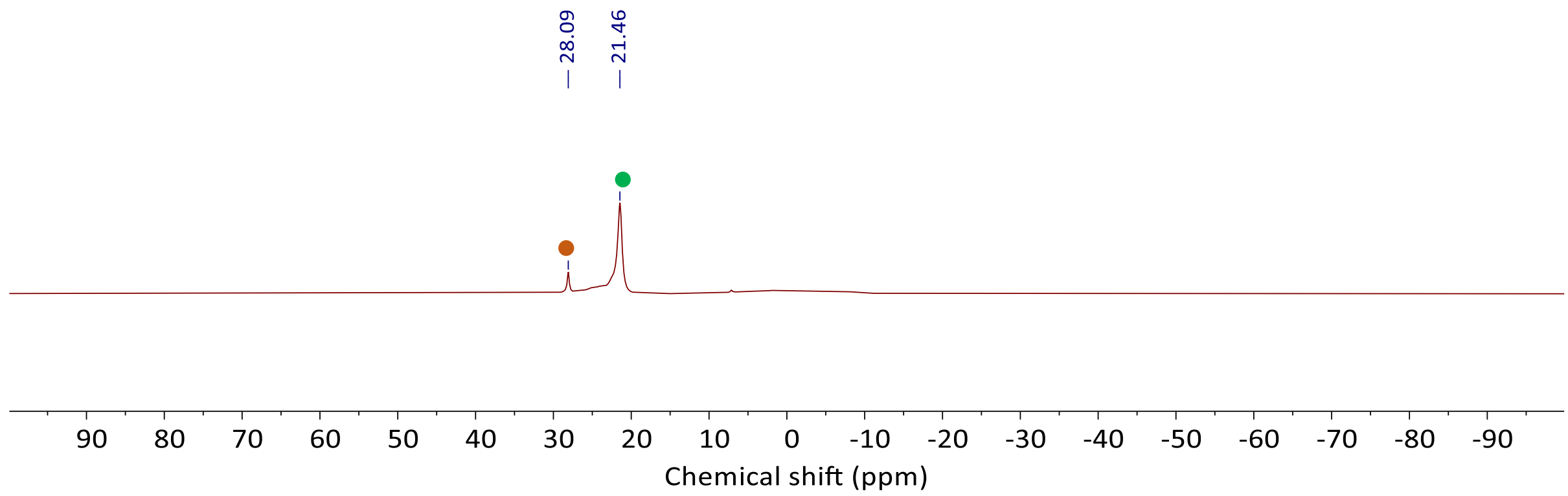

Figure S23: $^{11}B\{^{1}H\}$ NMR spectrum (DMF) of reaction 2.5.1 after the second irradiation period.

2.5.2 Catalyst Stability Towards Excess HBpin

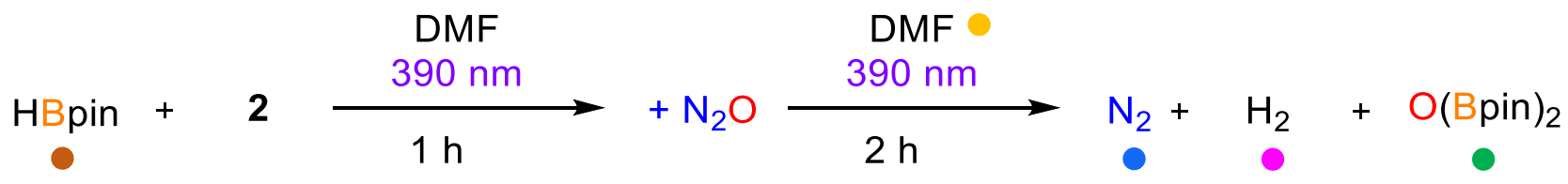

In a J Young NMR tube, **2** (0.0057 mmol, 0.1 equiv.) and HBpin (0.057 mmol, 1.0 equiv.) were dissolved in DMF (0.5 mL). The reaction mixture was irradiated for 1 h under 390 nm light before freezing at 77 K and removing the headspace. The solution was thawed and the tube was refilled with N_2O (1 atm) before irradiating for a further 2 h. The product was analysed by ^{1}H and $^{11}B\{^{1}H\}$ NMR spectroscopy.

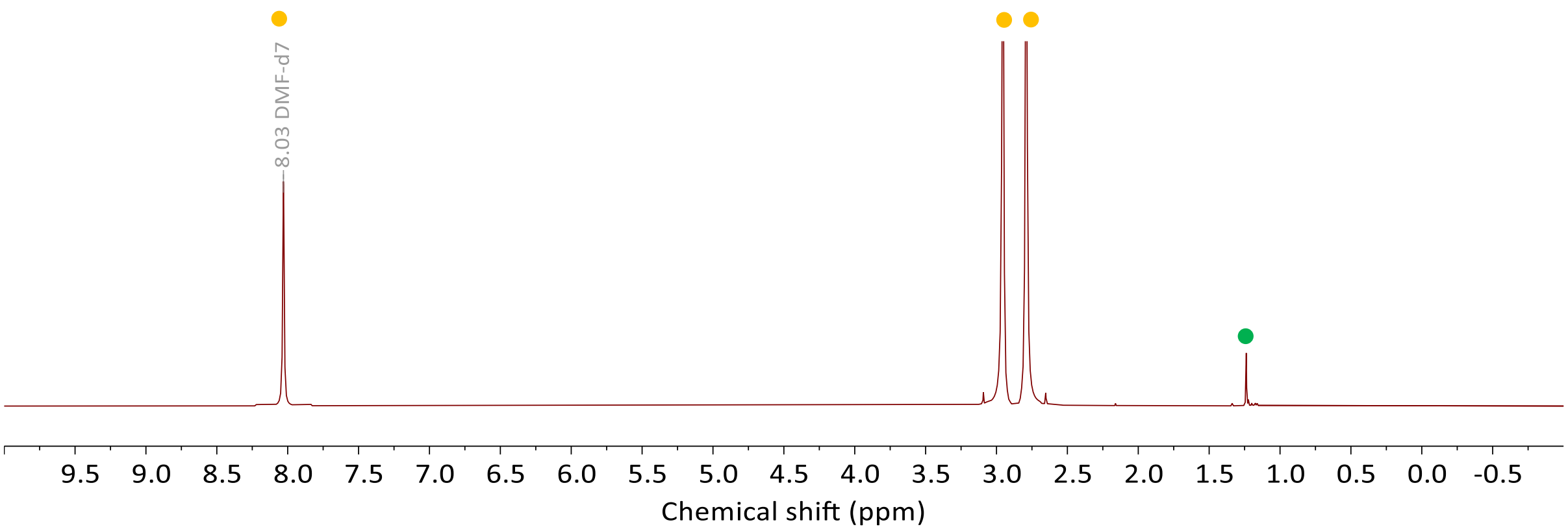

Figure S24: ^{1}H NMR spectrum (DMF) of reaction 2.5.2 after the second irradiation period.

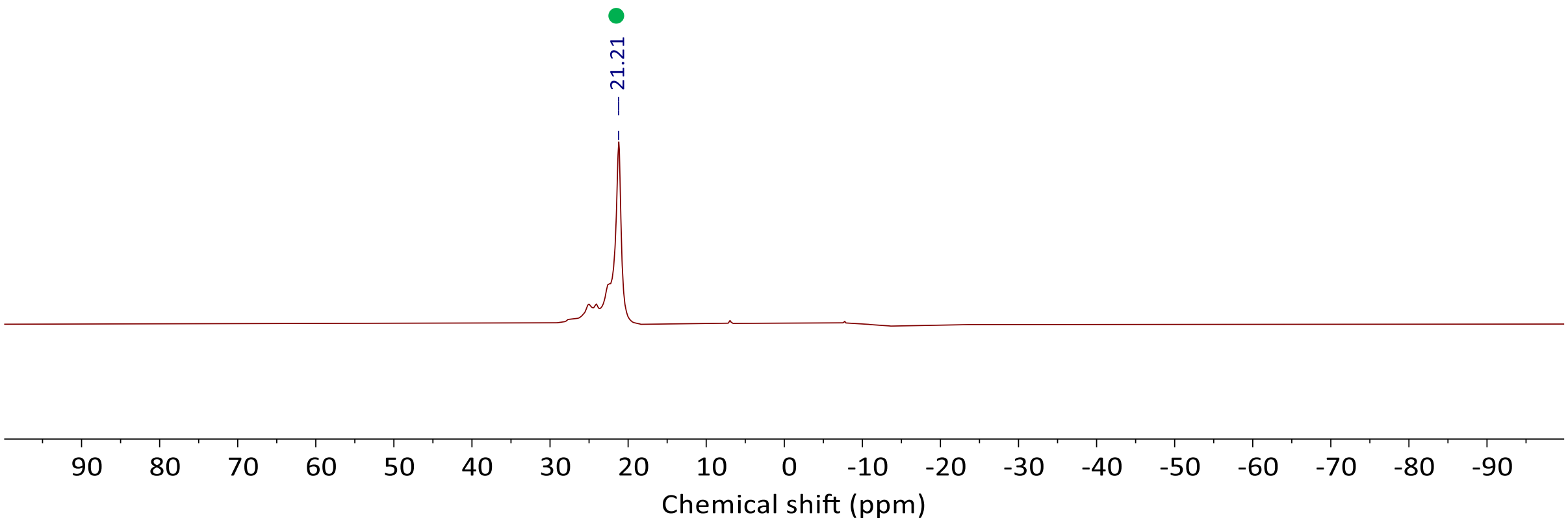

Figure S25: ^{11}B{^{1}H} NMR spectrum of 2.5.2 after the second irradiation period.

3. Experimental Mechanistic Studies

In addition to the stoichiometric and control reactions noted in Section 2.2 and 2.1.4, further targeted experiments were undertaken to understand individual components of the mechanism.

3.1 Studies on the homolytic cleavage of the N–H bond in 2

In a J Young NMR tube, **2** was dissolved in CD_3CN and irradiated for one hour under 390 nm light. The reaction was analysed by ^{1}H NMR spectroscopy, showing complete consumption of **2**, with small amounts of baseline decomposition visible. The NMR tube was left for 3 days under ambient conditions, showing decomposition of the radical into numerous products, as well as confirming the presence of H_2 in the NMR tube. This reaction was repeated in an EPR tube and analysed by EPR spectroscopy, showing formation of an EPR resonance that aligns with literature reports of **3**$^{\cdot}$.[4]

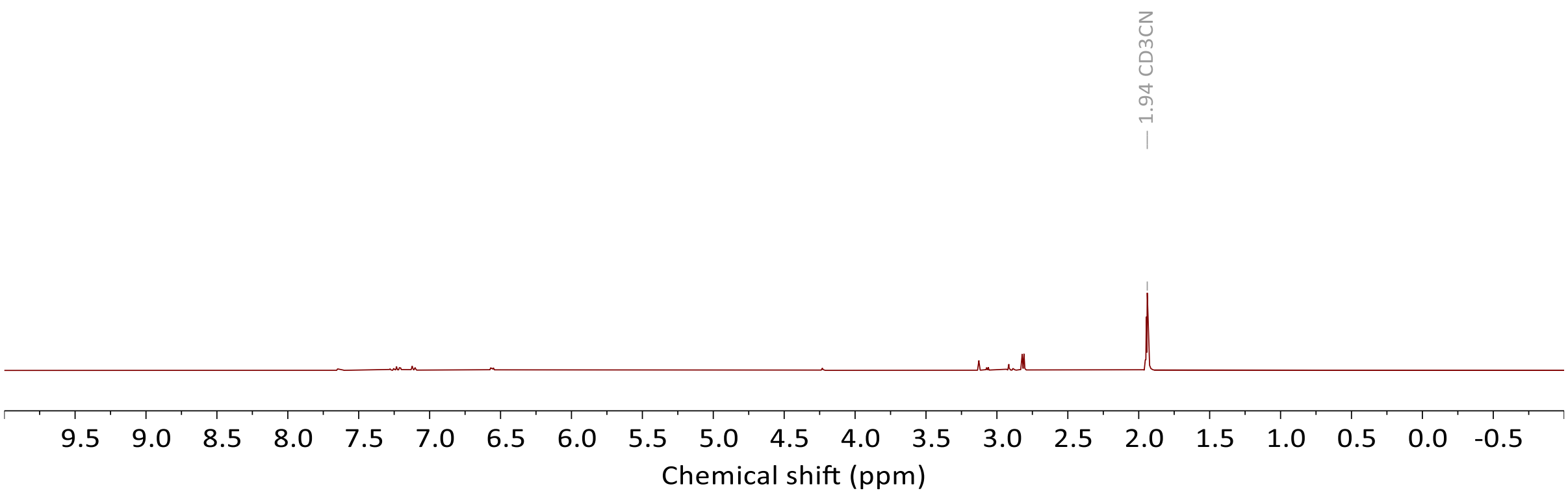

Figure S26: ^{1}H NMR spectrum (CD_3CN) of **2** after irradiation for one hour with 390 nm light.

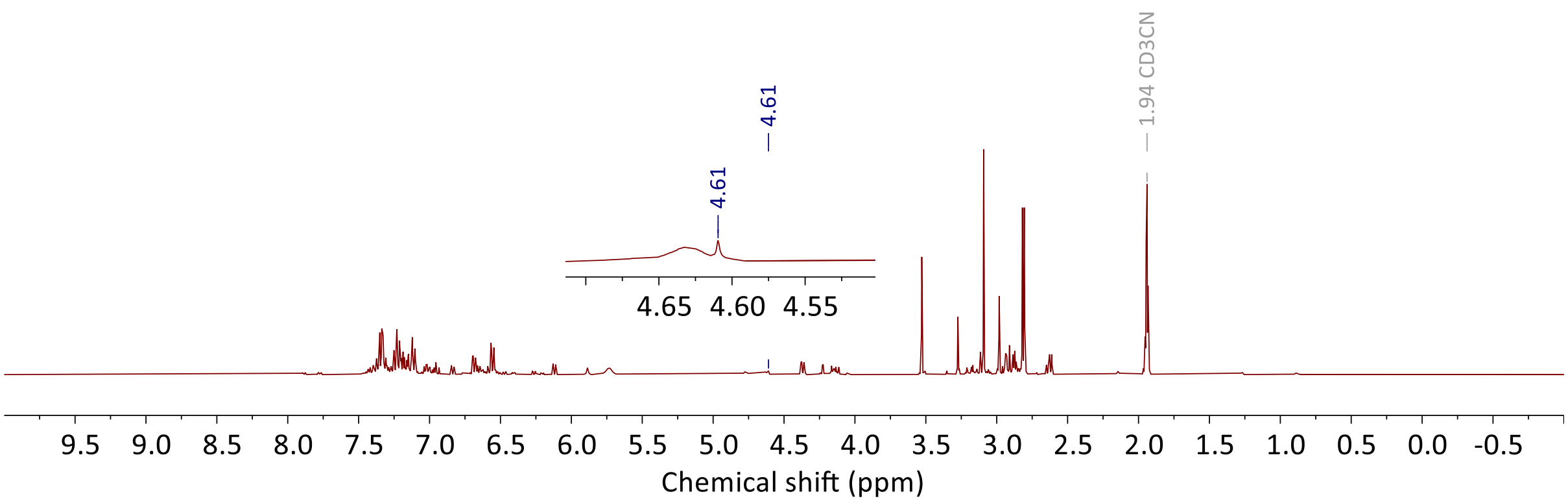

Figure S27: ^{1}H NMR spectrum (CD_3CN) of **2** after irradiation for one hour with 390 nm light and leaving for 3 days.

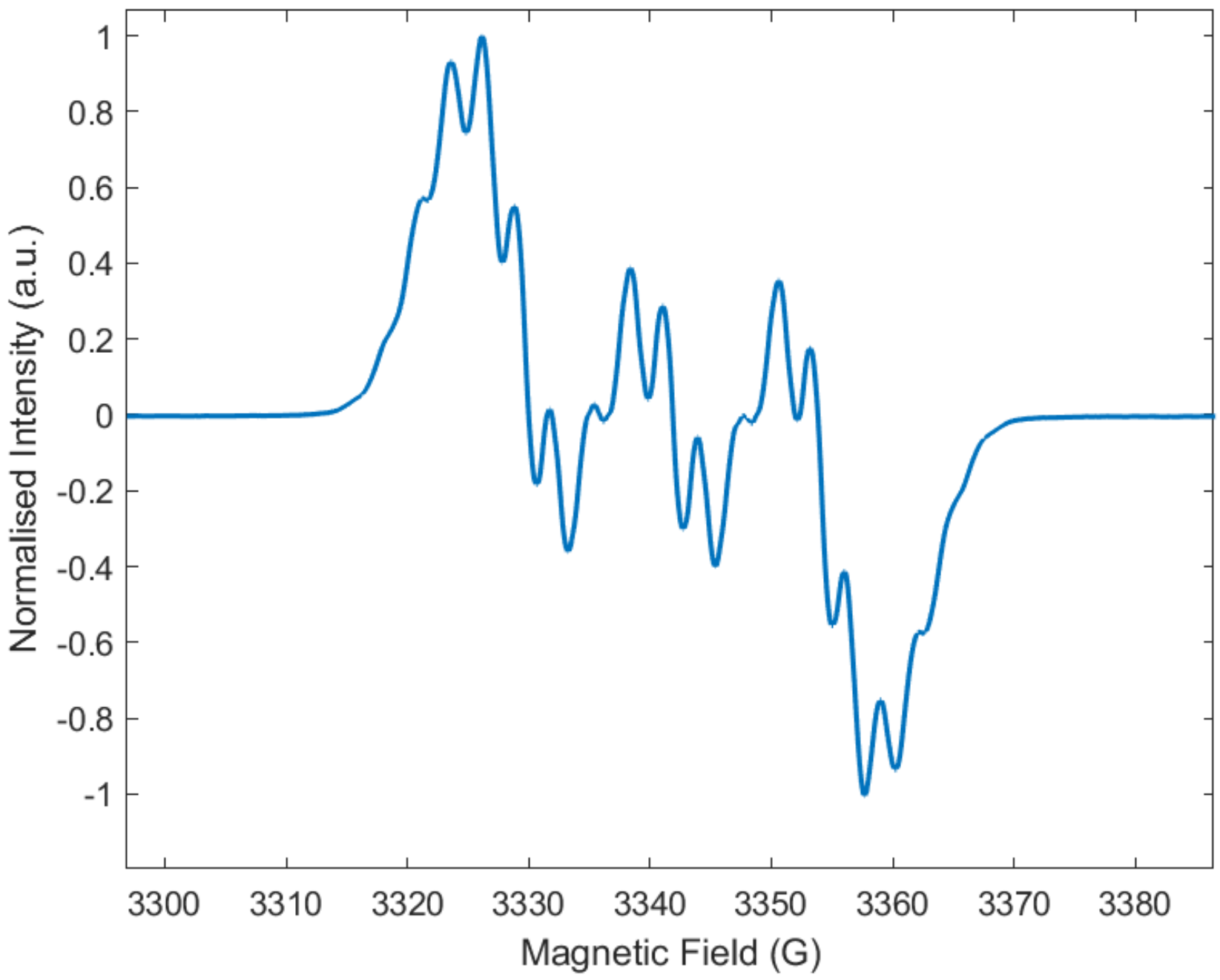

Figure S28: EPR spectrum of **2** after irradiation for one hour with 390 nm light, consistent with the formation of **3**$^{\bullet}$. Parameters: solvent = CD_3CN; frequency = 9.371 GHz; modulation amplitude = 0.5 G; scans = 10; temperature = 298 K.

3.2 Experimental studies on formation of 4

The first step in the catalytic cycle was probed by reacting (^{15}N)-**2** with N_2O and HBpin independently. No reaction was observed when (^{15}N)-**2** was reacted with N_2O under 390 nm irradiation by ^{1}H or ^{15}N NMR spectroscopy, and the EPR signal corresponding to **3**$^{\bullet}$ persisted. The reactivity observed between (^{15}N)-**2** and HBpin is detailed below.

3.2.1 Stoichiometric reactivity of ^{15}N-2 with DBpin

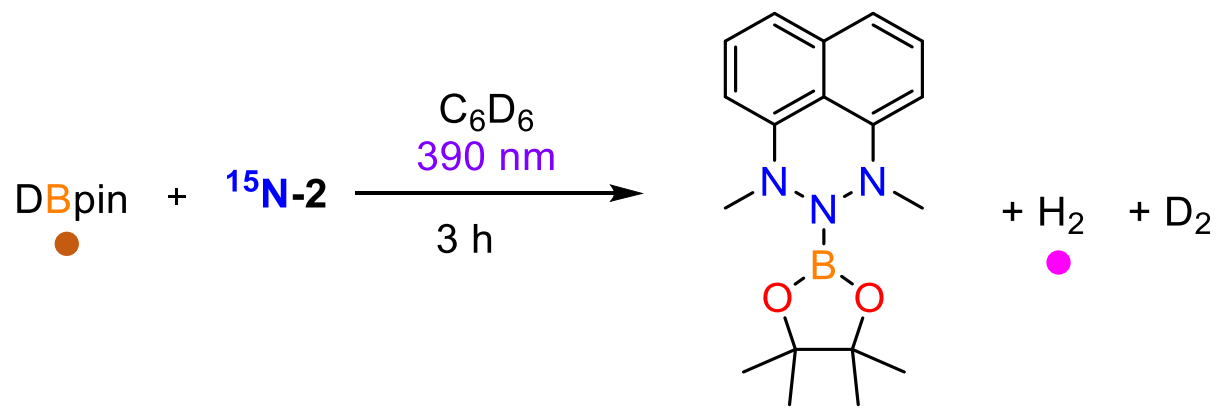

4

To a J Young NMR tube, a solution of ^{15}N-**2** (11 mg, 0.057 mmol, 1 equiv.), DBpin (from a 1 x 10^{-4} mmol/µL stock solution in C_6D_6; 407 µL, 0.057 mmol, 1.0 equiv.) and DMF (0.1 mL) were added. The reaction mixture was allowed to react for 3 hours under 390 nm irradiation before analysing by ^{1}H and ^{11}B{^{1}H} NMR spectroscopy, which showed no HD production. A doublet with $^1J_{N\text{-}B}$ coupling of ~35 Hz was observed in the ^{11}B{^{1}H} NMR spectrum for **4** in line with ^{15}N being spin active.

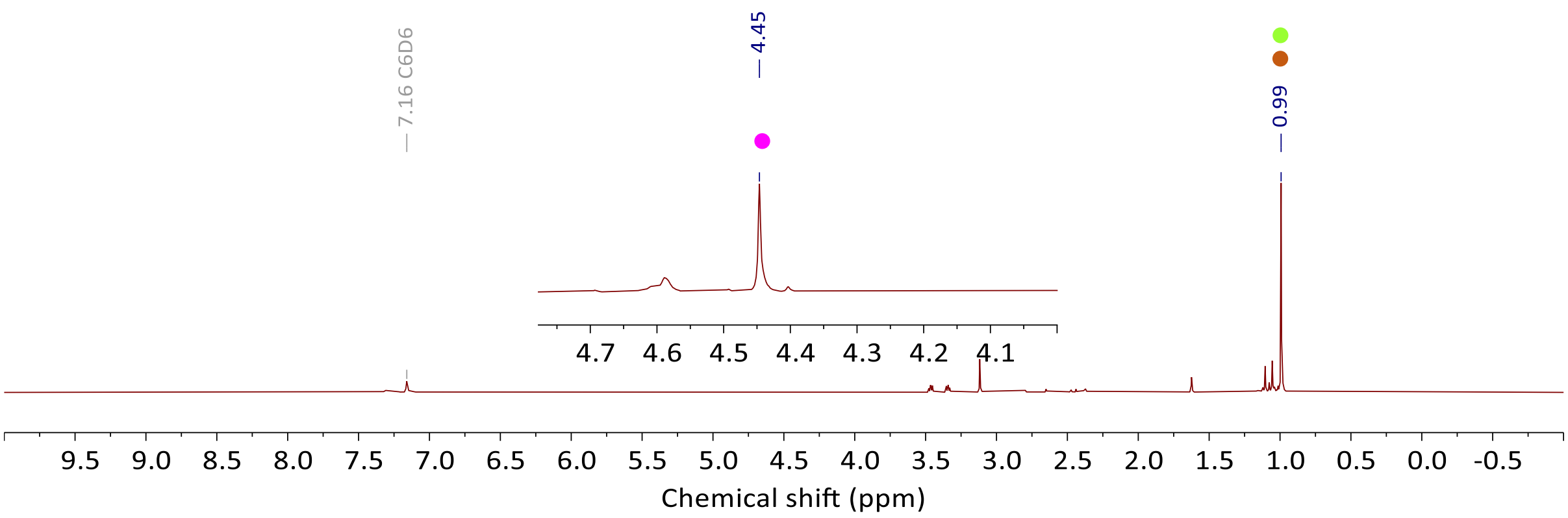

Figure S29: ^{1}H NMR spectrum (C_6D_6) of reaction 3.2.1 with an inset showing no HD formation.

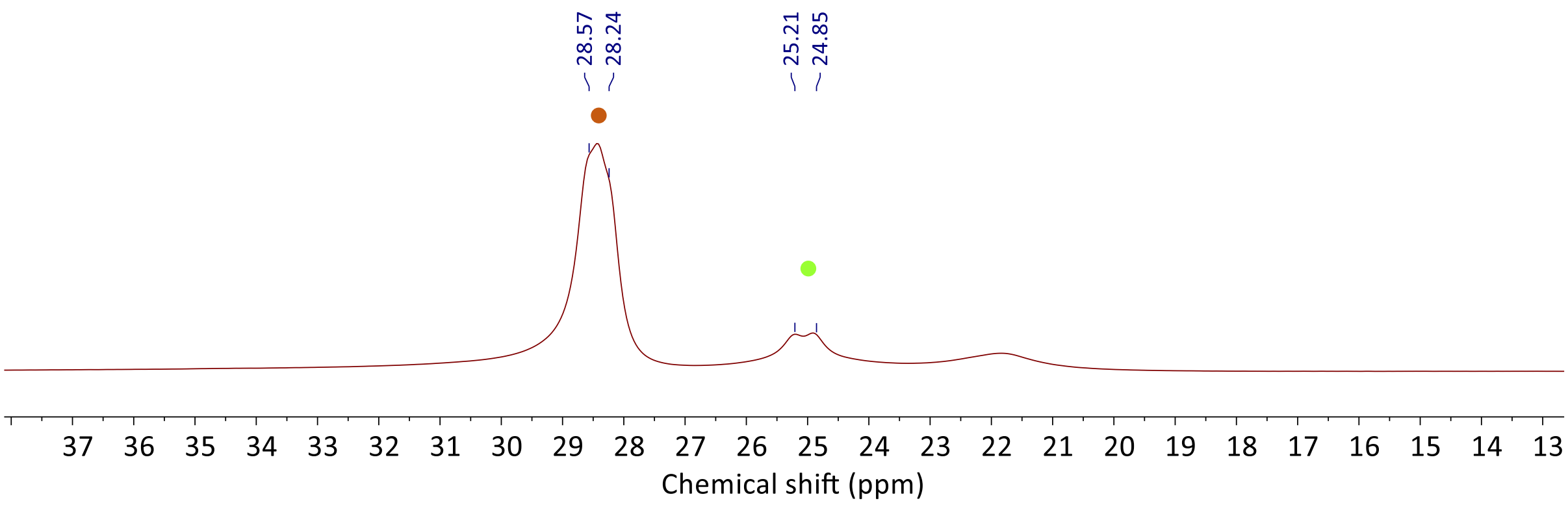

Figure S30: $^{11}B\{^1H\}$ NMR spectrum (C_6D_6) of reaction 3.2.1.

3.2.2 Stoichiometric reactivity of ^{15}N-2 with HBpin

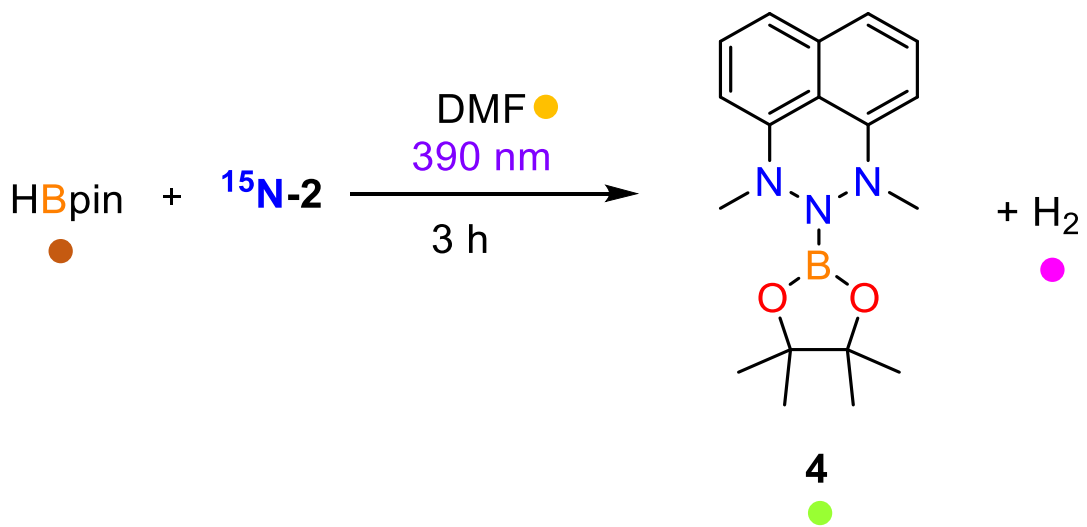

In a J Young NMR tube, ^{15}N-**2** was mixed with HBpin in DMF solvent and irradiated for 3 hours under 390 nm light. The product was analysed by $^{11}B\{^1H\}$ and ^{15}N NMR spectroscopy as well as ESI mass spectrometry.

Note: When conducted in the absence of 390 nm irradiation, no reaction was observed. Pre-irradiation of **2** to form **3**$^{\bullet}$ followed by reactivity with HBpin in the absence of irradiation also shows no reactivity.

Mass Spectrometry:

[(^{15}N-**4)**+H]$^+$ Calculated: 327.2010 Found: 327.2004

[(^{15}N-**4)**-H]$^-$ Calculated: 325.1854 Found: 325.1856

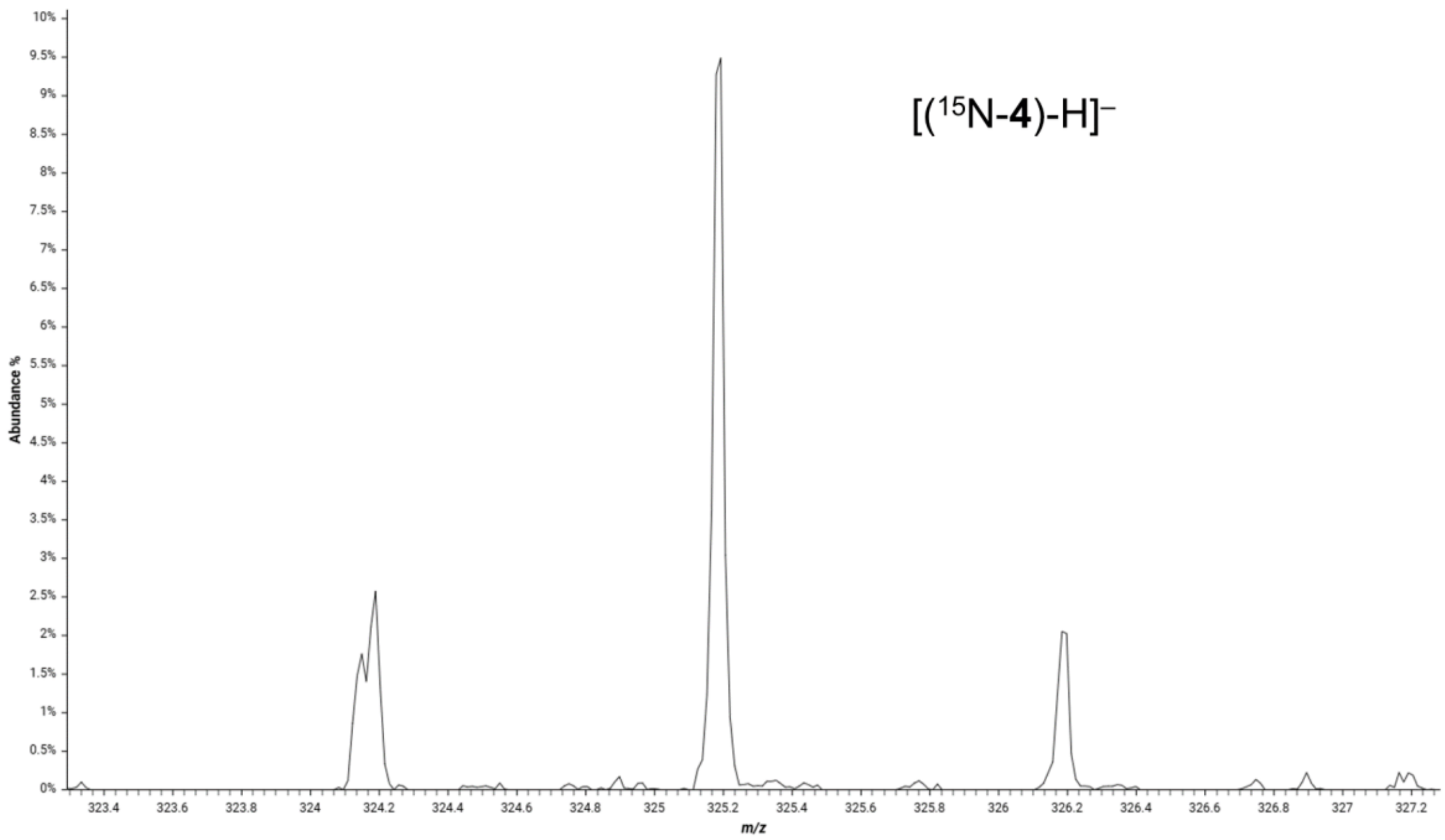

Figure S31: Mass spectrometry of ^{15}N-**4** in negative mode ([(^{15}N-**4)**-H]$^{-}$)**.**

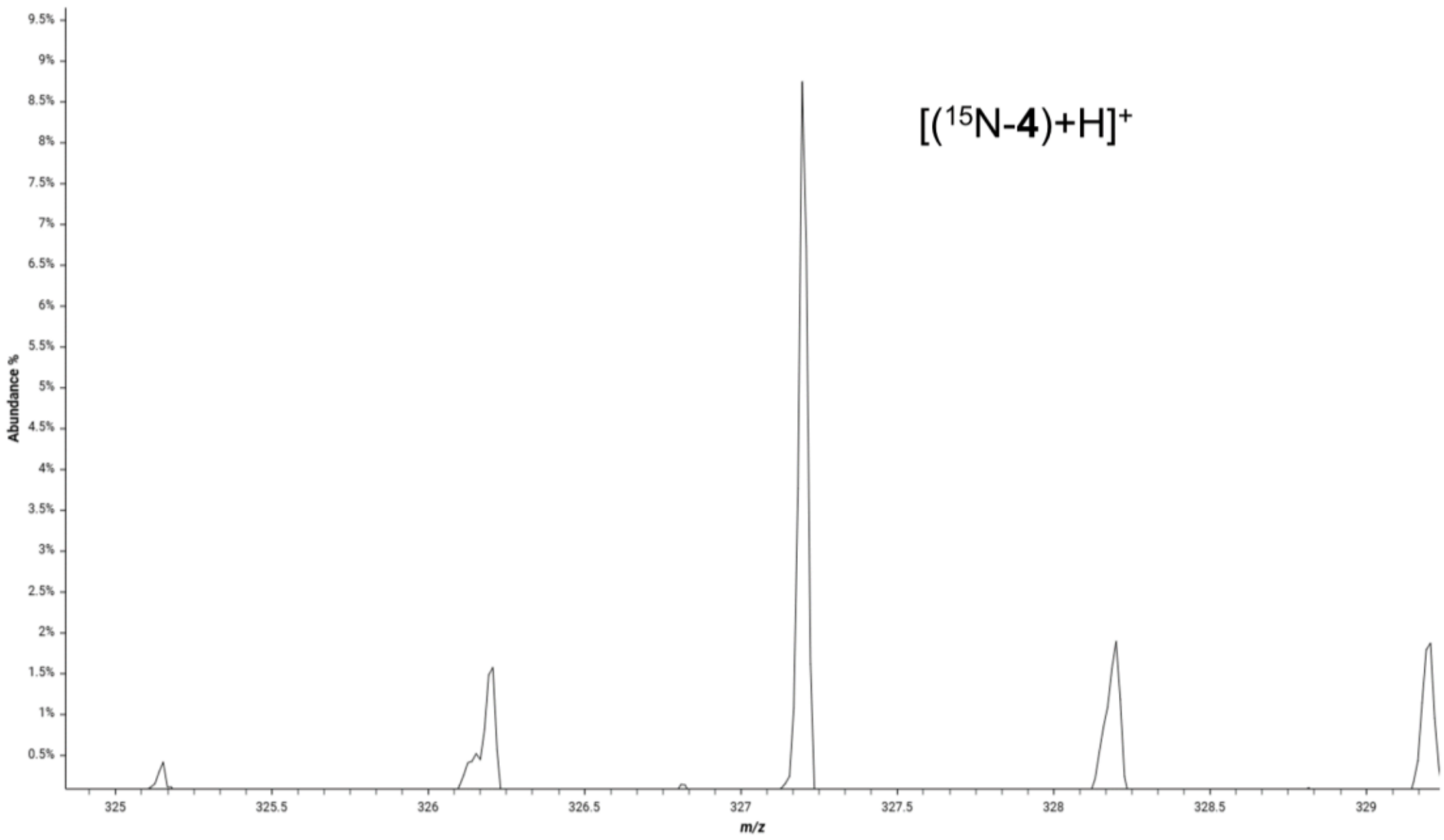

Figure S32: Mass spectrometry of ^{15}N-**4** in positive mode (left; [(^{15}N-**4)**+H]$^{+}$)

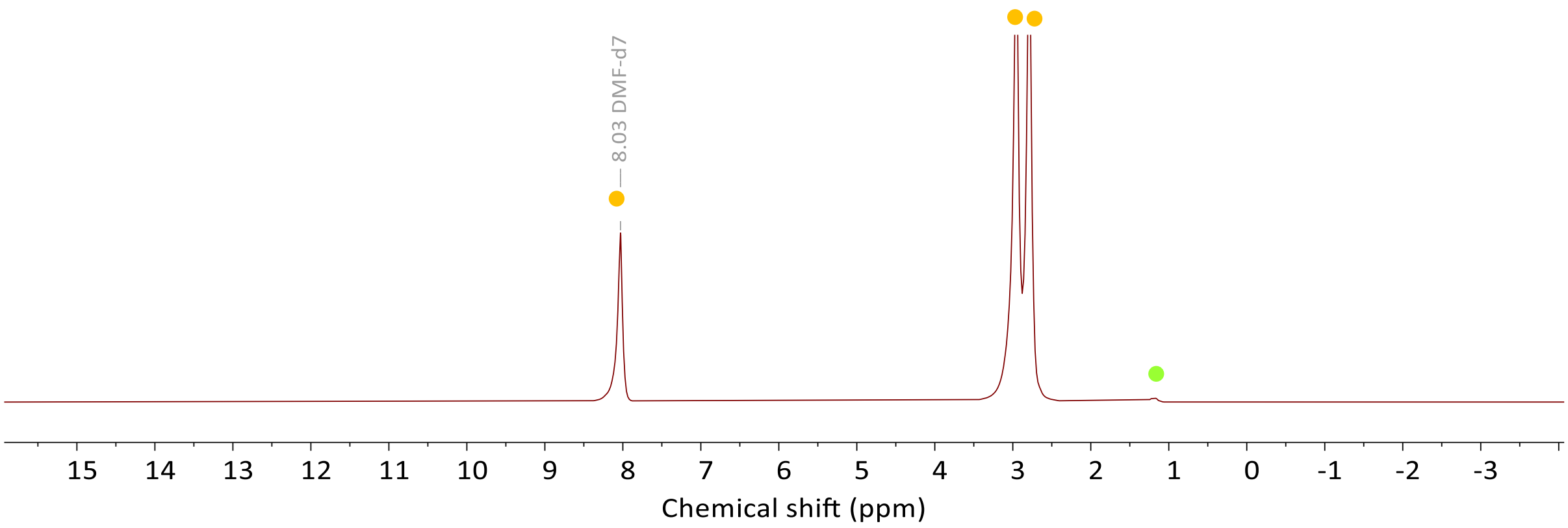

Figure S33: ^{1}H NMR spectrum (DMF) of reaction 3.2.2.

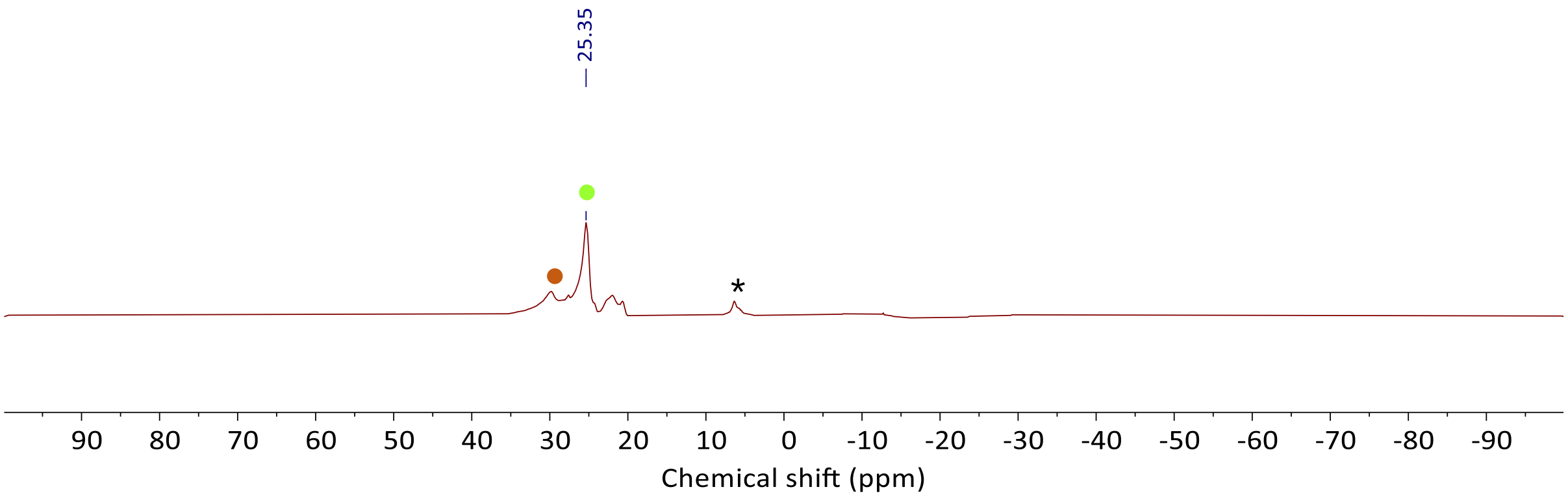

Figure S34: $^{11}B\{^{1}H\}$ NMR spectrum (DMF) of reaction 3.2.2. * denotes decomposition not present in the catalytic runs. Small resonances corresponding to HOBpin and $O(Bpin)_2$ are present due to trace water in the reaction mixture.

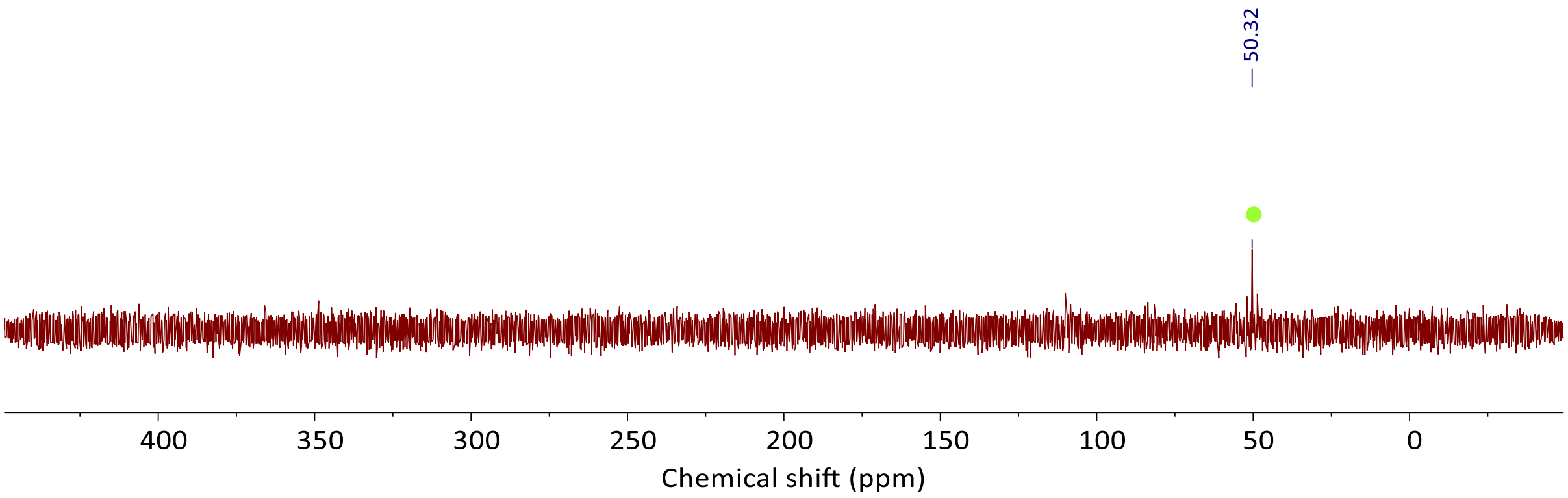

Figure S35: ^{15}N NMR spectrum (DMF) of reaction 3.2.2.

3.3 Studies on the reaction of 4 with N_2O

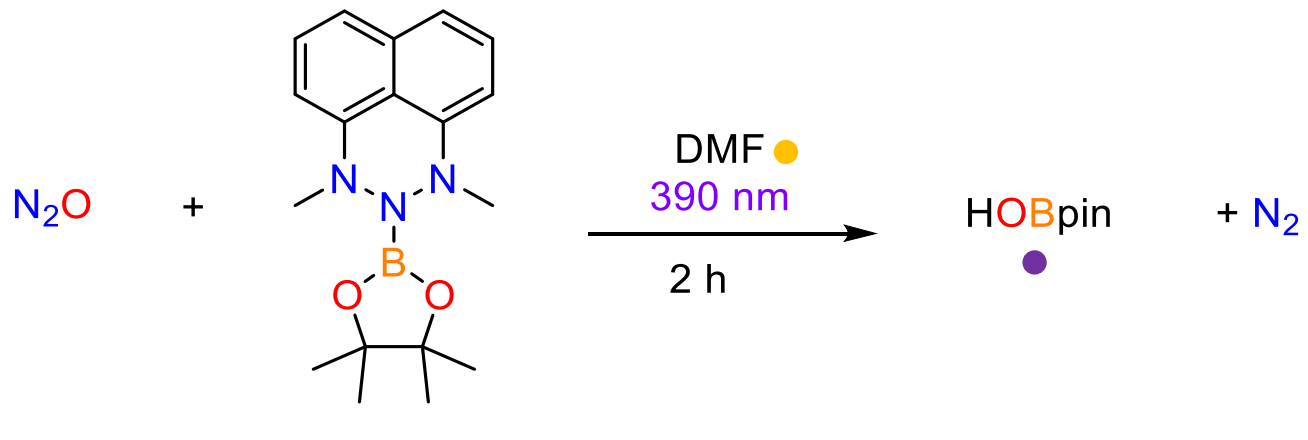

4

In a J Young NMR tube, *in situ* generated **4** (see section 3.2.2) in DMF solvent was frozen at 77 K. While frozen, the headspace of the NMR tube was evacuated. N_2O was then condensed into the NMR tube resulting in approximately 1 atm pressure at room temperature. After thawing, the reaction mixture was allowed to react for 2 hours under 390 nm irradiation. The reaction was analysed by ^{1}H and $^{11}B\{^{1}H\}$ NMR spectroscopy, confirming formation of HOBpin.

Note: No reaction was observed when the reaction was repeated in the absence of irradiation.

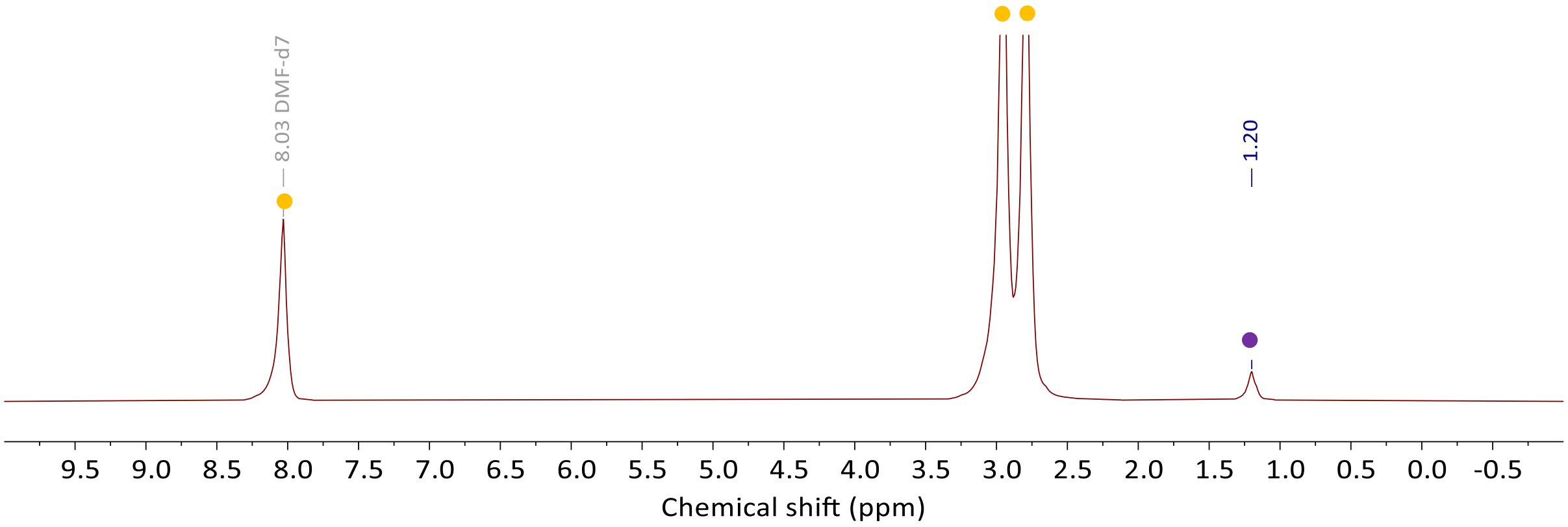

Figure S36: ^{1}H NMR spectrum (DMF) of reaction 3.3.

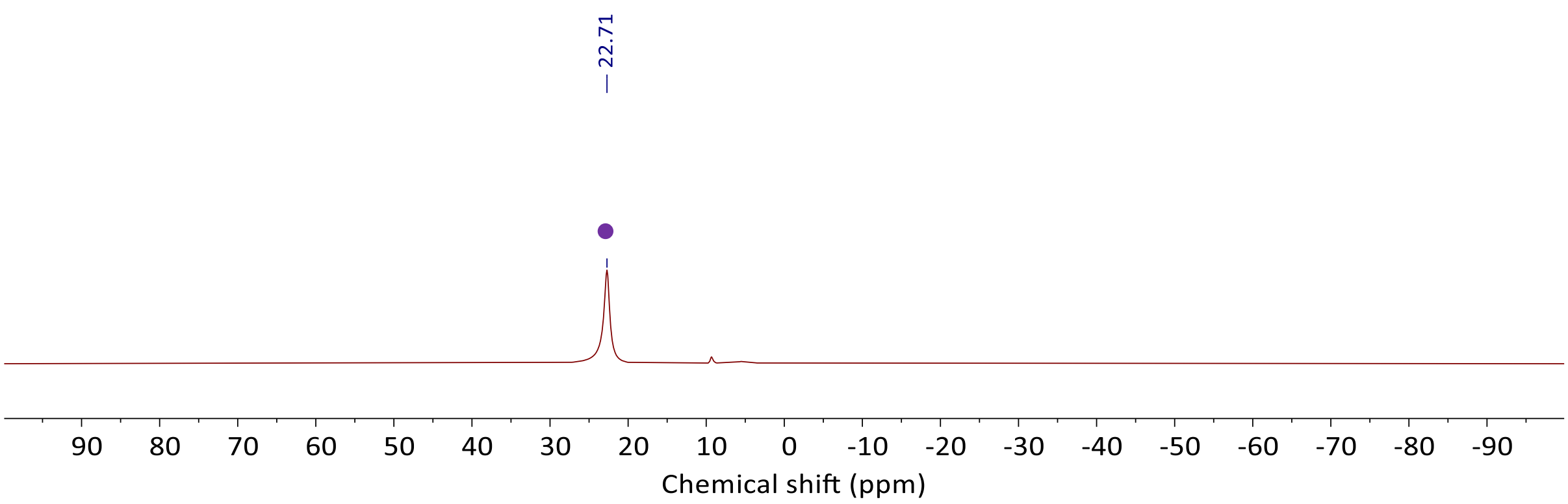

Figure S37: $^{11}B\{^{1}H\}$ NMR spectrum (DMF) of reaction 3.3.

4. Computational Mechanistic Studies

4.1 Method Benchmarking

Prior to mechanistic investigation, a benchmarking study was performed to identify an appropriate computational protocol for both ground-state and excited-state calculations. As the proposed catalytic cycle involves photoactivation of the pre-catalyst **2**, accurate descriptions of both the molecular structure and excited-state manifold were required.

4.1.1 Structural Benchmarking

The experimentally determined structure of **2** was used as a benchmark for evaluating the performance of several density functionals spanning multiple rungs of Jacob's ladder. Agreement between calculated and experimental structures was assessed using both all-atom and heavy-atom root-mean-square deviations (RMSDs). Heavy-atom RMSDs were considered particularly informative as they are less sensitive to hydrogen-atom positions and therefore provide a more robust measure of the underlying molecular framework.

Table S14: Structural benchmarking of selected density functionals against the experimentally determined structure of **2**.[23]

Method	RMSD (all atoms) / Å	RMSD (heavy atoms) / Å
ωB97X-D4	0.4594	0.0339
r^2SCAN-3c	0.4610	0.0347
B3LYP-D4	0.4606	0.0464
B97-3c	0.4604	0.0524
PBE	0.4677	0.0640

Among the methods examined, ωB97X-D4 provided the closest agreement with the experimentally determined heavy-atom framework. Although more computationally demanding than the composite methods investigated, the improved structural accuracy and demonstrated performance for main-group systems justified its use for all geometry optimisations and thermochemical calculations reported herein.

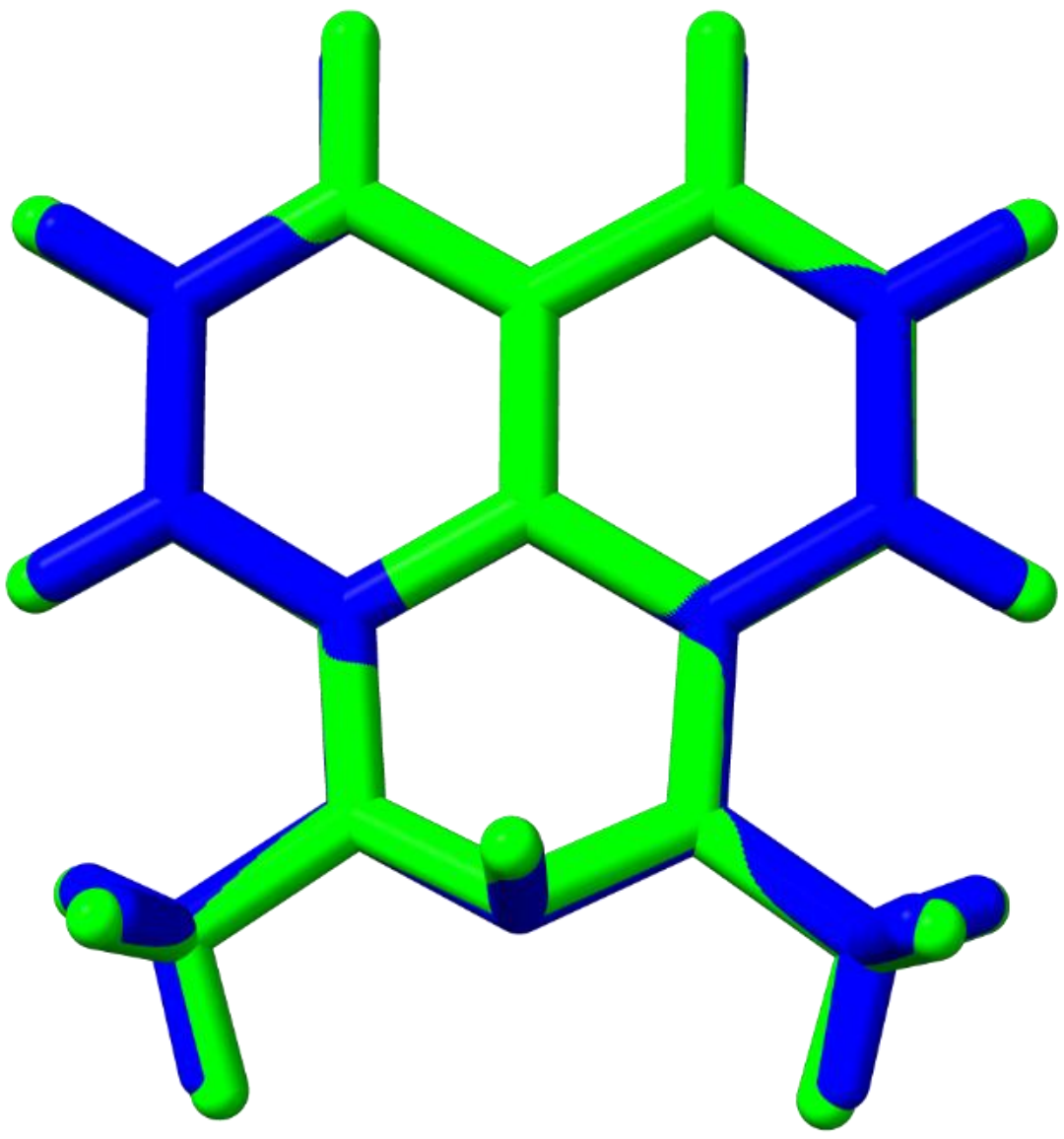

Figure S38: Overlay of the experimentally determined structure of **2** and the ωB97X-D4 optimised geometry. Crystal structure in blue; calculated structure in green.

4.1.2 Excited-State Benchmarking

As the catalytic reduction of N_2O proceeds only under irradiation, accurate description of the photoactive excited state is essential. Time-dependent density functional theory (TD-DFT) calculations were therefore used to benchmark **2** against the experimentally observed absorption feature centred near 350 nm.

Table S15. Calculated S_1 excitation wavelengths obtained using TD-DFT.

Functional	S1 wavelength (nm)
B3LYP	322.1
wB2PLYP	288.2
SCS/SOS-wB2PLYP	294.5
BP86	353.8
SCS-wPBEPP86	307.1
SCS-wB2GP-PLYP	299.2
SOS-RSX-QIDH	306.4
SCS/SOS-B2PLYP21	289.5
B97M-D4	326.7
PBE	353.8
M06-L	332.2
PBE0	314.9
r^2SCAN	332.5
SCAN	332.5
revPBE	352.6
RPBE	352.4
B2PLYP	311.8
ωB97X-D4	287.2

The experimentally observed absorption maximum was reproduced most accurately by BP86, PBE, revPBE and RPBE. Among these candidates, revPBE was selected for subsequent excited-state calculations owing to its excellent agreement with experiment (mirroring both the major absorption and the slight shoulder on the left side of the absorbance band) and its balanced description of the excited-state manifold. B97-3c was also found to reproduce the experimental excitation energy reasonably well (347.5 nm) and was therefore employed for preliminary exploration of excited-state reaction pathways, where the significantly reduced computational cost enabled efficient screening of multiple excited-state surfaces.

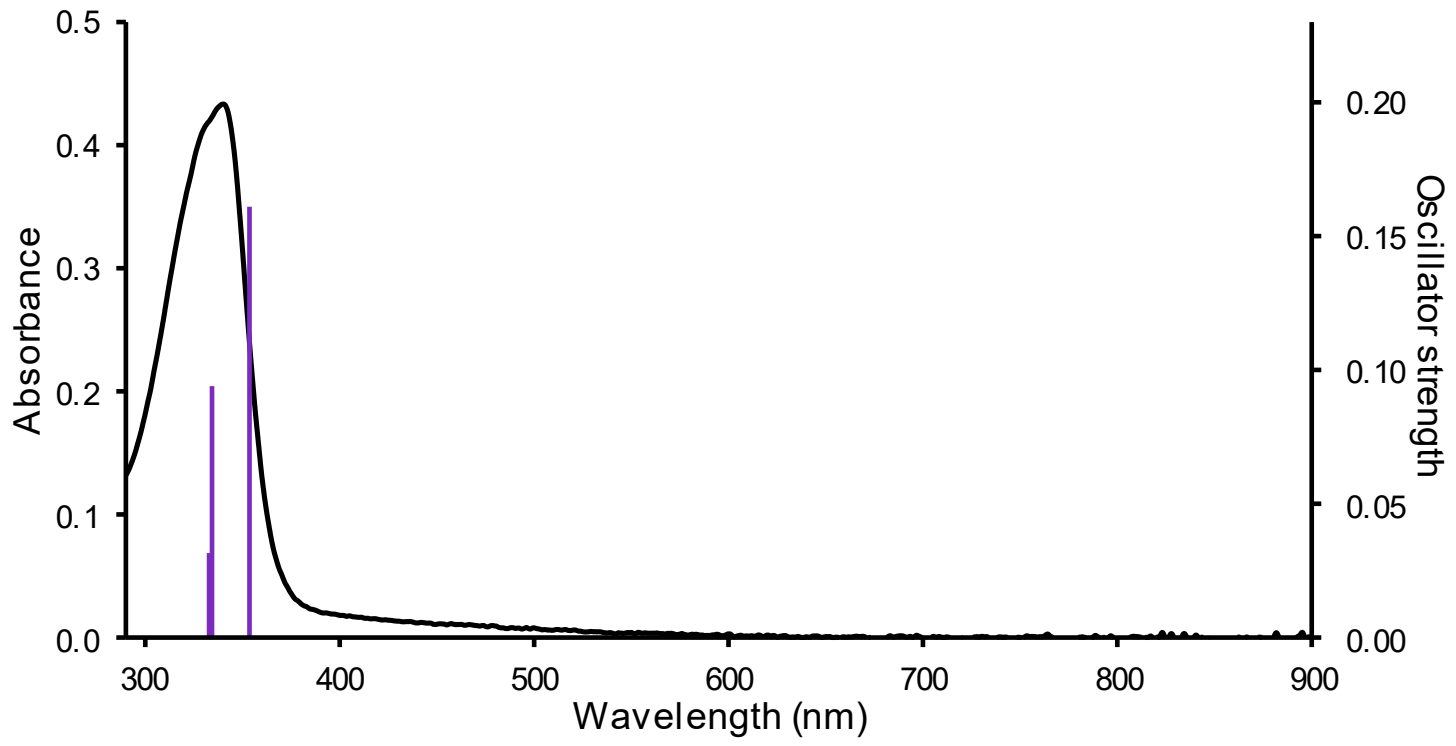

Figure S39: Comparison of experimental and calculated absorption wavelengths for the chosen functional revPBE.

4.2 Photochemical Activation of 2

The catalytic reduction of N_2O is observed experimentally only under irradiation, indicating that photoactivation of **2** is required for catalytic turnover. Experimental evidence for the formation of **3˙** suggests that N–H bond cleavage in **2** occurs before or during the catalytic cycle. Accordingly, the initial stages of the mechanism were investigated to determine how photoexcitation promotes N–H bond activation in **2** and generates the experimentally observed radical intermediate, **3˙**.

4.2.1 Exploration of the Singlet Excited-State Manifold

As the experimentally observed absorption band corresponds to excitation into S_1, initial investigations focused on N–H bond activation on the singlet manifold of **2**. Exploration of the S_1–S_4 surfaces revealed that N–H bond cleavage remains energetically unfavourable and does not occur spontaneously following excitation (Figure S40). Direct reaction on the singlet manifold is therefore unlikely to account for the experimentally observed catalytic activity. Furthermore, the short lifetimes typically associated with excited singlet states make the involvement of long-lived singlet intermediates improbable.

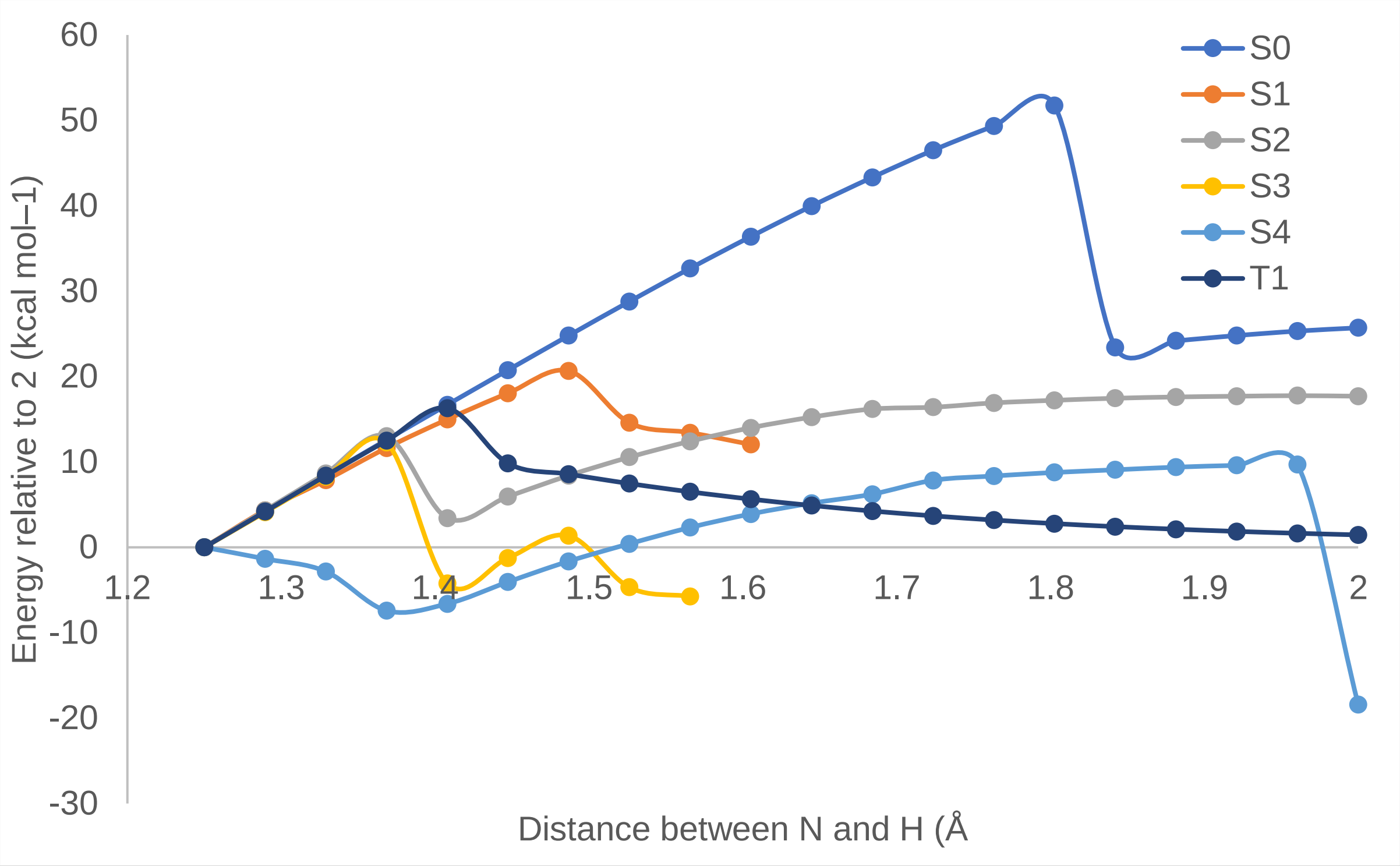

Figure S40: Relaxed N–H bond scan of **2** on the $S_{0\text{-}4}$ and T_1 surfaces (B97-3c).

4.2.2 Population of the Triplet Manifold

Inspection of the excited-state spectrum of **2** revealed several triplet states lying energetically close to the photoactive S_1 state. The small singlet–triplet energy separations suggest that intersystem crossing (ISC) may provide an efficient pathway for population of the triplet manifold following photoexcitation.

Table S16: Comparison of the low-lying excited states obtained using TD-DFT and CASSCF/CASPT2.

State	TD-DFT (eV)	CASSCF/CASPT2 (eV)
T1	2.593	2.574
T2	3.171	3.277
T3	3.330	3.441
S1	3.516	3.367

Multireference CASSCF/CASPT2 calculations on **2** reproduce the low-lying excited-state manifold predicted by TD-DFT and confirm the energetic proximity of the triplet states to S_1. The close agreement between the two methods provides confidence in the electronic structure description used for the subsequent mechanistic investigation. The accessibility and persistence of the triplet manifold were further evaluated through spin–orbit coupling and ISC rate calculations.

Table S17: Calculated spin–orbit coupling matrix elements and rate constants accounting for the Herzberg-Teller effect for population and decay of the triplet manifold.

Transition	SOC (cm^{-1})	Rate constant (s^{-1})
S1 → T1	0.02849	3.024374×10^7
S1 → T2	0.28589	5.418896×10^6
S1 → T3	0.28602	6.035772×10^6
SUM	0.45209	4.169841×10^7
T1 → S0	0.08058	1.674063×10^6

The calculated spin–orbit couplings are small; however, Herzberg–Teller vibronic coupling enables efficient intersystem crossing from the photoexcited singlet state into the triplet manifold.[24] The combined ISC rate is calculated to be 4.17×10^7 s^{-1}, indicating that triplet-state population is kinetically accessible following photoexcitation. Relaxation of the lowest triplet state to the ground state is predicted to occur more slowly (k(T1→S0) = 1.67×10^6 s^{-1}), corresponding to an estimated lifetime of approximately 0.6 μs. This transient triplet-state population provides a sufficient time window for N–H bond cleavage and is therefore consistent with a triplet-mediated activation pathway.[25]

4.2.3 Triplet-State N–H Activation

Following population of the triplet manifold of **2**, N–H bond activation was investigated. In contrast to the singlet-state surfaces, hydrogen atom loss from triplet **2** proceeds via a readily accessible transition state with a calculated free-energy barrier of 7.98 kcal mol^{-1}, yielding **3˙**. In addition to direct N–H homolysis, reaction between triplet **2** and singlet **2** was examined. This pathway proceeds via a transition state with a calculated free-energy barrier of 6.27 kcal mol^{-1} and likewise generates **3˙**. Despite the direct homolytic cleavage of the N–H bond being slightly higher in energy than the bimolecular route, it is suggested to be the major pathway due to the statistical improbability of a triplet-state **2** and a singlet state **2** colliding, as well as the predicted sufficient lifetime of the triplet state for homolytic cleavage. Both pathways lead to formation of the experimentally observed **3˙** and are significantly more favourable than the corresponding singlet-state activation processes (55.51 kcal mol^{-1}).

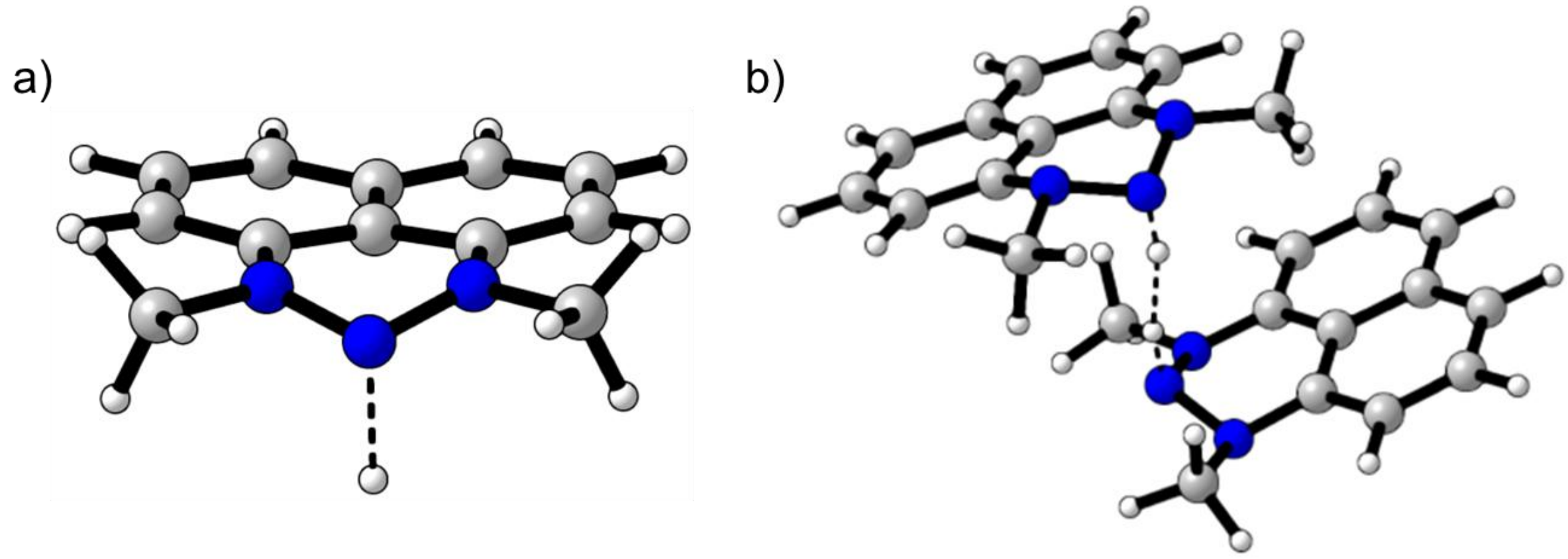

Figure S41: Calculated transition states for (a) direct hydrogen atom loss from triplet-state **2** and (b) reaction between triplet-state and singlet-state **2**.

4.3 Reactivity of 3$^{\bullet}$

Generation of **3$^{\bullet}$** provides a mechanistically plausible outcome of photoactivation and is consistent with the experimentally observed radical species. The reactivity of this intermediate towards HBpin was therefore investigated, in line with experimental observations.

4.3.1 Doublet-State Reactivity of 3$^{\bullet}$ Towards HBpin

The experimentally observed **3$^{\bullet}$** corresponds to a doublet electronic state. Relaxed potential energy surface scans performed along the N···B approach coordinate revealed no stable doublet-state adduct. Instead, the interaction was found to be predominantly repulsive, with the energy increasing continuously as HBpin approached the central nitrogen atom of **3$^{\bullet}$**. These results indicate that direct activation of HBpin by the experimentally observed doublet radical is not energetically favourable.

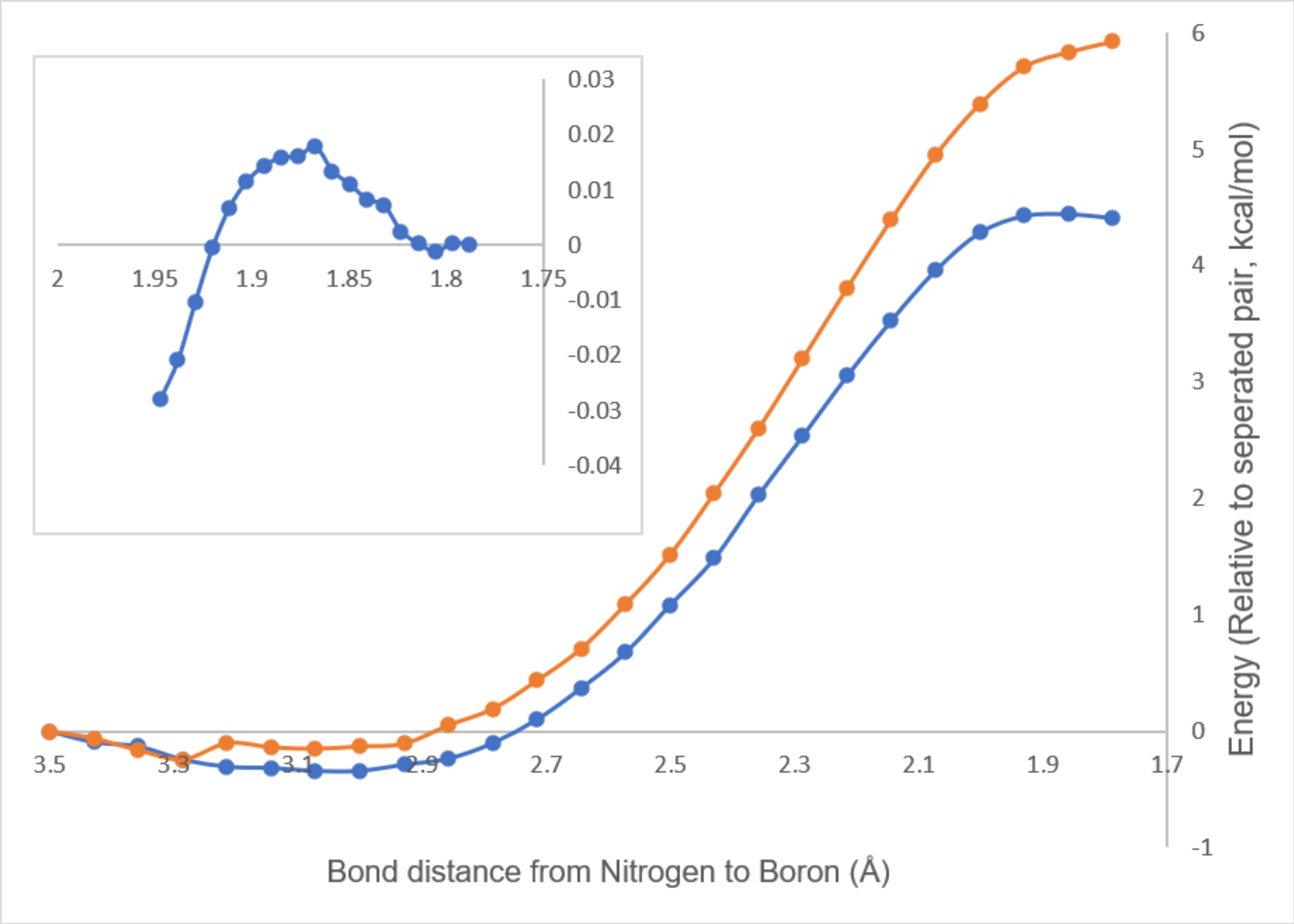

Figure S42: Relaxed potential energy surface scan describing the approach of HBpin to the doublet-state **3**˙ with an inset showing the surface maximum.

4.3.2 Accessible Higher Spin States

As direct activation of HBpin by the doublet **3**˙ was not observed, higher spin states were investigated. Both TD-DFT and CASSCF/CASPT2 calculations identify low-lying quartet states in close proximity to the doublet manifold (Table S18), suggesting that access to a reactive quartet state is feasible. Investigation of the region connecting the doublet and quartet manifolds further identified energetically accessible crossing points along the HBpin approach coordinate.

Explicit doublet–quartet intersystem crossing rate calculations were not performed, as the ESD(ISC) implementation available in ORCA is not currently applicable to doublet systems. CASSCF/NEVPT2 calculations indicate relatively small spin–orbit coupling matrix elements, suggesting that direct SOC-mediated spin crossing is unlikely to dominate. However, similar behaviour is observed for the singlet–triplet manifold, where population transfer is facilitated despite small SOC values. Given the experimentally observed requirement for irradiation, the energetic proximity of the doublet and quartet states, and the presence of accessible crossing regions, population of a reactive quartet state is proposed as the most plausible route to HBpin activation.

Table S18: Comparison of low-lying doublet and quartet states of **3•** obtained using TD-DFT and CASSCF/CASPT2.

State	TD-DFT (eV)	CASSCF/CASPT2 (eV)
D1	1.493	1.959
D2	1.788	1.991
D3	2.409	2.439
Q1	2.660	2.475
D4	3.329	2.636
Q2	3.129	2.666

Table S19: Calculated spin–orbit coupling matrix elements of **3•** using CASSCF/NEVPT2.

Transition	SOC (cm^{-1})
D4 → Q1	0.040012
D4 → Q2	0.031544
Q1 → D1	0.004243

4.3.3 Formation of Int-1• and 4

In contrast to the doublet surface, productive interaction between the quartet-state **3•** and HBpin yields a stable N–B bonded intermediate (**Int-1•**) *via* a transition state with a calculated free-energy barrier of 15.38 kcal mol^{-1}.

Following formation of **Int-1•**, the conversion to **4** was first investigated under thermal conditions. A transition state corresponding to B–H bond cleavage was successfully located; however, the calculated free-energy barrier of 34.97 kcal mol^{-1} indicates that this process is prohibitively high in energy and is therefore unlikely to occur under the experimental reaction conditions.

Given that catalysis proceeds only under continuous irradiation, a photochemical pathway was subsequently explored. Excitation of **Int-1•** populates the second quartet excited state from which B–H bond cleavage proceeds readily via a transition state with a calculated free-energy barrier of only 1.58 kcal mol^{-1}. Following bond cleavage, the system relaxes onto the quartet ground-state surface to afford **4**. However, it should be noted that the photodissociation step was calculated using TDDFT at the revPBE level because this method best reproduced the experimental excitation energies, whereas the remaining mechanism was calculated using wB97X. Applying revPBE to the entire mechanism would be inappropriate because it does not reproduce ground-state structures and transition-state energies as reliably as wB97X. Since the energies were obtained using different methods, they are therefore not directly comparable.

Table S20: Comparison of low-lying doublet and quartet states of **Int-1•** obtained using TD-DFT and CASSCF.

State	TD-DFT (eV)	CASSCF (eV)
Q1	0.986240	1.763
Q2	1.429685	2.594
Q3	1.681926	3.010
Q4	2.026582	3.437
Q5	2.207135	3.674
Q6	2.583005	4.022

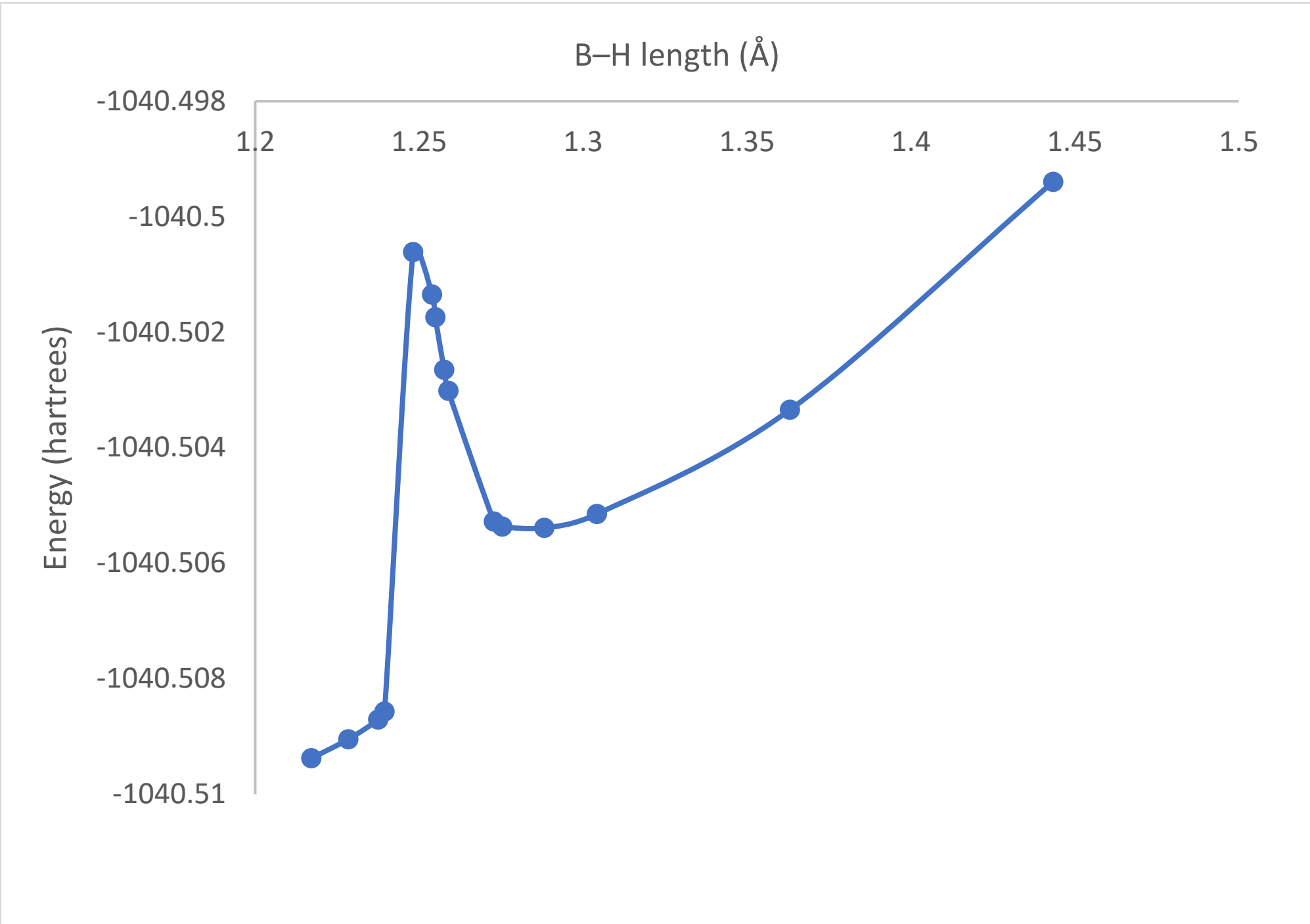

Figure S43: Relaxed potential energy surface scan describing the lengthening of the B–H bond in **Int-1**$^{\bullet}$

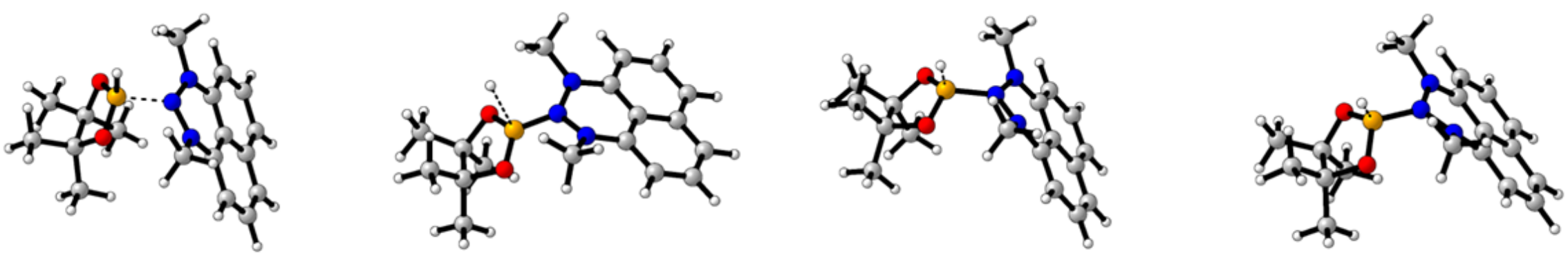

TS2 **High energy TS3** **Photo induced TS3** **Conical intersection**

Figure S44: Transition state for **3**$^{\bullet}$ reacting with HBpin (TS2) to form **Int-1**$^{\bullet}$ and for the cleavage of the B–H bond under thermal conditions (high energy **TS3**) and under irradiation (photo induced **TS3**) to form **4.** The conical intersection for **TS3** relaxing back to the ground state quartet is also shown.

4.4 N_2O Reduction and Product Formation

Having established a viable route to the **4**, the subsequent reduction of N_2O was investigated.

4.4.1 Activation of N_2O

Coordination of N_2O to **4** occurs readily, yielding a stable NB–N_2O (**Int-2**) adduct with a barrier of 2.13 kcal mol^{-1}. Subsequent concerted N–O bond cleavage, N–B bond cleavage, and N_2 extrusion proceed via a low-energy transition state (–0.3 kcal mol^{-1}) to generate a $^{•}$OBpin species and reform **3$^{•}$**. Although the free-energy profile appears effectively barrierless, inspection of the electronic energy surface confirms the presence of a genuine transition state connecting the NB–N_2O adduct and products.

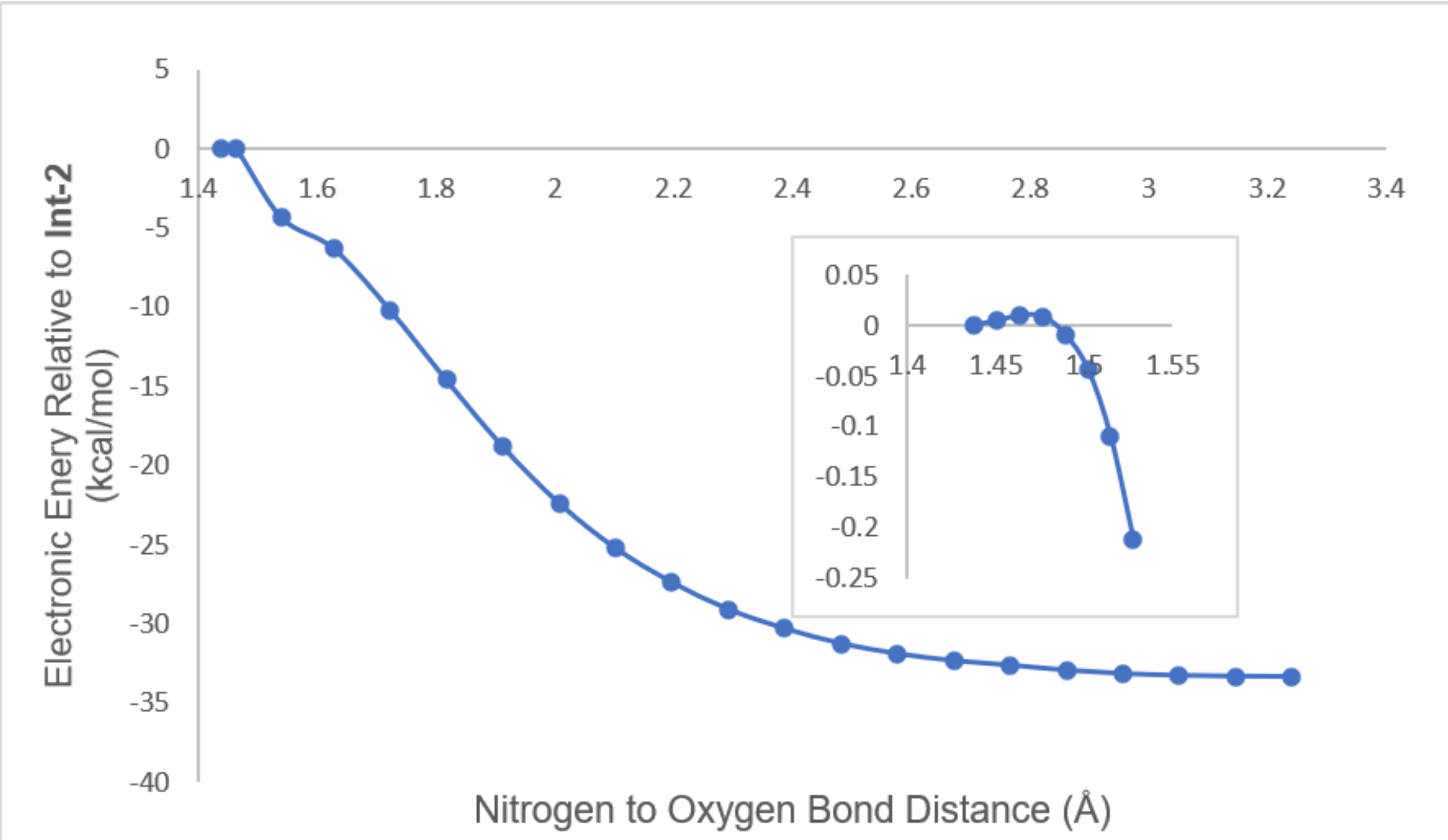

Figure S45: Reaction profile describing N_2 extrusion from Int-2 with an inset showing the small increase in electronic energy.

4.4.2 Intersystem Crossing Studies on 4

Although the photochemical pathway generates **4** initially in its reactive triplet state, the species can be readily characterised by NMR spectroscopy once irradiation is removed. This observation indicates that the triplet state relaxes efficiently to the diamagnetic singlet ground state following photoexcitation. To investigate this relaxation process, the excited-state manifold of **4** was examined using the same computational protocol employed previously for **2**.

TD-DFT and CASSCF/CASPT2 calculations reveal several low-lying singlet and triplet excited states that are closely spaced in energy (Table S21). As observed for **2**, the small singlet–

triplet energy separations suggest that efficient intersystem crossing provides a viable pathway for relaxation of the initially formed triplet state.

Table S21: Comparison of the low-lying excited states obtained using TD-DFT and CASSCF/CASPT2.

State	TD-DFT (eV)	CASSCF/CASPT2 (eV)
T1	2.550324	1.408
T2	3.093584	2.448
T3	3.133661	3.101
S1	3.444792	3.165

To quantify this process, spin–orbit coupling matrix elements and intersystem crossing rate constants accounting for Herzberg–Teller vibronic coupling were calculated (Table S22). The calculated ISC rate of $2.7925471 \times 10^6\ s^{-1}$ indicates that relaxation to the singlet ground state is kinetically accessible on the experimental timescale.

Table S22: Calculated spin–orbit coupling matrix elements and rate constants accounting for the Herzberg-Teller effect for population and decay of the triplet manifold.

Transition	SOC (cm^{-1})	Rate constant (s^{-1})
S1 → T1	0.18789	4.4902898×10^7
S1 → T2	0.17108	3.360502×10^5
SUM	0.45209	3.00980817×10^7
T1 → S0	0.26376	2.7925471×10^6

4.4.3 Formation of $O(Bpin)_2$

The formed $^{\bullet}$OBpin subsequently reacts with HBpin to afford the experimentally observed $O(Bpin)_2$ product in a concerted process with a barrier of 12.77 kcal mol^{-1}.

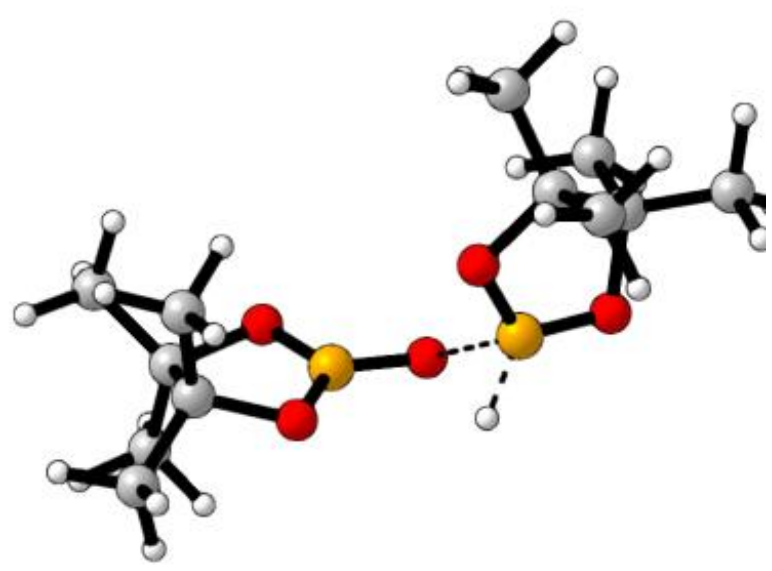

Figure S46: Transition state for the reaction of $^{\bullet}$OBpin with HBpin (TS6).

4.5 CASSCF

4.5.1 CASSCF of 2

Table S23: Low-lying singlet states of **2** obtained from state-averaged CASSCF/CASPT2 calculations.

State	CASPT2 Energy (eV)	Dominant Configuration(s)	Weight(s)
S_0	0.000	22220000	0.870
S_1	3.367	22210100 + 22121000	0.509 + 0.256
S_2	3.729	22211000	0.799
S_3	4.689	21220100 + 22202000	0.260 + 0.143
S_4	4.028	21221000 + 22210010	0.423 + 0.224
S_5	5.204	21220100 + 12221000	0.228 + 0.116
S_6	4.496	22210010 + 22121000	0.344 + 0.294
S_7	4.873	22121000 + 22210100	0.213 + 0.203
S_8	5.158	22120100	0.624
S_9	5.801	22202000 + 12221000	0.278 + 0.120

Table S24: Low-lying triplet states of **2** obtained from state-averaged CASSCF/CASPT2 calculations.

State	CASPT2 Energy (eV)	Dominant Configuration(s)	Weight(s)
T_1	2.574	22211000	0.729
T_2	3.277	22210100 + 22121000	0.637 + 0.138
T_3	3.441	22121000	0.707
T_4	4.056	212211000 + 222110010	0.443 + 0.309
T_5	4.471	221211000 + 222110100	0.637 + 0.179
T_6	5.916	122211000 + 222110001	0.237 + 0.226
T_7	5.050	122210100 + 221210001	0.375 + 0.151
T_8	5.232	212210100 + 221111100	0.363 + 0.176
T_9	5.346	222101100 + 221111100	0.247 + 0.144
T_{10}	6.506	221111100 + 222101100	0.325 + 0.180

Table S25: Density matrix for **2** obtained from state-averaged CASSCF/CASPT2 calculations.

	**0**	**1**	**2**	**3**	**4**	**5**	**6**	**7**
**0**	1.8578	0	0	0	0	0	0	0
**1**	0	1.7233	0	0	0	0	0	0
**2**	0	0	1.6660	0	0	0	0	0
**3**	0	0	0	1.4758	0	0	0	0
**4**	0	0	0	0	0.5331	0	0	0
**5**	0	0	0	0	0	0.4254	0	0
**6**	0	0	0	0	0	0	0.2192	0
**7**	0	0	0	0	0	0	0	0.0992

Table S26: Spin density matrix for **2** obtained from state-averaged CASSCF/CASPT2 calculations.

	**0**	**1**	**2**	**3**	**4**	**5**	**6**	**7**
**0**	0.0627	0.0000	0.0000	-0.0029	0.0013	0.0000	0.0000	0.0006
**1**	0.0000	0.1217	0.0150	0.0000	0.0000	0.0020	-0.0023	0.0000
**2**	0.0000	0.0150	0.1403	0.0000	0.0000	-0.0011	0.0014	0.0000
**3**	-0.0029	0.0000	0.0000	0.1704	0.0006	0.0000	0.0000	-0.0005
**4**	0.0013	0.0000	0.0000	0.0006	0.1849	0.0000	0.0000	0.0011
**5**	0.0000	0.0020	-0.0011	0.0000	0.0000	0.1785	-0.0020	0.0000
**6**	0.0000	-0.0023	0.0014	0.0000	0.0000	-0.0020	0.1021	0.0000
**7**	0.0006	0.0000	0.0000	-0.0005	0.0011	0.0000	0.0000	0.0395

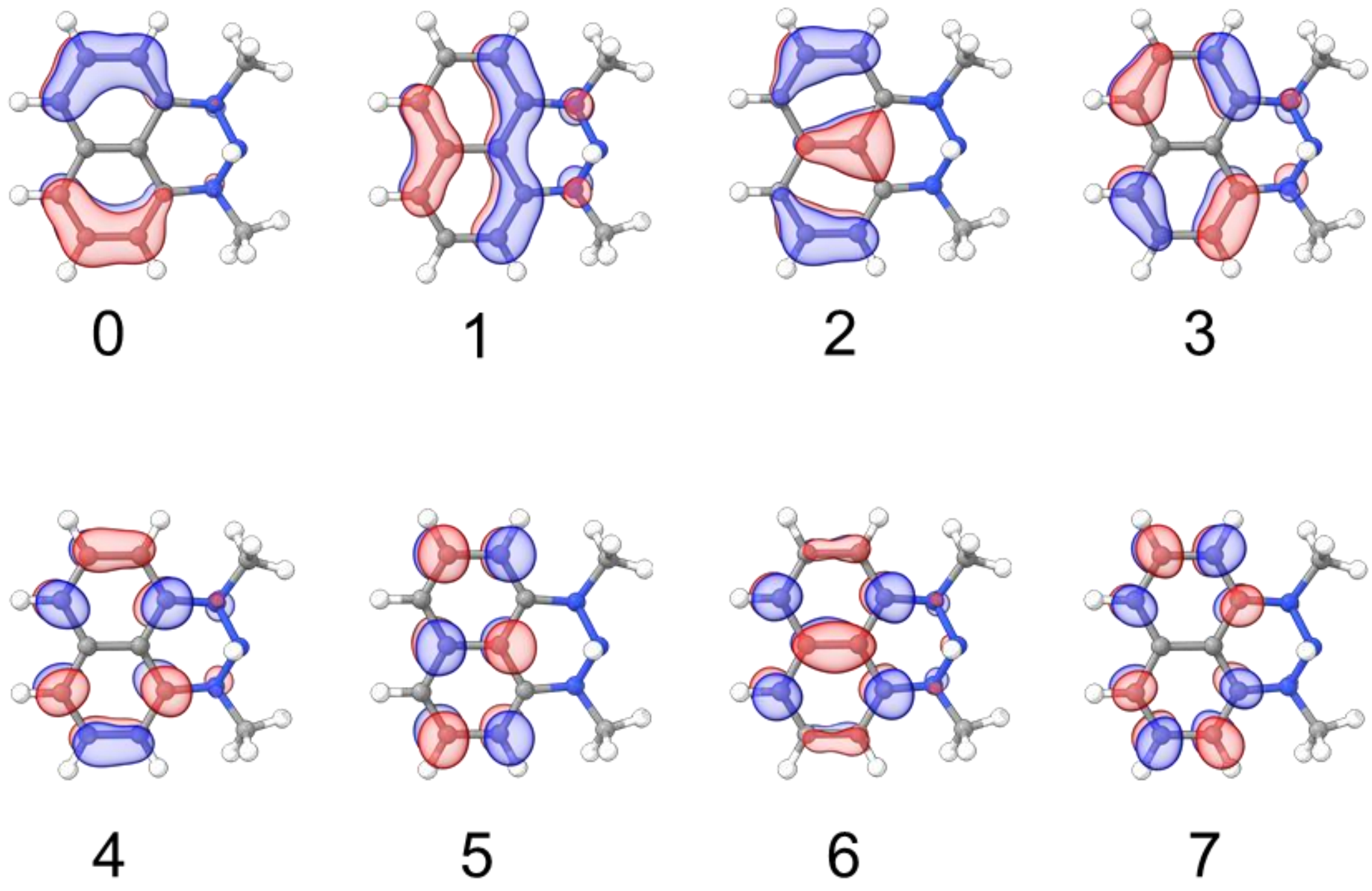

Figure S47: CASSCF orbitals of **2** (isovalue = 0.05).

4.5.2 CASSCF of 3˙

Table S27: Low-lying doublet states of **3˙** obtained from state-averaged CASSCF/CASPT2 calculations.

State	CASPT2 Energy (eV)	Dominant Configuration(s)	Weight(s)
D_1	0.000	222210000	0.851
D_2	2.636	222111000	0.664
D_3	2.439	222201000 + 212211000	0.428 + 0.151
D_4	3.153	222200100 + 212210100	0.493 + 0.157
D_5	3.424	222110100 + 221211000	0.626 + 0.133
D_6	3.502	222110100 + 221211000	0.503 + 0.213
D_7	3.953	222120000 + 222201000	0.377 + 0.224
D_8	4.302	221210100	0.628
D_9	5.801	222111000	0.756
D_{10}	6.218	221211000 + 222110100	0.602 + 0.194

Table S28: Low-lying quartet states of **3˙** obtained from state-averaged CASSCF/CASPT2 calculations.

State	CASPT2 Energy (eV)	Dominant Configuration(s)	Weight(s)
Q_1	2.475	222111000	0.745
Q_2	3.019	222110100 + 221211000	0.699 + 0.134
Q_3	4.049	221210100 + 222111000	0.707 + 0.072
Q_4	2.666	212211000 + 222110010	0.443 + 0.309
Q_5	4.127	221211000 + 222110100	0.637 + 0.179
Q_6	5.193	122211000 + 222110001	0.237 + 0.226
Q_7	5.471	122210100 + 221210001	0.375 + 0.151
Q_8	5.297	212210100 + 221111100	0.363 + 0.176
Q_9	5.369	222101100 + 221111100	0.247 + 0.144
Q_{10}	6.506	221111100 + 222101100	0.325 + 0.180

Table S29: Density matrix for **3˙** obtained from state-averaged CASSCF/CASPT2 calculations.

	**0**	**1**	**2**	**3**	**4**	**5**	**6**	**7**	**8**
**0**	1.8825	0	0	0	0	0	0	0	0
**1**	0	1.8139	0	0	0	0	0	0	0
**2**	0	0	1.6586	0	0	0	0	0	0
**3**	0	0	0	1.4583	0	0	0	0	0
**4**	0	0	0	0	0.9514	0	0	0	0
**5**	0	0	0	0	0	0.5320	0	0	0
**6**	0	0	0	0	0	0	0.4823	0	0
**7**	0	0	0	0	0	0	0	0.1234	0
**8**	0	0	0	0	0	0	0	0	0.0976

Table S30: Spin density matrix for **3**$^{\bullet}$ obtained from state-averaged CASSCF/CASPT2 calculations.

	**0**	**1**	**2**	**3**	**4**	**5**	**6**	**7**	**8**
**0**	0.0777	0.0000	0.0000	-0.0041	0.0000	-0.0068	0.0000	0.0000	0.0017
**1**	0.0000	0.1182	0.0024	0.0000	0.0097	0.0000	-0.0022	0.0219	0.0000
**2**	0.0000	0.0024	0.2189	0.0000	0.0042	0.0000	0.0008	-0.0061	0.0000
**3**	-0.0041	0.0000	0.0000	0.3317	0.0000	-0.0104	0.0000	0.0000	0.0085
**4**	0.0000	0.0097	0.0042	0.0000	0.4374	0.0000	-0.0030	0.0062	0.0000
**5**	-0.0068	0.0000	0.0000	-0.0104	0.0000	0.3466	0.0000	0.0000	0.0028
**6**	0.0000	-0.0022	0.0008	0.0000	-0.0030	0.0000	0.3391	0.0031	0.0000
**7**	0.0000	0.0219	-0.0061	0.0000	0.0062	0.0000	0.0031	0.0712	0.0000
**8**	0.0017	0.0000	0.0000	0.0085	0.0000	0.0028	0.0000	0.0000	0.0593

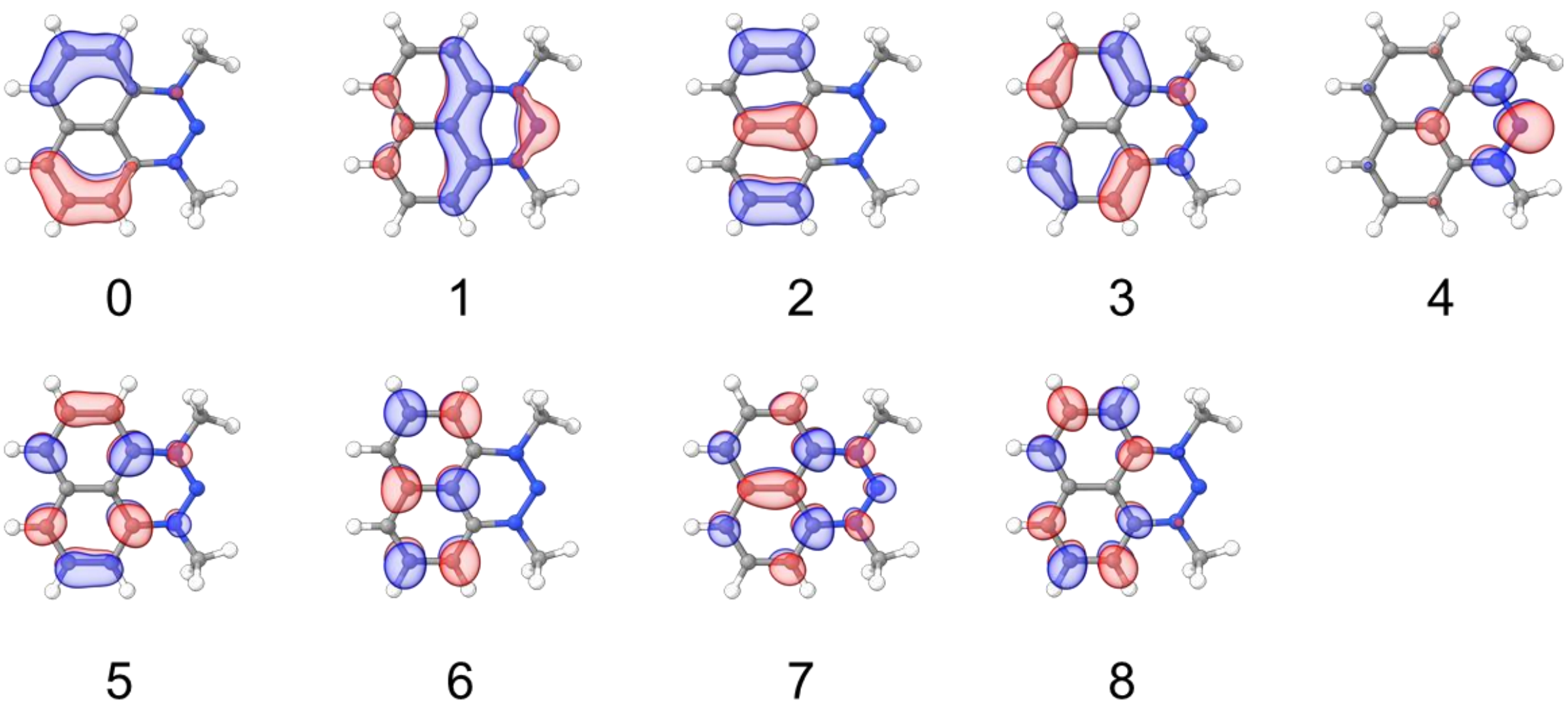

Figure S48: CASSCF orbitals of **3**$^{\bullet}$ (isovalue = 0.05).

4.5.3 CASSSCF of Int-1•

Table S31: Low-lying quartet states of **Int-1•** obtained from state-averaged CASSCF calculations.

State	CASSCF Energy (eV)	Dominant Configuration(s)	Weight(s)
Q0	0.000	222111000	0.801
Q1	1.763	222110100 + 221211000	0.627 + 0.162
Q2	2.594	212211000 + 222110010	0.398 + 0.348
Q3	3.010	221210100	0.622
Q4	3.437	122211000 + 222110001	0.320 + 0.204
Q5	3.674	221211000 + 222110100	0.524 + 0.235
Q6	4.022	122210100 + 221210001 + 222011100	0.281 + 0.127 + 0.110
Q7	4.475	212210100 + 221111100 + 221210010	0.327 + 0.178 + 0.101
Q8	4.900	221111100 + 212111010	0.429 + 0.104
Q9	5.195	222110010 + 212211000 + 222011100	0.348 + 0.182 + 0.109

Table S32: Density matrix for **Int-1•** obtained from state-averaged CASSCF calculations.

	**0**	**1**	**2**	**3**	**4**	**5**	**6**	**7**	**8**
**0**	1.8229	0	0	0	0	0	0	0	0
**1**	0	1.7589	0	0	0	0	0	0	0
**2**	0	0	1.6134	0	0	0	0	0	0
**3**	0	0	0	1.3567	0	0	0	0	0
**4**	0	0	0	0	0.9982	0	0	0	0
**5**	0	0	0	0	0	0.6034	0	0	0
**6**	0	0	0	0	0	0	0.4881	0	0
**7**	0	0	0	0	0	0	0	0.2217	0
**8**	0	0	0	0	0	0	0	0	0.1366

Table S33: Spin density matrix for **Int-1•** obtained from state-averaged CASSCF calculations.

	**0**	**1**	**2**	**3**	**4**	**5**	**6**	**7**	**8**
**0**	0.1318	–0.0001	–0.0002	–0.0149	0.0000	–0.0081	0.0002	–0.0001	–0.0107
**1**	–0.0001	0.1892	–0.0034	0.0002	0.0094	0.0006	–0.0055	–0.0128	–0.0002
**2**	–0.0002	–0.0034	0.2993	0.0002	–0.0117	–0.0006	–0.0095	0.0019	0.0003
**3**	–0.0149	0.0002	0.0002	0.4252	–0.0015	–0.0038	–0.0004	0.0000	0.0032
**4**	0.0000	0.0094	–0.0117	–0.0015	0.8389	0.0015	0.0104	–0.0135	–0.0001
**5**	–0.0081	0.0006	–0.0006	–0.0038	0.0015	0.4494	–0.0002	0.0002	–0.0113
**6**	0.0002	–0.0055	–0.0095	–0.0004	0.0104	–0.0002	0.3973	–0.0045	–0.0002
**7**	–0.0001	–0.0128	0.0019	0.0000	–0.0135	0.0002	–0.0045	0.1717	–0.0001
**8**	–0.0107	–0.0002	0.0003	0.0032	–0.0001	–0.0113	–0.0002	–0.0001	0.0974

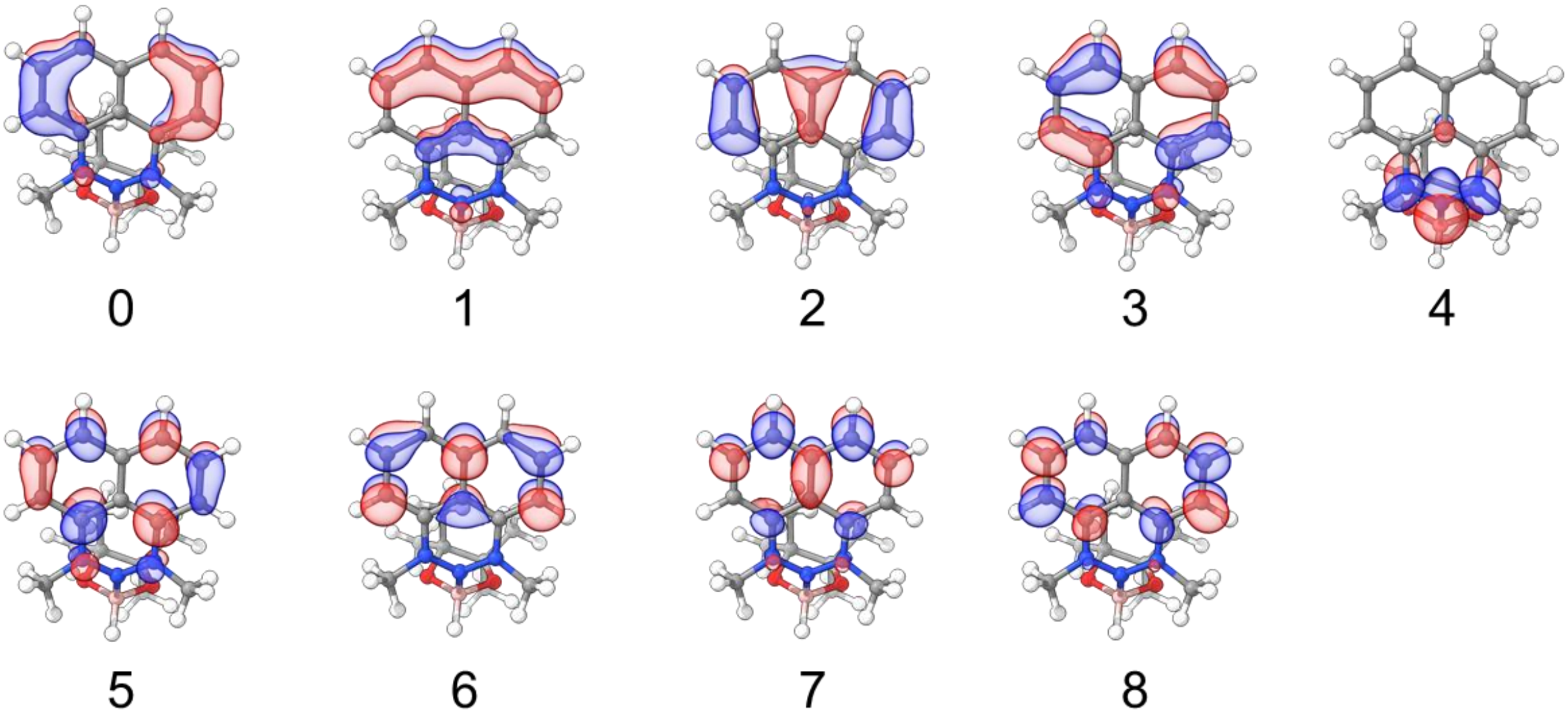

Figure S49: CASSCF orbitals of **Int-1˙** (isovalue = 0.05).

4.5.4 CASSCF of 4

Table S34: Low-lying triplet states of **4** obtained from state-averaged CASSCF/CASPT2 calculations.

State	CASPT2 Energy (eV)	Dominant Configuration(s)	Weight(s)
T1	2.448	22211000	0.748
T2	3.165	22210100 + 22121000	0.679 + 0.098
T3	3.225	21221000 + 22210010	0.344 + 0.338
T4	1.408	22120100	0.673
T5	4.211	21220100 + 22210001	0.455 + 0.155
T6	4.147	22121000 + 22210100	0.511 + 0.197
T7	5.245	12220100 + 22201100	0.375 + 0.137
T8	4.518	12221000 + 21220100	0.258 + 0.241
T9	4.906	22210010 + 21221000	0.371 + 0.250
T10	4.956	22120010 + 21220010	0.358 + 0.188

Table S35: Low-lying singlet states of **4** obtained from state-averaged CASSCF/CASPT2 calculations.

State	CASPT2 Energy (eV)	Dominant Configuration(s)	Weight(s)
S0	0.000	22220000	0.870
S1	3.101	22210100 + 22121000	0.525 + 0.229
S2	3.559	22211000	0.788
S3	4.542	21220100 + 22202000	0.243 + 0.153
S4	4.135	21221000 + 22210010	0.460 + 0.161
S5	4.656	21220100 + 22202000	0.241 + 0.132
S6	3.599	22121000 + 22210010	0.409 + 0.270
S7	5.075	22210010 + 22210100	0.280 + 0.140
S8	5.369	22120100 + 21220100	0.583 + 0.109
S9	5.565	22202000 + 12221000	0.246 + 0.146

Table S36: Density matrix for **4** obtained from state-averaged CASSCF/CASPT2 calculations.

	**0**	**1**	**2**	**3**	**4**	**5**	**6**	**7**
**0**	1.8587	0	0	0	0	0	0	0
**1**	0	1.7198	0	0	0	0	0	0
**2**	0	0	1.6699	0	0	0	0	0
**3**	0	0	0	1.4687	0	0	0	0
**4**	0	0	0	0	0.5413	0	0	0
**5**	0	0	0	0	0	0.4325	0	0
**6**	0	0	0	0	0	0	0.2091	0
**7**	0	0	0	0	0	0	0	0.1000

Table S37: Spin density matrix for **4** obtained from state-averaged CASSCF/CASPT2 calculations.

	**0**	**1**	**2**	**3**	**4**	**5**	**6**	**7**
**0**	0.0625	0.0001	–0.0001	0.0027	–0.0013	0.0000	0.0000	–0.0007
**1**	0.0001	0.1205	0.0146	0.0001	0.0000	–0.0024	–0.0015	0.0001
**2**	–0.0001	0.0146	0.1409	0.0000	0.0000	0.0024	0.0012	0.0000
**3**	0.0027	0.0001	0.0000	0.1715	0.0015	–0.0001	0.0000	–0.0005
**4**	–0.0013	0.0000	0.0000	0.0015	0.1873	0.0001	–0.0001	0.0012
**5**	0.0000	–0.0024	0.0024	–0.0001	0.0001	0.1792	0.0027	–0.0001
**6**	0.0000	–0.0015	0.0012	0.0000	–0.0001	0.0027	0.0981	0.0000
**7**	–0.0007	0.0001	0.0000	–0.0005	0.0012	–0.0001	0.0000	0.0400

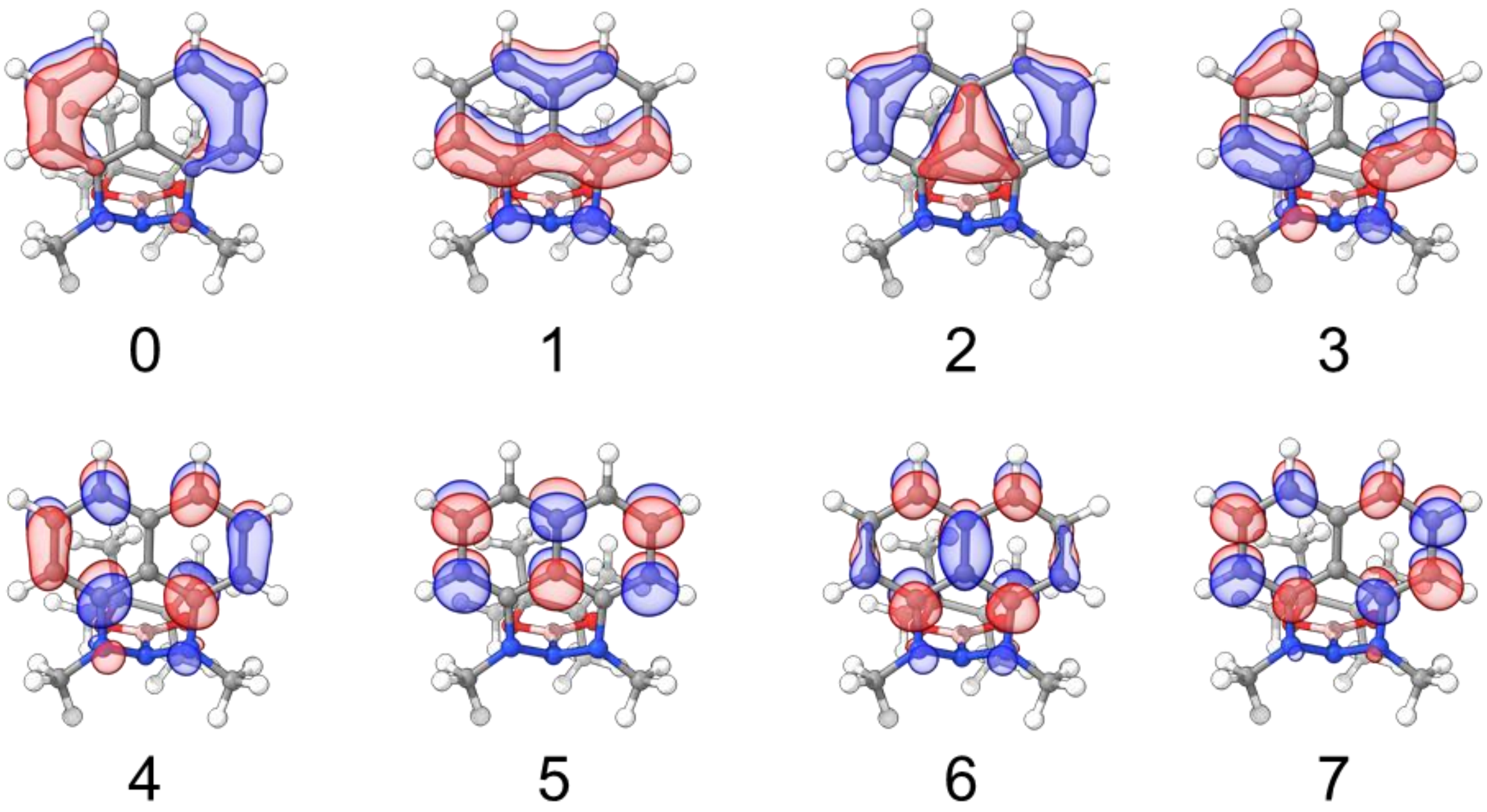

Figure S50: CASSCF orbitals of **Int-1˙** (isovalue = 0.05).

4.6 TDDFT Orbitals

4.6.1 TDDFT S_0->S_1 NTO transition of 2

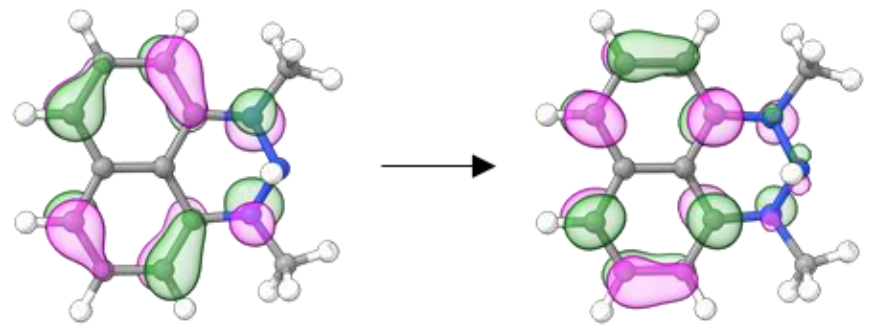

52A 53A

Oscillator strength = 0.957

Figure S51: S_0->S_1 Natural Transition Orbitals of **2** (isovalue = 0.05).

4.6.2 TDDFT D_0->D_4 Natural Transition Orbitals of 3$^{\bullet}$

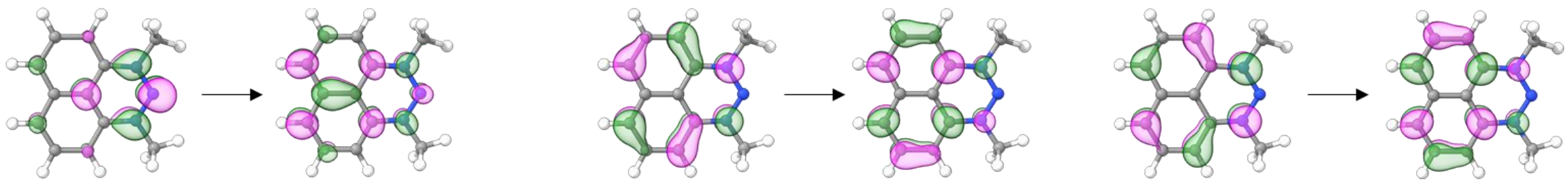

52A 53A 51A 54A 51B 52B

Weight = 0.537 Weight = 0.312 Weight = 0.137

Figure S52: D_0->D_4 Natural Transition Orbitals of **3**$^{\bullet}$ (isovalue = 0.05).

4.6.3 TDDFT Q_0->Q_2 Natural Transition Orbitals of Int-1$^{\bullet}$

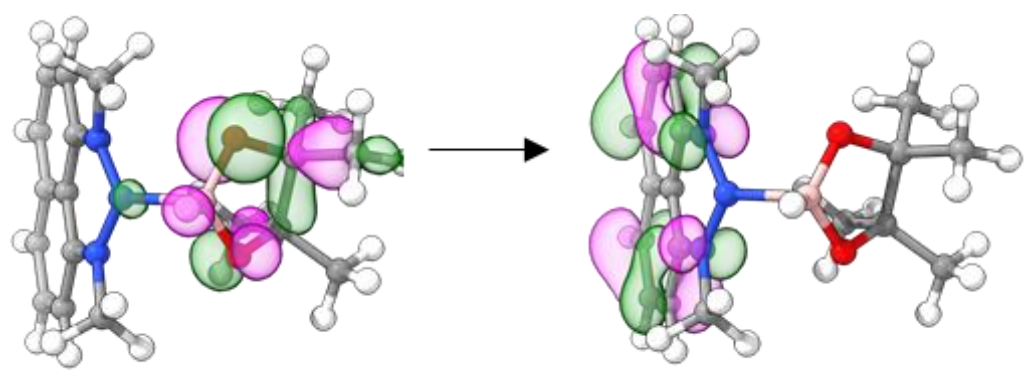

85B 86B

Weight = 0.999

Figure S53: Q_0->Q_2 Natural Transition Orbitals of **3**$^{\bullet}$ (isovalue = 0.05).

4.6.4 TDDFT S_0->S_1 Natural Transition Orbitals of 4

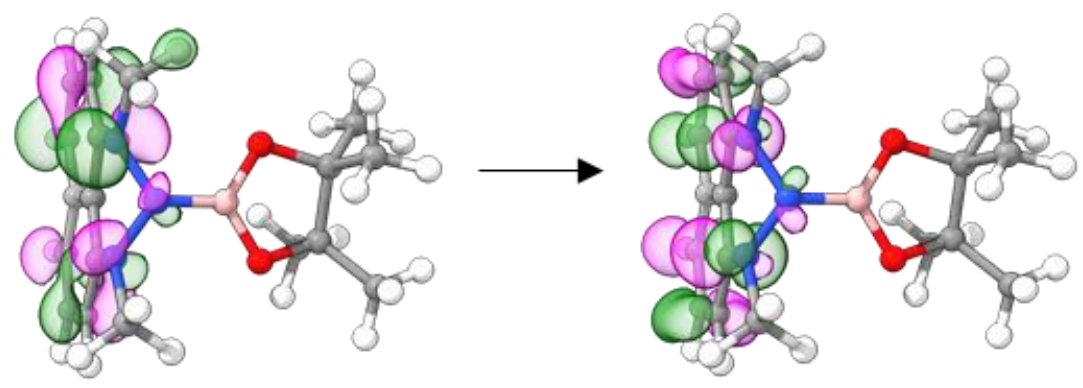

86A 87A

Weight = 0.965

Figure S54: S_0->S_1 Natural Transition Orbitals of **3**$^{\bullet}$ (isovalue = 0.05).

4. References

[1] Y. Zhang, A. M. Santos, E. Herdtweck, J. Mink, F. E. Kühn, “Organonitrile ligated silver complexes with perfluorinated weakly coordinating anions and their catalytic application for coupling reactions” *New Journal of Chemistry* **2005**, *29*, 366–370.

[2] M. Mehta, J. M. Goicoechea, “Nitrenium Salts in Lewis Acid Catalysis” *Angewandte Chemie International Edition* **2020**, *59*, 2715–2719.

[3] A. Pogoreltsev, Y. Tulchinsky, N. Fridman, M. Gandelman, “Nitrogen Lewis Acids” *Journal of the American Chemical Society* **2017**, *139*, 4062–4067.

[4] A. Koronatov, A. Mauda, B. Tumansky, A. Kaushansky, N. Fridman, D. Bravo-Zhivotovskii, M. Gandelman, “Multimodal Reactivity of N–H Bonds in Triazanes and Isolation of a Triazinyl Radical” *Journal of the American Chemical Society* **2022**, *144*, 23642–23648.

[5] R. J. Ely, J. P. Morken, “Regio- and Stereoselective Ni-Catalyzed 1,4-Hydroboration of 1,3-Dienes: Access to Stereodefined (Z)-Allylboron Reagents and Derived Allylic Alcohols” *Journal of the American Chemical Society* **2010**, *132*, 2534–2535.

[6] F. Neese, “Software update: The ORCA program system—Version 5.0” *WIREs Computational Molecular Science* **2022**, *12*, e1606.

[7] F. Neese, “The ORCA program system” *WIREs Computational Molecular Science* **2012**, *2*, 73–78.

[8] F. Neese, “The SHARK integral generation and digestion system” *Journal of Computational Chemistry* **2023**, *44*, 381–396.

[9] N. Mardirossian, M. Head-Gordon, “ωB97X-V: A 10-parameter, range-separated hybrid, generalized gradient approximation density functional with nonlocal correlation, designed by a survival-of-the-fittest strategy” *Physical Chemistry Chemical Physics* **2014**, *16*, 9904–9924.

[10] F. Weigend, R. Ahlrichs, “Balanced basis sets of split valence, triple zeta valence and quadruple zeta valence quality for H to Rn: Design and assessment of accuracy” *Physical Chemistry Chemical Physics* **2005**, *7*, 3297–3305.

[11] F. Weigend, “Accurate Coulomb-fitting basis sets for H to Rn” *Physical Chemistry Chemical Physics* **2006**, *8*, 1057–1065.

[12] E. Caldeweyher, S. Ehlert, A. Hansen, H. Neugebauer, S. Spicher, C. Bannwarth, S. Grimme, “A generally applicable atomic-charge dependent London dispersion correction” The *Journal of Chemical Physics* **2019**, *150*, 154122.

[13] A. V. Marenich, C. J. Cramer, D. G. Truhlar, “Universal Solvation Model Based on Solute Electron Density and on a Continuum Model of the Solvent Defined by the Bulk Dielectric Constant and Atomic Surface Tensions” The *Journal of Physical Chemistry B* **2009**, *113*, 6378–6396.

[14] B. Hammer, L. B. Hansen, J. K. Nørskov, “Improved adsorption energetics within density-functional theory using revised Perdew-Burke-Ernzerhof functionals” *Physical Review B* **1999**, *59*, 7413–7421.

[15] J. P. Perdew, K. Burke, M. Ernzerhof, “Generalized Gradient Approximation Made Simple” *Physical Review Letters* **1996**, *77*, 3865–3868.

[16] J. G. Brandenburg, C. Bannwarth, A. Hansen, S. Grimme, “B97-3c: A revised low-cost variant of the B97-D density functional method” The *Journal of Chemical Physics* **2018**, *148*, 064104.

[17] A. Hellweg, C. Hättig, S. Höfener, W. Klopper, “Optimized accurate auxiliary basis sets for RI-MP2 and RI-CC2 calculations for the atoms Rb to Rn” *Theoretical Chemistry Accounts* **2007**, *117*, 587–597.

[18] C. Kollmar, K. Sivalingam, Y. Guo, F. Neese, “An efficient implementation of the NEVPT2 and CASPT2 methods avoiding higher-order density matrices” The *Journal of Chemical Physics* **2021**, *155*, 234104.

[19] C. Kollmar, K. Sivalingam, B. Helmich-Paris, C. Angeli, F. Neese, “A perturbation-based super-CI approach for the orbital optimization of a CASSCF wave function” *Journal of Computational Chemistry* **2019**, *40*, 1463–1470.

[20] M. Ugandi, M. Roemelt, “A recursive formulation of one-electron coupling coefficients for spin-adapted configuration interaction calculations featuring many unpaired electrons” *International Journal of Quantum Chemistry* **2023**, *123*, e27045.
[21] C. Y. Legault **2020**.
[22] E. F. Pettersen, T. D. Goddard, C. C. Huang, E. C. Meng, G. S. Couch, T. I. Croll, J. H. Morris, T. E. Ferrin, “UCSF ChimeraX: Structure visualization for researchers, educators, and developers” *Protein Science* **2021**, *30*, 70–82.
[23] I. Avigdori, A. Pogoreltsev, A. Kaushanski, N. Fridman, M. Gandelman, “Frustrated Lewis Pairs Comprising Nitrogen Lewis Acids for Si–H Bond Activation” *Angewandte Chemie International Edition* **2020**, *59*, 23476–23479.
[24] A. Manian, S. P. Russo, “The dominant nature of Herzberg–Teller terms in the photophysical description of naphthalene compared to anthracene and tetracene” *Scientific Reports* **2022**, *12*, 21481.
[25] A. H. Zewail, “Femtochemistry: Atomic-Scale Dynamics of the Chemical Bond” The *Journal of Physical Chemistry A* **2000**, *104*, 5660–5694.

5. DFT Coordinates

2 Charge = 0 Multiplicity = 1

Energy: -629.833768441389

C	6.95759818623472	-1.20735416827290	1.15064892319849
C	6.11029894968096	3.33464522254952	1.65278120685842
C	4.14089654737639	-1.86299398620504	1.37115420637188
C	4.59933529508704	-0.56626442990466	1.41358965751381
C	2.77449582845215	-2.13069248611583	1.60896565826725
C	4.14738380474988	1.85560990854008	1.68249810877313
C	3.69144539348723	0.50083950414746	1.69726125051951
C	3.25558474940923	2.88116174742312	1.89813021658103
C	1.88654869048474	-1.12863909073792	1.88544958945628
C	2.32730572033220	0.22083636113910	1.92513029334463
C	1.89410145130993	2.58704471472452	2.13318250080568
C	1.43332241855573	1.30008972272115	2.15520515309447
H	6.74249560652752	-1.95538228468726	0.38665227598769
H	5.61270346519132	4.09989521287838	1.05583488761431
H	7.90170961570524	-0.72210818997205	0.90483240031432
H	4.81419157796634	-2.68389271876543	1.16116824290398
H	7.15486573166482	3.28078229717923	1.34752262317318
H	7.05367646460890	-1.71237423464033	2.12227805816608
H	2.43406726342662	-3.16096541336098	1.57541299786678
H	3.58285936807511	3.91280044711287	1.89373045376868
H	0.83869795651847	-1.34535056840634	2.06554526871847
H	6.05839317368541	3.62350817991300	2.71211785002945
H	1.20768321006950	3.41019170640848	2.30563805882879
H	0.38505020676848	1.08564469080606	2.33553704709591
N	5.92037097270520	-0.19522419972483	1.15065498303226
N	6.31210976833138	0.97348718531711	1.84424798280895
N	5.50238123011615	2.04395447220059	1.39831632174590
H	6.12243035347919	0.82775139773290	2.84350678316059

2 Charge = 0 Multiplicity = 3

Energy: -629.734221161627

C	6.96890960320050	-1.19568900734302	1.15058249680596
C	6.12446054143648	3.32847001574098	1.64675432709287
C	4.11335871753257	-1.91442240837956	1.35895987275660
C	4.58897169118494	-0.55131786709466	1.41460037385295
C	2.80554920690236	-2.18059746587870	1.59455988189977
C	4.14299300806117	1.83796558306024	1.68082331413517
C	3.70157842800531	0.50288384349252	1.69455121565654
C	3.21149074428274	2.92052332693693	1.89884240588365
C	1.88283870531135	-1.11801056140377	1.88421960732902
C	2.30956404809657	0.21727364796799	1.92666597420366
C	1.90521949115073	2.64648548204002	2.13389773940736
C	1.43397491656495	1.28914609443932	2.15309385649388
H	6.72973278754936	-1.95875198547095	0.40987293406974
H	5.59794641848873	4.09230154308018	1.07443303177895
H	7.89287726950480	-0.70106300619243	0.85123037334647
H	4.80568192131101	-2.71905647947964	1.14435369410554
H	7.15066054470375	3.27003826278604	1.28418674819108
H	7.12238995587829	-1.67989618342261	2.12477425442101
H	2.44406021528109	-3.20279009297322	1.56665803159274
H	3.56305441315125	3.94480360677201	1.88883775593025
H	0.83575684826221	-1.34539491158129	2.05435001241290
H	6.13600285796651	3.62064280578764	2.70579759140223
H	1.20249118006532	3.45341934999481	2.31098424024150
H	0.38230400197080	1.08669161989376	2.32633222673727
N	5.91773709747380	-0.19790816922565	1.18626317973394
N	6.30878897952222	0.96757326338924	1.89329730493638
N	5.49996438550018	2.03773003151343	1.43353005093849
H	6.08364502164081	0.81595066155038	2.88454050464398

3$^{\bullet}$ Charge = 0 Multiplicity = 2

Energy: -629.209041800601

C	6.90589114895718	-1.25373086587439	1.27453061116560

C	6.04897595047438	3.33496304693036	1.78226684854588
C	4.11234335199051	-1.87707753861232	1.49056520066383
C	4.56177080359799	-0.57180830854336	1.56703350604793
C	2.73126535555290	-2.13706108190750	1.60065234759749
C	4.11536354183050	1.81641219604199	1.83120794020144
C	3.62887763780186	0.48341931923489	1.75538830462215
C	3.22678206895332	2.85957993286855	2.01541176770490
C	1.81466159652693	-1.13518071821373	1.78187252123675
C	2.24980432463769	0.21339340094764	1.86449449768648
C	1.84946213620183	2.57953044862807	2.12343516517510
C	1.35890315636667	1.30244792983819	2.05211596561668
H	6.73603230158434	-1.79757078184026	0.34089795365096
H	5.65255282579106	3.97043183823094	0.98537141606053
H	7.87196013780580	-0.75572965037400	1.22874735129370
H	4.80157771242492	-2.69852309929231	1.34790594746563
H	7.12542084059488	3.23637997750604	1.65989982853992
H	6.90545566267190	-1.96421833558339	2.10598236734980
H	2.39470484414660	-3.16743558953549	1.53821574877054
H	3.57077424156465	3.88329928548124	2.07709806086712
H	0.75570851820283	-1.35610246781306	1.86407956601906
H	5.83769793444653	3.80126733103276	2.74884369029550
H	1.16503662807117	3.40999728076177	2.26740277417872
H	0.29502407911659	1.10795351728255	2.13735449836726
N	5.89154232796620	-0.23676219462432	1.46806819219848
N	6.39643568104039	1.02491240982832	1.53548297486523
N	5.47197219168030	2.00721571760078	1.71467895381328

3$^{\bullet}$ Charge = 0 Multiplicity = 4

Energy: -629.113668443208

C	6.91122889101500	-1.25107276627633	1.27990414091450
C	6.05441268775700	3.33423673316757	1.77597730475222
C	4.08725083458018	-1.92685873474570	1.48640917364819
C	4.54965444777147	-0.56157934281306	1.56925011389523
C	2.76035238756116	-2.18985555788802	1.58971312564173
C	4.10774814700922	1.80252050103989	1.83095154521668
C	3.64096591777930	0.48587919456878	1.75470314551373
C	3.18558442810586	2.89664477463995	2.02552885832776
C	1.81551279486445	-1.12560314181079	1.78074001274661
C	2.24149419514102	0.21169668821017	1.86509734184402
C	1.85772016419116	2.63959660193617	2.13115568164887
C	1.36327238040890	1.29387148288521	2.05200469914984
H	6.75433063886680	-1.78781549200973	0.34084134326035
H	5.65473585213993	3.97410598308272	0.98562776798970
H	7.87331827664469	-0.74471160365602	1.24548005128305
H	4.79607762951733	-2.73258826924870	1.34232279531705
H	7.12810325238477	3.22622656896524	1.63902347611764
H	6.91062045879994	-1.96479119876381	2.10733177626968
H	2.40426527795627	-3.21219586177521	1.52797889814741
H	3.55315005693658	3.91329355042399	2.08649884395209
H	0.75733173241870	-1.35019704483164	1.86149251246875
H	5.86170012535381	3.79735267217555	2.74710280283258
H	1.15819941415574	3.45509874960708	2.27751234491883
H	0.29884906065401	1.10317579377045	2.13750095696477
N	5.88785782476271	-0.24025470916023	1.47257130497906
N	6.39568135750790	1.02477447345904	1.53568339061005
N	5.46657876571595	2.00905295504736	1.71060059158954

Int-1$^{\bullet}$ Charge = 0 Multiplicity = 4

Energy: -1041.337252860918

C	7.12495135806381	-0.88670811737878	0.42845939712502
C	6.27254647617642	3.62309237086728	1.20541915199000
C	4.48121701634651	-1.70493483480154	1.38830990703313
C	4.90425512390528	-0.32540978144413	1.39599694203068
C	3.21519134106112	-2.00972965346168	1.76515718710721
C	4.44242390396414	2.02604742939628	1.73998787007685
C	4.02504170029133	0.69532693826383	1.77147035978356
C	3.54216440331599	3.09427033344815	2.10088783815739

C	2.29949934527266	-0.97669083063482	2.16087600859651
C	2.68649653056308	0.37404124261348	2.16534609347655
C	2.27529913481313	2.79048156608022	2.47898218795082
C	1.82765916631215	1.42953111299042	2.51576801595162
H	6.64208232550313	-1.34303453078402	-0.43827014521752
H	5.61945865603538	4.14946587448104	0.50691526401223
H	7.99629142627492	-0.32948658138357	0.09540022912382
H	5.17390299943899	-2.48404485919206	1.09598760426654
H	7.26769133136056	3.55542213660159	0.77477499764709
H	7.43969300656060	-1.65855947620769	1.13043301598535
H	2.88399322424413	-3.04204577000091	1.77065638699152
H	3.88018103155110	4.12235895358648	2.07935787408245
H	1.28803364365409	-1.23701958808317	2.45355565220359
H	6.32507555412349	4.16602447922428	2.14930669291443
H	1.58835083132622	3.58206132047257	2.75659640964614
H	0.80807929680237	1.20560423403099	2.81050134075199
N	6.19143102810387	0.04328914219025	1.04604078712011
N	6.67734872467935	1.27938030051480	1.31561496853716
N	5.76046060380993	2.27440341907932	1.39680073523055
H	8.97333352519068	1.84538850666064	1.19424737623649
B	8.26788715270764	1.46924786647794	2.11197884428383
O	8.53888520637352	0.18510357078611	2.68249986514514
O	8.08838542441524	2.40743847029974	3.17108475045498
C	8.68733630685036	0.35298139677184	4.09909368224599
C	7.88663043201096	1.66108855145572	4.38076720929099
C	8.40800153866582	2.48171376080461	5.55032048870401
H	7.77504916617899	3.36243398564373	5.69230572905651
H	8.38454512384894	1.89686310408053	6.47436257034231
H	9.42979500663481	2.82138631452648	5.37700963924827
C	6.39217226327716	1.40352257658542	4.57424657494959
H	6.19299864112426	0.96577549425648	5.55595483848978
H	5.85623507577591	2.35394243589615	4.51172427138108
H	5.99073756662200	0.72934384081653	3.81753874966809
C	8.14950842334683	-0.88082617707079	4.80750537302469
H	8.77711089164567	-1.74449404748391	4.56996478041820
H	8.16323670606891	-0.74250079785268	5.89262874231045
H	7.12854700199624	-1.10707231453907	4.49671363307464
C	10.17879744198120	0.50791766637810	4.39528940239892
H	10.37727527227004	0.56139057855825	5.46884388388916
H	10.70882627886316	-0.35986262829550	3.99368957538527
H	10.58288137059218	1.40507601477428	3.91989824742419

4 Charge = 0 Multiplicity = 3

Energy: -1040.790643164626

C	6.82419699642035	-1.38765508280534	0.64340780584341
C	6.35080121442094	3.13931272481810	1.03539708008439
C	3.99791541205193	-1.94510226055257	1.48049738462165
C	4.57272581619286	-0.61579893505286	1.43149310796791
C	2.72976877229501	-2.10721124417367	1.92788501271916
C	4.32837014236961	1.81187453494500	1.65990981074398
C	3.81039380359182	0.51284986574110	1.80312210870526
C	3.52478630588501	2.95878788345453	1.97765886753278
C	1.94272483002153	-0.97523811364091	2.33208489982431
C	2.46030627874434	0.33684046847801	2.26371728668069
C	2.25559535262642	2.78622366300929	2.43225452093872
C	1.70685006632356	1.46851058217263	2.58111879166628
H	6.36169218095746	-2.04218554763773	-0.09673671119197
H	5.76257253751516	3.81111071582184	0.40775960357381
H	7.67606784683050	-0.88811653911113	0.18071739534646
H	4.59483369094892	-2.79946878564967	1.18404680456663
H	7.29325584351912	2.92955052557307	0.52785028623500
H	7.18055368803820	-1.99216664460782	1.48531883035996
H	2.29510905523861	-3.09930764805000	1.98579635584384
H	3.94237409484666	3.95312328731192	1.86927865478275
H	0.92273611421759	-1.12349887547480	2.67068831019016
H	6.56342855434869	3.63546450022967	1.98939599457117
H	1.64655468294005	3.64660345517280	2.68792963264570

H	0.68559346061502	1.34671660159519	2.92706120085248
N	5.87824993029112	-0.37270605408590	1.04944233944360
N	6.40823130543115	0.79094677414172	1.63939160380669
N	5.63925523848384	1.88974634275751	1.19229004737912
B	6.69276874824633	0.71540957014049	3.05828225790443
O	6.92298119994589	-0.46912410664993	3.70508423684310
O	6.81564222272479	1.82707824398079	3.84922224813698
C	7.45489946815898	-0.11329056788985	5.01516554982260
C	6.89476556793569	1.33119406438797	5.21804868096517
C	7.79149741263420	2.24940645789378	6.02494278843938
H	7.31775312527440	3.22902376243502	6.12706158637610
H	7.94544350094815	1.84032335622337	7.02695470286810
H	8.76319822482401	2.38466436590433	5.54902216773851
C	5.47474053774481	1.34616369866289	5.77185509848184
H	5.45802894792985	1.05004265639250	6.82290274579429
H	5.07615831187015	2.36061056518793	5.69511792936346
H	4.81844489227285	0.67723238746116	5.20908734497940
C	6.97054608597905	-1.12716759586236	6.03318148541972
H	7.42000625281179	-2.10120382539239	5.82407001921458
H	7.27075351712195	-0.82375226031589	7.03960415229239
H	5.88592714763036	-1.23612702632124	6.00893233728417
C	8.97366131586357	-0.15138443191688	4.90189665296839
H	9.44561478417797	0.04203908618087	5.86753260677014
H	9.27852566359716	-1.14498570023426	4.56477026622118
H	9.33970285714163	0.58364010535165	4.18048411638166

Int-2 Charge = 0 Multiplicity = 3
Energy: -1225.612385696543

C	6.69100476869541	-1.34884297975524	1.10697441552028
C	6.12593968870125	3.27892969189863	1.58673297034946
C	3.90434008624004	-1.82800958552767	1.65228709106554
C	4.42745428740803	-0.55633394332770	1.71044504805362
C	2.51723612855633	-2.00752060427556	1.83924161646491
C	4.12857076063611	1.86292621106056	1.91752108716440
C	3.57769271205334	0.55659599957801	1.93235038858689
C	3.31139019542917	2.96099242028807	2.06297325446559
C	1.67651497194996	-0.95167994035338	2.06599320722525
C	2.19097928524595	0.36999538114471	2.10522792368692
C	1.92643972561477	2.76520494412158	2.24771077384264
C	1.37096819151995	1.51470417736844	2.27734752591953
H	6.80963727629811	-2.10804459999134	1.88095889387355
H	5.62863398233163	3.80784290650535	0.77037757666407
H	6.29209907539721	-1.80546534198108	0.19785382871008
H	4.53796355130129	-2.68695955610900	1.47712110655244
H	7.17113125545359	3.13868399899420	1.33054359125668
H	7.65684382508922	-0.90502730976682	0.88459966933184
H	2.11805007564583	-3.01598072163571	1.80140795197650
H	3.71266724384331	3.96518931157423	2.05069430101529
H	0.61176522168388	-1.10720058319741	2.20323137006900
H	6.04915500621464	3.86361009581509	2.50447301404469
H	1.29444610251185	3.63875698753103	2.37121665853904
H	0.30343577120880	1.38261597944800	2.41755379225370
N	5.77850065825208	-0.29860752339641	1.52871831609639
N	6.31330847729578	0.90261610483983	1.83260479782911
N	5.49948921460653	1.97550532531736	1.73551519071663
B	7.76506466040861	0.99927175251365	2.74648318923770
O	7.95074885108626	-0.24512787068673	3.39345770533370
O	7.62427923609478	2.01424738788686	3.71929837130253
C	7.81652537355814	-0.07348525742766	4.81418501310384
C	7.24867355510395	1.38838249360679	4.95741196378901
C	7.85532026195777	2.18970415055690	6.09913332486894
H	7.39904893494731	3.18310768135046	6.12750442136723
H	7.66450555801165	1.70280500691465	7.05919603223968
H	8.93185877218377	2.31199037769148	5.97505213254694
C	5.72506715892809	1.43137667663054	5.07602548782870
H	5.39709942584832	1.07547502926419	6.05576343337665
H	5.39108271723415	2.46541808142505	4.96018388335370

H	5.23193123110046	0.82350151599419	4.31778682618180
C	6.88866603780146	-1.15918684605480	5.34625284728320
H	7.36347054184521	-2.13535717984253	5.21483461032838
H	6.69597179611176	-1.01933069168094	6.41353024804039
H	5.93500165324426	-1.17316819753386	4.81718971528059
C	9.19576309295958	-0.25659647520145	5.44187875011504
H	9.14090470809270	-0.22640907591555	6.53303933370729
H	9.58492305332070	-1.23544409883940	5.14876809314548
H	9.89919188827676	0.50383048576666	5.10529138789395
O	8.67514020697258	1.29992722815072	1.60738069642045
N	10.04465495297927	1.57332159279595	1.95127160378019
N	10.44414479274834	1.69551338646706	3.02502456820055

•OBpin Charge = 0 Multiplicity = 2

Energy: -486.856333636267

C	-4.76750052598813	3.77701601995840	-0.11035601467367
C	-6.03094484408994	2.85831401102736	-0.16991413580605
B	-4.16759699835124	1.61587963979386	0.00542801095582
O	-3.73470529845666	2.85698346505138	0.37762735550060
O	-5.44846443589780	1.54463184364605	-0.46390672562229
C	-4.87762475612865	4.93788991415706	0.85795662895896
H	-3.96100068334513	5.53180712285971	0.82540989278478
H	-5.71212533538584	5.58348595068849	0.57207010112048
H	-5.03271114873484	4.59687547059097	1.88175358163516
C	-6.74448379539250	2.73575139101152	1.16955040582434
H	-7.26270638525817	3.66288687229407	1.42273542053950
H	-7.48574047108388	1.93597219650349	1.10401734219909
H	-6.04753938296012	2.49462288256713	1.97616761669364
C	-7.01288490400854	3.20771671327066	-1.27051677433951
H	-7.86427099395815	2.52386215274330	-1.23320656801407
H	-7.38663243021427	4.22502720367389	-1.12799439567504
H	-6.55526993835765	3.13738744359723	-2.25743594643722
C	-4.31571475334466	4.26064755899508	-1.48169333916483
H	-5.00565602570695	5.00729510987928	-1.88012483826837
H	-3.32974177055281	4.72143831153875	-1.38730566638452
H	-4.24400033981046	3.43601044780263	-2.19538838424766
O	-3.35927825297333	0.50160494834947	0.09677118242087

HBpin Charge = 0 Multiplicity = 1

Energy: -412.221793384277

C	-4.77176908574227	3.78268657901945	-0.11078909568677
C	-6.03052132870650	2.86006298442255	-0.16842563962200
B	-4.15054096535324	1.60605758040177	0.01141418881207
H	-3.44908806664138	0.64427556141224	0.09518058771095
O	-3.73044184234931	2.86356974135770	0.33914297610928
O	-5.44450543852160	1.54694140263301	-0.42109729958503
C	-4.87435623981377	4.93297306089094	0.87257838727476
H	-3.95826776155202	5.52794698713365	0.83841386660345
H	-5.71156942430874	5.58294239815381	0.60498761725623
H	-5.01788470887827	4.58050204462128	1.89429837304667
C	-6.76284959686921	2.76138267465783	1.16442149548761
H	-7.28354659242024	3.69261440643404	1.39717146298638
H	-7.50416088763417	1.96115135137189	1.10253741740632
H	-6.07626842278755	2.53086374564998	1.98269812482786
C	-7.00554940477948	3.18795516487454	-1.28311963523309
H	-7.85306003845196	2.49917433719867	-1.24292937491392
H	-7.38834146969061	4.20472316643920	-1.16187628621713
H	-6.54010320480706	3.10438418874674	-2.26547898724050
C	-4.34017295838124	4.29320013102270	-1.48035309110624
H	-5.04013093138940	5.04030171551608	-1.86048495635057
H	-3.35677211735576	4.76062183546142	-1.38997280643741
H	-4.26844851356610	3.47991394258043	-2.20667332512895

$O(Bpin)_2$ Charge = 0 Multiplicity = 1

Energy: -898.628582480275

C	-4.74750394813356	3.42415951380140	-0.75002166176396
C	-5.26512492641182	3.46556610713648	0.72512965680321

B	-3.99366822842667	1.62490489978688	0.39504639598045
O	-3.69638185682613	2.41935693821934	-0.68192586964994
O	-5.01066567530920	2.10618169697611	1.17947648652520
C	-4.15536235866499	4.72833119752064	-1.24813726442369
H	-3.83369264727664	4.61304902891004	-2.28627454506545
H	-4.90675891131183	5.52143213614309	-1.21158802545821
H	-3.29446539145441	5.03403705238970	-0.65286875947418
C	-4.44495130096153	4.38989814870900	1.61727363551640
H	-4.63083484324980	5.43854375973894	1.37587297151335
H	-4.73064261758585	4.22175036480107	2.65833436876963
H	-3.37416335124061	4.19356228034029	1.51974485057076
C	-6.74442715715481	3.76700052717002	0.86716059337478
H	-7.01510164408520	3.78814118713646	1.92582585607375
H	-6.97236439479943	4.74640439764461	0.43854744743113
H	-7.35834035326781	3.01570671999547	0.36965012704306
C	-5.79267219551404	2.90529741534073	-1.73038302716899
H	-6.58979553796070	3.63625858302022	-1.88134303535584
H	-5.31262457392145	2.71852340155684	-2.69394287925260
H	-6.23809119594134	1.97032047437086	-1.38108667858760
O	-3.34862846025912	0.43741935146242	0.60960487154704
B	-3.08205358393076	-0.17713754022154	1.80199065383341
O	-2.95185715233281	-1.53882479472108	1.89607328391509
O	-2.87171918821761	0.48604330115755	2.98410707705104
C	-2.39774829590111	-1.80506025581020	3.21579835148228
C	-2.82838462201954	-0.53527579508120	4.02032452787038
C	-2.96958812103198	-3.10828127994175	3.73974664221034
C	-0.88720031493289	-1.91637535521781	3.04766733942445
C	-4.23716887294446	-0.64358297669663	4.59170104322416
C	-1.85103366959892	-0.10615241631697	5.09739655653526
H	-2.60915962845610	-3.93963703923447	3.12864230529334
H	-2.64212774926361	-3.27667724987024	4.76897870052655
H	-4.05965010069016	-3.10982898306912	3.71432810244557
H	-0.40342567434685	-2.17787250115844	3.99112542946804
H	-0.66870745120716	-2.70298129527272	2.32152118769074
H	-0.45566064114622	-0.98135995669695	2.68130774209795
H	-4.27343679794821	-1.35115035543218	5.42268147626958
H	-4.54453441814268	0.33702815563729	4.96261449010905
H	-4.95385703896751	-0.96230834933414	3.83042516416032
H	-2.23669735371530	0.77848741490654	5.61034693381984
H	-1.73018433782989	-0.90185713780062	5.83713437380792
H	-0.87293441761915	0.13647022800403	4.68102410381626

H_2 Charge = 0 Multiplicity = 1

Energy: -1.180852533780

H	3.89280217430106	1.04292100000000	0.01365300000000
H	3.14805782569894	1.04292100000000	0.01365300000000

N_2 Charge = 0 Multiplicity = 1

Energy: -109.593938594146

N	-0.00489042459340	0.00000000000000	0.00000000000000
N	-1.09510957540660	0.00000000000000	0.00000000000000

N_2O Charge = 0 Multiplicity = 1

Energy: -184.769901534415

N	0.00289439879746	-0.01158414690469	-0.02015911938583
N	-0.35648743115036	-0.53916882701262	-0.93394322779760
O	0.38357903235290	0.54775397391732	0.94810634718341

TS1 (ground state direct homolytic cleavage) Charge = 0 Multiplicity = 1

Energy: -629.736655580182

C	6.93656021034446	-1.18469096646519	1.15027175165166
C	6.09924384222190	3.30654913436391	1.65075339746208
C	4.13067007499686	-1.85831049979760	1.37419035616840
C	4.55121483081213	-0.55471234115609	1.39449498601883
C	2.76461262551302	-2.13434156692162	1.60572014713655
C	4.10577043668421	1.83244069910391	1.65903434995029
C	3.64391166418001	0.49717874940159	1.65274455273795

C	3.24724543395955	2.87324833064145	1.89723874675600
C	1.86226252632968	-1.13712012652472	1.85528670586907
C	2.28277913553889	0.21709890743517	1.88553983676575
C	1.88273745734099	2.58801201758651	2.12699084596927
C	1.40599584815210	1.30594162957834	2.12483717882429
H	6.72657488471673	-1.90338255686426	0.35921916613403
H	5.61806273020281	4.05235104877437	1.01931679524228
H	7.87759767440865	-0.68026653306770	0.94689507905948
H	4.81813877538586	-2.67196111996474	1.18842030378051
H	7.15015442719125	3.22503993677611	1.38579119136472
H	6.98511159659579	-1.69472691474026	2.11784961004830
H	2.43585568963328	-3.16780394619610	1.58629569745503
H	3.59071286259376	3.89846534384446	1.91500704976645
H	0.81658247434415	-1.36646057135744	2.03001175812971
H	5.99795151101023	3.58451292303416	2.70487152509748
H	1.20629887854744	3.41532497101523	2.31273832659776
H	0.35502074833850	1.10489028432537	2.30257131979321
N	5.88896162776576	-0.17802750362314	1.18317330831775
N	6.32682370870507	1.02182260581963	1.43096321558422
N	5.48100246686430	2.01030874233257	1.42771556336931
H	6.67414885762186	0.86562032264599	3.38905023494933

TS1 (triplet state bimolecular) Charge = 0 Multiplicity = 3
Energy: -1259.565486411868

C	1.88107008204151	-0.25776174419751	-1.91887733163406
C	-1.25501965062368	3.21541480959096	-1.68951682455078
C	-0.20990117855557	-2.29050473006439	-2.10705716216690
C	-0.51345147120206	-0.86835517457558	-2.01805125983976
C	-1.21483067511416	-3.19248103737913	-2.05830384070352
C	-2.14492792353683	0.94400496283025	-1.92404318725469
C	-1.84978738246749	-0.42066451731246	-1.91992325851273
C	-3.50475866967624	1.38253600309837	-1.81264268903709
C	-2.57988724645721	-2.76240008033483	-1.92527142085458
C	-2.91564783536468	-1.38412893069875	-1.85836594491559
C	-4.51133183674533	0.46894338296398	-1.72480583637530
C	-4.22595598094850	-0.93307838757752	-1.75577996291679
H	2.19533257430001	-0.89574429326056	-2.74536645683872
H	-1.98809325710363	3.70843391284803	-2.32755633300504
H	2.45338865746564	0.66790047539061	-1.94783348719005
H	0.81838417354156	-2.61610468325653	-2.19984464657853
H	-0.30150038272761	3.73369113138964	-1.78583905792375
H	2.08535773111916	-0.77141767814090	-0.97273684491666
H	-0.99884313762444	-4.25373063079887	-2.12049579918013
H	-3.73443585493453	2.44078781318871	-1.79743400349839
H	-3.37494775741728	-3.50004659707416	-1.89420622776486
H	-1.59033023928485	3.28430950900694	-0.64399451310383
H	-5.53960152635538	0.80137133292008	-1.62849117306105
H	-5.03667424129834	-1.65310169584953	-1.70504016473467
N	0.47826806870651	0.08676995083265	-2.07174094558258
N	0.16546504372819	1.36514432005897	-1.56439962928842
N	-1.06302281092604	1.83616701108442	-2.11865710889516
H	0.04701633186742	1.27984432069019	-0.55419274762600
H	-0.48426160806375	1.11093946244844	0.90174109465406
N	-0.52785377985347	0.89902522609261	1.89917678109264
N	-0.42759630856975	-0.49703304988228	2.08058371176801
N	0.54289901284330	1.57190346576369	2.54414585053725
C	-1.65114817049782	-1.18587472767401	1.70535816049336
C	0.79592204226896	-1.07947340526242	1.75491390847226
C	0.30948125731103	3.00644838732660	2.49145567333442
C	1.82510240637376	1.12091781344736	2.18904019919638
H	-1.76179318457667	-2.09090962798778	2.30460952651288
H	-2.48945511623094	-0.52136796596223	1.91038582106428
H	-1.67104980069372	-1.46401192847662	0.64407569594968
C	0.93261020483358	-2.41367002289318	1.43781010292760
C	1.95605746866126	-0.25408095699145	1.83904682555270
H	0.91192166417232	3.51281925627011	3.24538722602405
H	-0.74365067231682	3.18509908837257	2.70325988566615

H	0.55410118850374	3.42453410246078	1.50396508341326
C	2.94520579509463	1.91970969372405	2.23626371991824
C	2.21758679252393	-2.95866420476228	1.22559893840175
H	0.06662865425468	-3.05758219843920	1.36102847466620
C	3.23649677112463	-0.80817532641005	1.61525142202988
C	4.22036554432149	1.36073640419798	1.99535100446771
H	2.87006870154159	2.97287151199630	2.47136831878728
C	3.34527763281330	-2.18996158837051	1.30841280397173
H	2.30039932764879	-4.01480480453367	0.98820845134651
C	4.37590447564301	0.03400881143035	1.70753827276075
H	5.08954606874270	2.00949237242337	2.04299716571708
H	4.32779520901481	-2.61980926749462	1.14029235485100
H	5.36210481870526	-0.38888527618701	1.54320138437254

TS1 (triplet state direct homolytic cleavage) Charge = 0 Multiplicity = 3

Energy: -629.670351654074

C	6.94425305569964	-1.19169953108684	1.06483279588251
C	6.10217340338562	3.33469199855836	1.59209792121171
C	4.13219191729176	-1.87539244773317	1.37523480874697
C	4.61271652561128	-0.54500854824550	1.48781548860462
C	2.79254956929305	-2.13640436871256	1.58673115379752
C	4.17038627857219	1.82548656874870	1.74818838267481
C	3.69852535138897	0.49271578393907	1.77825189289463
C	3.24077141376209	2.88966792747322	1.88841246254156
C	1.88422917742068	-1.11908035908986	1.87551187765677
C	2.32434899134247	0.21725108501066	1.94925356060795
C	1.90546500226958	2.60473952974447	2.09452006843881
C	1.43315306324550	1.29354582304836	2.13435703195687
H	6.63805093759817	-1.88901295077652	0.28632737747542
H	5.54991906414216	4.02595821968935	0.95723517287120
H	7.82933848453895	-0.65312458980085	0.73388214561156
H	4.81239235615975	-2.68691197009042	1.15675430208545
H	7.11403906282170	3.22747646343066	1.20834425693764
H	7.17142345332827	-1.73631438336095	1.98665759565120
H	2.44267675957724	-3.16085335419862	1.52431333721710
H	3.57639416030971	3.91723996712197	1.86647401320555
H	0.83384147234085	-1.34326069823682	2.02266184380619
H	6.13794530858588	3.71575669318891	2.61718023224660
H	1.20953590985052	3.42559405451391	2.22737663175652
H	0.37835670641325	1.09231171466078	2.28306340402059
N	5.89823404771199	-0.21058260215638	1.30057770120659
N	6.40618078868341	1.00121303741279	1.73926912720230
N	5.48293018607330	2.01963826315809	1.56312171857124
H	6.13998055258132	0.73135967378906	3.62854669511982

TS2 Charge = 0 Multiplicity = 4

Energy: -1041.337224245592

C	7.13029435129316	-0.88192419271396	0.42146802935687
C	6.26194869079075	3.63834475697172	1.16507241052550
C	4.51384099544053	-1.70106174178324	1.44355410831367
C	4.92405001001372	-0.31785556362810	1.41427268415194
C	3.25798055386165	-2.01039454307541	1.84983874798319
C	4.44819707743878	2.03464495112462	1.72493804754665
C	4.04106891296784	0.70169928208254	1.78523310159388
C	3.54616496277581	3.10085732101296	2.08744025233983
C	2.33955869445984	-0.97803930075012	2.24184910402983
C	2.71391273201704	0.37611144977600	2.21162037839557
C	2.28893266160643	2.79366441326056	2.49435081172270
C	1.85292107486383	1.43012308798262	2.56140841104035
H	6.64617419827097	-1.35600568730859	-0.43531910883445
H	5.60726470599283	4.16792215354026	0.47032189365301
H	7.99816747649970	-0.32698109675888	0.07578062098779
H	5.20832716063473	-2.47973306387512	1.15431868573433
H	7.25656853317057	3.57447030382714	0.73244850813721
H	7.45153546571804	-1.64091676754549	1.13470616601918
H	2.93703155366416	-3.04546510896714	1.88256719864698
H	3.87477388351244	4.13124959703456	2.04271222049174

H	1.33699651206184	-1.24208530673007	2.56073789277046
H	6.31508088935096	4.17801172721610	2.11120100271519
H	1.60094687925177	3.58423784605903	2.77236132798543
H	0.84141367885952	1.20309242165649	2.88067988277496
N	6.19893724382078	0.05593922557557	1.02879064028003
N	6.68569294906628	1.30422317522711	1.24216667166926
N	5.75697802990998	2.28735812566321	1.35358047650395
H	9.00810309491951	1.91582465531091	1.18961157555200
B	8.33796692380192	1.51601955368966	2.11602616736906
O	8.60086420743136	0.22846321156071	2.65190865948139
O	8.10193244921351	2.42597220134795	3.17395188615214
C	8.67898028568638	0.34977441232384	4.08149529184863
C	7.87501616885041	1.65691090635305	4.36836039010382
C	8.37537894624834	2.45721843167724	5.56079256233038
H	7.74354281830939	3.33888574344940	5.70109446924120
H	8.32753726925662	1.85875476240612	6.47496847175002
H	9.40251885947217	2.79383708726057	5.41591134365641
C	6.37657193156714	1.40537920302308	4.52580699967420
H	6.15602614900213	0.94860712722930	5.49407871208317
H	5.84759561787138	2.36009788434090	4.47263604129168
H	5.98690327910831	0.74980378072354	3.74794699317081
C	8.09709094159849	-0.90186710413359	4.71989217935694
H	8.72907949277597	-1.76239986849200	4.48310502442185
H	8.05942331364534	-0.80032788564331	5.80838333619619
H	7.09079920792459	-1.10793682941007	4.35237214502308
C	10.15617539129542	0.47784014411989	4.44999024246080
H	10.30349624410415	0.50016840690119	5.53269353215764
H	10.69434723209129	-0.38556657706123	4.05006340611940
H	10.59288529850522	1.38204828814752	4.01881140402090

TS3 (Photoexcited) Charge = 0 Multiplicity = 4

Energy: –1040.503011478886

C	7.13651005085236	-0.90969182166852	0.39009913506302
C	6.27830982387392	3.60837841233440	1.24031406625731
C	4.35058884242988	-1.71064120065431	1.01335779145814
C	4.78136253853971	-0.36145428479045	1.11880469944059
C	3.00271151428321	-2.00353480221984	1.23781367189858
C	4.32255710376176	2.04048209430032	1.55963042285212
C	3.87439690935144	0.68840048798005	1.45745557441097
C	3.42830543695067	3.09595848407183	1.88501223985863
C	2.07579652162822	-0.98591560072370	1.57475877497681
C	2.48471106452642	0.37836573991581	1.69390823389669
C	2.08257537598474	2.79075061707811	2.10427247373941
C	1.60625789967362	1.45929642417326	2.01636124388015
H	6.74321232036335	-1.36377002462998	-0.53175570423534
H	5.68827588865441	4.17921632192346	0.50726561968002
H	8.04652569130518	-0.35468307100839	0.13810312406406
H	5.05339833482011	-2.50390308370209	0.75949056178395
H	7.30698061881005	3.53710195466855	0.87007102328178
H	7.37854008868125	-1.71367733182800	1.10280089254986
H	2.66130211163697	-3.03845865832658	1.15019889867677
H	3.78075917334659	4.12423318097964	1.96286455520256
H	1.02539618757003	-1.23811340543700	1.74156512039603
H	6.26933454155111	4.14564440965197	2.20104423815927
H	1.38689854735241	3.59829876531284	2.34769158668546
H	0.54835183222597	1.24548708805007	2.18919735550022
N	6.13610238473158	0.02121940734808	0.89922999262581
N	6.57734832673971	1.21279337640855	1.41702739527748
N	5.71088115157846	2.26890502282453	1.33665343373837
H	8.91506449106996	1.38411409611232	1.63666673628081
B	7.92405637625416	1.27808178137807	2.40572131621082
O	8.25766030420620	0.00042866265861	3.01025401897344
O	7.91694001451771	2.32123308273422	3.36775816372596
C	8.86739490992784	0.33724444537196	4.31853273278983
C	8.13850810999428	1.69505545179375	4.68648739990192
C	8.96952816981807	2.64467538897720	5.54940079711932
H	8.39599434242451	3.56246916945120	5.74454497806815

H	9.20166908009338	2.17865237330531	6.51808480968540
H	9.91133267414670	2.92468865263223	5.06022669722232
C	6.74207365122898	1.48699180241990	5.29924833784481
H	6.81889757613157	1.07084469619287	6.31357488311424
H	6.23322765100797	2.45885612777805	5.36759376785104
H	6.11817113297400	0.81331239576610	4.69414035808950
C	8.57714310511740	-0.83888251777733	5.25799440095138
H	9.12993850617638	-1.72812879820125	4.92371162035658
H	8.91488002635936	-0.58734289633060	6.27304254001063
H	7.50872303536082	-1.08494758169461	5.29498253693355
C	10.38280178660031	0.47083869343619	4.09120741174267
H	10.89712081328222	0.59039936816239	5.05415592520637
H	10.76100007825741	-0.44483806866575	3.61497748487769
H	10.63034488382712	1.32575317246645	3.44756166192457

Conical Intersection After TS3 Charge = 0 Multiplicity = 4

Energy: –1039.407313417097

C	7.24229101594748	-0.88234841799185	0.27777787891920
C	6.39374738529806	3.62279775344397	1.11288698258838
C	4.49150411374863	-1.72520700863589	1.07067788829609
C	4.91670880188796	-0.36219771064449	1.11788467267267
C	3.14651193182061	-2.02151660996704	1.33669873328156
C	4.44648472395333	2.05870654736168	1.50020286785124
C	4.00240506745426	0.69492296576866	1.44565773572727
C	3.54184973724583	3.12537228645802	1.80291918374799
C	2.21220747849974	-0.99412770826673	1.65428464389824
C	2.61366803909659	0.38119833748202	1.71753609072230
C	2.19818469466555	2.81573349252340	2.05419606594263
C	1.72896756412382	1.47148888488026	2.02122780945426
H	6.79639151720955	-1.35436560246540	-0.62172931623512
H	5.78334358923747	4.17719407345487	0.37099250381211
H	8.13323410355599	-0.31051360647919	-0.03758034114794
H	5.20203802469070	-2.52765899636383	0.83086270568853
H	7.42277771686801	3.54653322433210	0.71898474083181
H	7.55192385452033	-1.68677379487204	0.97780905408560
H	2.80878015242920	-3.07005112541983	1.29647065854819
H	3.88988704429641	4.16599626251049	1.84376938286558
H	1.15886625872621	-1.24905576230819	1.85122685472665
H	6.40635826815165	4.19225397489690	2.06501339898222
H	1.49268642316399	3.63145453587435	2.28154872786376
H	0.66869704291067	1.25312263788609	2.22509789675553
N	6.26048199174507	0.03149119285984	0.84855009965277
N	6.70399643501267	1.23339446171466	1.34336830392454
N	5.83333379222156	2.28508928567553	1.26497991287483
H	9.02906980053030	1.39523143349396	1.56719694356266
B	7.98425243464788	1.32297512304666	2.28915976050443
O	8.32468985816377	0.03556118566357	2.91945388111179
O	7.94008101812279	2.34228164043579	3.27986017655584
C	8.90027220339029	0.37151270632056	4.23402442123504
C	8.13278850700030	1.72008435636806	4.58828690206318
C	8.93034260101805	2.67539717862884	5.48459889688728
H	8.32951212629261	3.58564132602461	5.68312075047511
H	9.16111440776968	2.20334657913991	6.46042425468487
H	9.87901206390004	2.99274382028947	5.01221794177895
C	6.73096324333940	1.46888497536875	5.18425257002417
H	6.78704104806234	1.06212836225236	6.21334467424299
H	6.18322550443226	2.43117371866000	5.22772750151978
H	6.13336189847403	0.76711751465157	4.56825053366750
C	8.62702099593925	-0.81991538785641	5.16367027835635
H	9.21594340975103	-1.70108264905493	4.84109029599795
H	8.93742241020350	-0.55917312000740	6.19396099257973
H	7.55851945516680	-1.10343079518655	5.18066463650965
C	10.41961887936825	0.54138277478152	4.02382705237924
H	10.92418784829824	0.63528687316219	5.00365736691967
H	10.82591046746810	-0.35364202601829	3.51286502276986
H	10.65705005017949	1.43068483612734	3.41008100984297

TS3 (High energy) Charge = 0 Multiplicity = 4

Energy: –1041.277243227677

C	6.99392409068851	-0.95018368167839	0.75916457170745
C	6.06263567965033	3.51259341126899	1.63892241110277
C	4.16748575903391	-1.73095966382028	0.90145730939286
C	4.59901702309348	-0.38057143803530	1.17655109179177
C	2.84041684265375	-2.00047303431189	0.88468638993389
C	4.11100111003887	1.95533802028189	1.63406407704610
C	3.67886279751598	0.64438936615383	1.41268488705956
C	3.17164933746701	3.03330987863393	1.83535691195956
C	1.87820914580796	-0.96459339498702	1.12154931424342
C	2.27776823374007	0.35744848934079	1.38266283632103
C	1.84638529682744	2.75525113367470	1.81653371942496
C	1.38011243871467	1.41864888385747	1.58825103429442
H	6.67379455790740	-1.33807632507436	-0.20807593034441
H	5.54716217379649	4.07294502344403	0.85869194945313
H	7.90323072698855	-0.36863856623373	0.62946750933746
H	4.89856075386725	-2.51048446214328	0.73156891917147
H	7.11379959584451	3.41950098863490	1.37859474053945
H	7.16840515772273	-1.77126756312716	1.45385562809760
H	2.49396368107440	-3.00895524519085	0.69005170198707
H	3.52836775834299	4.03918813980572	2.01511260098547
H	0.81955684687678	-1.19729812281174	1.08893361486685
H	5.95784055928350	4.02020853404663	2.59746122596157
H	1.12341115825215	3.54729393824656	1.97420582021605
H	0.31456473273755	1.21898110886150	1.56217800158976
N	5.94371306105067	-0.06008558356772	1.24786231761907
N	6.31888792848258	1.11755746781570	1.81100976918436
N	5.47690553101078	2.17584519109296	1.68342010675380
H	8.93469514421010	1.80879291161409	0.97700977047752
B	7.44309469186191	1.15665984284424	2.80116614759394
O	7.99385201602694	-0.00852781548918	3.23725384287296
O	7.63194619479773	2.23541559270676	3.61037945587464
C	8.84717915350272	0.35120698301933	4.37083513626519
C	8.21996145196644	1.70644552672139	4.84168938842898
C	9.22031037662842	2.71237204141762	5.37518151576161
H	8.69752899202795	3.62199930439888	5.68053016756314
H	9.73141484943234	2.30372799519917	6.25060368829408
H	9.96573158368086	2.97651730754571	4.62496100378796
C	7.06703936814885	1.52573761084473	5.82058992171874
H	7.42680373853295	1.18184151649947	6.79241337251278
H	6.56902307155307	2.48795375432392	5.96034966430602
H	6.33116835707225	0.80966503667594	5.44611532655375
C	8.77015286574333	-0.76543513702257	5.39427484851000
H	9.23401084399263	-1.66666663318228	4.98622935976591
H	9.31477566125389	-0.48237905055527	6.29863320386309
H	7.73992128003663	-0.99882638879979	5.66367081238802
C	10.26953344035012	0.49100894532220	3.84831313978001
H	10.96080179724640	0.69694926969600	4.66813918830286
H	10.56786065494768	-0.44869663916001	3.37725293828899
H	10.35055748850669	1.28632053120042	3.10618657738883

TS4 Charge = 0 Multiplicity = 3

Energy: -1225.574236392572

C	6.19570849617052	-0.75621748182173	0.52951281475473
C	6.10838348634685	2.99190091382393	1.90314323953144
C	3.50017176899732	-1.95174293606787	1.81889524155418
C	4.11701266141226	-0.72616944330455	1.77226447784098
C	2.10823461909115	-2.03076374350864	1.62555182712671
C	4.03259372399289	1.70165618212924	1.48622481746282
C	3.36942843941445	0.43832688467431	1.54504366676274
C	3.33499918992069	2.84232923067606	1.09378807742915
C	1.36239529812643	-0.90428026752384	1.37242480894204
C	1.98412105816021	0.36322809735164	1.31135486744302
C	1.96104139475940	2.74905257602233	0.84242324291692

C	1.28822585565026	1.55439261675084	0.96862728631555
H	5.98120785255770	-1.76674262006438	0.17994469442813
H	6.04029627080336	3.42756666694361	0.90559308419521
H	5.83525789988426	-0.03539364398382	-0.21581362481777
H	4.08889769940872	-2.84702831643621	1.98518482804895
H	7.14059071417789	2.74103767080577	2.12257746464601
H	7.26870665892636	-0.63012645610141	0.67001323577963
H	1.62472966104810	-3.00076081444863	1.66422868265320
H	3.82953826830145	3.80170595657615	1.02374518991284
H	0.29355068533983	-0.97575575682397	1.20179182200954
H	5.70821139978024	3.68541533059776	2.64287696009753
H	1.42121134185585	3.64596454401268	0.56128239415496
H	0.22034525742225	1.50398561830137	0.78494494018382
N	5.53590261704293	-0.58435582615704	1.83203167528668
N	5.87367140696643	0.63010733226063	2.43168235290420
N	5.32925724428006	1.75384913030475	1.90664091510707
B	6.78307744686302	0.64199905905859	3.58511248759868
O	7.21157253774805	-0.52844285233358	4.13594092980413
O	6.96315343542122	1.75221807394207	4.36203844374783
C	7.96846274024088	-0.15790511897093	5.32539453106877
C	7.39458866313597	1.25952446368317	5.66503703680199
C	8.40713346891430	2.22856735708051	6.24463128539906
H	7.92050270701106	3.18408220617924	6.45638147533822
H	8.80961014074016	1.83754371970998	7.18278996975081
H	9.23184765483573	2.40724614654198	5.55471315962920
C	6.14813717390369	1.20553649517485	6.54052115930067
H	6.39385271254752	0.89611170341393	7.55853535614896
H	5.70320860444763	2.20253820344655	6.58354861579246
H	5.40429896275327	0.51526627466031	6.13458496963328
C	7.72400078038771	-1.20651843756865	6.39516198926530
H	8.16280505487750	-2.15699434367860	6.08115395244095
H	8.19928287843928	-0.90715089217413	7.33295626157853
H	6.66003803006220	-1.36222447983529	6.57584283656031
C	9.44238578433881	-0.13196035665745	4.94302340516403
H	10.06527428099482	0.06467067213379	5.81865211384267
H	9.71882906791137	-1.10942552751502	4.54008122276882
H	9.64286939484755	0.62117995981526	4.18172291532960
O	8.60903001820550	0.97764687993666	1.87222509280545
N	9.58634398784746	1.83864506946744	1.85568254596429
N	9.79069850368725	2.80492527950031	2.50591526159553

TS5 Charge = 0 Multiplicity = 3

Energy: -1225.612364092588

C	6.68660938691152	-1.35307387538114	1.10945007215721
C	6.12798758380313	3.27557834549579	1.58941916172103
C	3.89694296687634	-1.82693023513697	1.64149032714942
C	4.42255114294341	-0.55643557224353	1.70460787177130
C	2.50918923417349	-2.00435607004072	1.82532732174488
C	4.12765090172064	1.86290539109701	1.91803174518731
C	3.57447187427918	0.55748916984920	1.92829773871906
C	3.31222214917282	2.96194146134469	2.06558997610808
C	1.67010235600657	-0.94758538008138	2.05395967351219
C	2.18709119365927	0.37292402711279	2.09810779419309
C	1.92655518915094	2.76818462424081	2.24743012068643
C	1.36881667163777	1.51861647439289	2.27225859900650
H	6.79840805629035	-2.11359958499463	1.88319533176114
H	5.62863020313435	3.80760439022847	0.77636746979805
H	6.29166312665340	-1.80773628687988	0.19760011326058
H	4.52905636363914	-2.68660597472122	1.46449538619229
H	7.17184037422223	3.13293331392107	1.32878354630634
H	7.65491117478852	-0.91161324512787	0.89351383716704
H	2.10817744576379	-3.01194143049829	1.78373467938220
H	3.71560505293014	3.96535970124733	2.05737994508907
H	0.60487075240151	-1.10149695793565	2.18929175641784
H	6.05661675313443	3.85832292874968	2.50885239949035
H	1.29586117886425	3.64245061126893	2.37252908417768
H	0.30082914591790	1.38799181235988	2.41041141129249

N	5.77454182126321	-0.30083475664102	1.52716727642658
N	6.31059161750268	0.89868789661829	1.83503514573803
N	5.49895281743475	1.97337080136375	1.73707539053958
B	7.76747111157028	0.99310448406753	2.74747917171370
O	7.94641069730247	-0.25129841743860	3.39899317359134
O	7.62864542108522	2.00989375455706	3.71993261347342
C	7.82028385066512	-0.07318555821253	4.81954105630211
C	7.24832783752722	1.38652185063439	4.95755344200061
C	7.84737605556997	2.19093858026054	6.10111237046897
H	7.38436924022930	3.18116951038103	6.13086977194306
H	7.65875441979577	1.70096896848948	7.06010147216806
H	8.92331018088176	2.32033423802488	5.97878986697114
C	5.72401642585126	1.42467278638251	5.06941485009629
H	5.39321347645996	1.07138204114251	6.04917193015568
H	5.38690107609968	2.45708840719249	4.94821741479110
H	5.23631915355415	0.81178883503423	4.31166865816791
C	6.90015729463017	-1.15919037479560	5.36384143346529
H	7.37637209673856	-2.13474183295109	5.23287935195677
H	6.71616185085077	-1.01481446446632	6.43210302782546
H	5.94208607401422	-1.17793646307929	4.84298357989319
C	9.20414645203400	-0.24688607277965	5.43997328285016
H	9.15488803104008	-0.21741405864446	6.53144564502943
H	9.59856923601477	-1.22282872882961	5.14430896092846
H	9.90082188432378	0.51863733280439	5.10086420517455
O	8.66915010948468	1.28661431963462	1.60444581708123
N	10.06907286426061	1.55866208267142	1.96133845130744
N	10.44712109572659	1.72262752031032	3.03118971764136

TS6 Charge = 0 Multiplicity = 2

C	-4.47677494649245	3.36547878082817	-0.67247905729959
C	-5.45301610823931	3.29494647478349	0.54603552611371
B	-4.42401336485800	1.31126376190018	0.25771923726539
O	-3.65102433214341	2.17553311163566	-0.47488121616959
O	-5.56976522264312	1.85593876361909	0.76819561745137
C	-3.57680262454159	4.58515849218811	-0.69138743452793
H	-2.93730899548393	4.55669569219517	-1.57711284682846
H	-4.18018709284450	5.49557518069395	-0.73702157713808
H	-2.94018706836802	4.63044864219231	0.19249268596854
C	-4.86188483539424	3.89248973328738	1.81677197370390
H	-4.80953449173919	4.98118404875264	1.75051721032761
H	-5.50213457477076	3.63054769638423	2.66226738452312
H	-3.85906551760883	3.50529810908279	2.01310269303555
C	-6.83402886865633	3.86194095032239	0.28210409909625
H	-7.44269931331878	3.78208033368123	1.18619592445656
H	-6.76034679269239	4.91906763742907	0.01390887944638
H	-7.34161827273909	3.33056219480373	-0.52337750139605
C	-5.18316205298791	3.19952639335533	-2.01169607013663
H	-5.77385012743187	4.08477183374643	-2.25643316321013
H	-4.43260712530624	3.06221154711389	-2.79364289274311
H	-5.84361404395119	2.32856899593141	-2.01221762338413
O	-4.02401165638017	-0.00318004801607	0.40131385575656
B	-4.34544645853760	-0.73570584611372	2.35543236791056
O	-3.75437141022169	-1.96126596619468	2.47824688872680
O	-3.87501492652272	0.20722977733237	3.22868021001594
C	-2.59166682351738	-1.76432400009816	3.33801742773740
C	-2.99358840234425	-0.49465867068749	4.15513340997614
C	-2.37956087529173	-3.01339803958991	4.17125494438515
C	-1.39885770204411	-1.52761397661102	2.41912905932697
C	-3.82220411803324	-0.81827854753334	5.39246652548902
C	-1.83679690867252	0.41722222176567	4.51769584216770
H	-2.09462423991582	-3.84392364118612	3.52041979293403
H	-1.57288017702841	-2.85497574487696	4.89194510561158
H	-3.28258632159133	-3.29567497176359	4.71326063444963
H	-0.47444030898820	-1.43333687410045	2.99307317813038
H	-1.29739351861733	-2.38047639864090	1.74381890354294
H	-1.53047506247239	-0.62608659526982	1.81698641106739
H	-3.21042851426870	-1.29293620263145	6.16258855823543

H	-4.22592067685448	0.11135677697015	5.80073999491350
H	-4.65853794237244	-1.47953863237480	5.15269955735244
H	-2.20835516976100	1.26954221098888	5.09212620561623
H	-1.11269243095333	-0.11936684042847	5.13643819206310
H	-1.32757682604457	0.79573469865933	3.63100049346041
H	-5.36096390735101	-0.57374486352750	1.70026534257385
